# Changes in food and drink purchasing behaviour in England during the first 3 months of the COVID-19 pandemic: an interrupted time series analysis

**DOI:** 10.1017/S1368980024001071

**Published:** 2024-11-22

**Authors:** Alexandra Kalbus, Laura Cornelsen, Andrea Ballatore, Steven Cummins

**Affiliations:** 1 Department of Public Health, Environments and Society, London School of Hygiene & Tropical Medicine, 15-17 Tavistock Place, London WC1H 9SH, UK; 2 Department of Digital Humanities, King’s College London, Strand, London WC2R 2LS, UK

**Keywords:** Food and drink purchasing, Ultra-processed foods, COVID-19, Interrupted time series

## Abstract

**Objective:**

This study examined changes food and drink purchasing during the first 3 months of the COVID-19 pandemic in England, and if changes varied by population subgroups.

**Design:**

We investigated changes in take-home food and drink purchasing and frequency of out-of-home (OOH) purchasing using an interrupted time series analysis design. The start of pandemic restrictions (the intervention) was defined as 16 March 2020, when first announced in the UK.

**Setting:**

London and the North of England.

**Participants:**

1245 households reporting take-home and 226 individuals reporting OOH purchases between January 2019 and mid-June 2020 from the GB Kantar Fast Moving Consumer Goods Panel.

**Results:**

The marginal mean estimate of total take-home energy purchased was 17·4 % (95 % CI 14·9, 19·9) higher during the pandemic restriction period compared with the counterfactual. Increases of 35·2 % (95 % CI 23·4, 47·0) in take-home volume of alcoholic beverages and 1·2 % (95 % CI 0·1, 2·4) in foods and drinks high in fat, salt and sugar were observed. Reductions in purchased energy from fruit and vegetables (–7·3 %, 95 % CI –10·9, –3·6), ultra-processed foods (–4·0 %, 95 % CI –5·2, –2·8) and in OOH purchasing frequency (–44·0 %, 95 % CI –58·3, –29·6) were observed. Changes in chocolate and confectionery, soft drink and savoury snack purchases levelled off over time. Changes in all studied outcomes varied by sociodemographic characteristics and usual purchasing.

**Conclusions:**

Pandemic restrictions were associated with positive and negative changes in food and drink purchasing, which differed by individual characteristics. Future research should ascertain if changes persist and translate into changes in health.

Social, public and individual life was severely disrupted by the COVID-19 pandemic. As part of mitigation measures to prevent the spread of COVID-19, the UK Government announced widespread restrictions aimed at minimising transmission on 16 March 2020^(1)^. A nation-wide lockdown was implemented 1 week later, which included the closure of all but ‘essential’ businesses, including the out-of-home (OOH) food sector^(2)^.

The pandemic has had a considerable impact on health behaviours, including changes in daily routines, sleep, smoking, exercise, sedentary behaviour, alcohol consumption and diet^(3–5)^. In the early stages of the pandemic, food shopping shifted to fewer and larger trips^(6)^, with stockpiling also becoming a feature of consumer behaviour^(7)^. Survey findings suggest both health-promoting and health-damaging dietary changes because of pandemic-related restrictions^(8)^. Negative dietary changes included eating out of control, snacking, lower fruit and vegetable consumption, more frequent main meals and greater alcohol consumption^(9,10)^. An extensive analysis of take-home and OOH food and drink purchases in Great Britain showed that total purchased energy increased by 280 kcal per adult per day on average between March and July 2020 compared with the same period in 2019 and by 150 kcal for the remainder of 2020^(11)^.

Positive and negative dietary changes during the pandemic have been observed to vary by age, sex, living arrangements, socio-economic position and usual diet^(8,10–12)^. Among British cohorts, for instance, older cohorts were less likely to change their diets during lockdown, while younger cohorts were more likely to reduce alcohol consumption and increase fruit and vegetable intake^(13)^.

Much of the knowledge on pandemic-related changes in dietary behaviour is based on diet recall surveys and food frequency questionnaires (FFQ) which are subject to recall and social desirability bias^(14)^. To complement existing evidence on dietary changes during the pandemic, this paper makes use of large-scale, objectively collected consumer purchase data. The aim of this study is to examine changes in food and drink purchasing in England following the onset of pandemic restrictions. A secondary aim was to investigate changes across region, sociodemographic characteristics and usual purchasing levels.

## Methods

This study uses an interrupted time series design to estimate changes in food and drink purchasing following the onset of pandemic restrictions. Interrupted time series is used to estimate changes associated with an event by comparing observed post-event outcomes with those calculated by continuing the trend observed prior to the event, that is, the counterfactual^(15)^. We specified the time of the intervention as 16 March 2020 (the ‘interruption’), when pandemic-related restrictions were first announced in the UK. Correspondingly, our study period consisted of 63 pre- and 13 post-intervention weeks. We use the terms ‘pre-pandemic’ and ‘pandemic restrictions’ to refer to the period pre- and post-intervention, respectively.

### Data

#### Data source

Item-level transaction data on take-home and OOH food and drink purchasing were available from households in the Kantar Fast Moving Consumer Goods (FMCG) panel from 1 January 2019 to 11 June 2020. This is a nationally representative sample in terms of household characteristics. Data for this study were available from a previous study and restricted to households residing in London and the North of England (North West, North East, and Yorkshire and the Humber)^(16)^. Within this panel, a subsample of individuals also reports OOH food and drink purchases. Households and individuals who reported food and drink purchasing before and during pandemic restrictions were included in the analysis.

Households in the Kantar FMCG panel record food and drink purchases brought into the home using hand-held barcode scanners and bespoke barcodes for unbarcoded items such as loose produce. Individuals report OOH food and drink purchases via a mobile application. Kantar also provides data on the nutritional content on take-home purchases. Nutritional information for OOH products is unknown unless these are purchased from supermarkets, for example, ready-to-eat meals.

We assumed underreporting when a household did not report take-home purchases for a period of two or more consecutive weeks and removed such household-weeks from the sample^(11)^. Person-weeks from the OOH sample were removed if the individual joined the panel after the start of the study period (but before the onset of pandemic restrictions), and where periods of no recorded OOH purchasing coincided with household underreporting. This resulted in an analytical take-home sample of 89 382 household-weeks and an OOH sample comprising 16 806 person-weeks.

#### Purchase outcomes

We aggregated all purchases to weeks and applied a previously developed classification of thirty-five food groups to the take-home purchases^(17)^. We further determined foods and drinks high in fat, salt and sugar (HFSS) following the Nutrient Profiling Model (NPM)^(18)^, which has been described previously^(19)^, and ultra-processed foods (UPF) following the NOVA classification. Although there is overlap between products categorised as HFSS and UPF, we included both measures because of their different foci (on macronutrients for HFSS, and on level of processing for UPF) and relevance to UK policy. We also determined low-sugar, medium-sugar and high-sugar soft drinks by identifying if products were exempt from the Soft Drinks Industry Levy, or if they were eligible for either the lower or higher levy^(20)^. We examined soft drinks specifically due to their relevance to health and UK policy. We further investigated changes in purchasing of specific food and drink products, namely savoury snacks, chocolate and confectionery and alcoholic beverages as surveys point towards changes in their consumption during the pandemic period^(10)^. See Supplementary Material 1 for details of food and drink classification.

We considered the following take-home purchase outcomes: total energy (kcal); energy (kcal) purchased from fruit and vegetables, HFSS products, UPF, savoury snacks, chocolate and confectionery, low-, medium- and high-sugar soft drinks; volume (ml) of purchased alcoholic beverages; and frequency (days) of OOH purchasing.

#### Covariates

Kantar collects sociodemographic data from the panellists annually. These include sex, age in years and occupational social grade of the main food shopper/OOH reporter, as well as number of adults and presence of children (< 16 years) in the household. Occupational social grade is based on the National Readership Survey classification and further categorised into ‘high’ (AB), ‘middle’ (C1C2) and ‘low’ (DE)^(21)^.

Since food and drink purchasing may be affected by seasonality^(22)^ and pre-pandemic dietary patterns^(23)^, we included several covariates: dummy variables for festivals associated with food, including Valentine’s Day, Easter, Halloween and Christmas; dummy variables for season (quarters of the year); and usual purchasing. The latter was determined along the quartiles of households’/individuals’ average purchasing during mid-March to June in 2019, corresponding to the pandemic restrictions period in 2020. This was done for all outcomes except for alcohol volume and medium- and high-sugar soft drinks, where no four distinct quartiles could be determined, and OOH purchasing, where quartiles led to multicollinearity issues. Instead, usual alcohol purchasing was expressed as tertiles, and medium- and high-sugar soft drink purchasing as well as OOH purchasing as binary variables.

### Statistical analysis

Descriptive statistics were presented as means (sd) and as *n* (%) where appropriate to summarise sample characteristics and unadjusted outcome variables before and during pandemic restrictions.

Because households and OOH reporters did not purchase specific or any food and drink products every week, the percentage of zero values ranged from 4·7 % for total energy purchased to 97·8 % for medium-sugar soft drinks. To account for this zero-inflation, we employed a zero-inflated two-part model^(24)^. Negative binomial distribution was used as outcomes were over-dispersed. We used cluster-robust se to account for clustering of outcomes by household and OOH reporter in all models. Take-home purchase outcomes were expressed as rates: total energy purchased per household member; energy from fruit and vegetables, HFSS products, UPF, savoury snacks, chocolate and confectionery, and soft drinks per total energy; alcohol volume per adult household member. To account for these rates in the count models, respective offsets, that is, log terms with a coefficient of 1, were modelled.

Each outcome was modelled using interrupted time series. Models contained the following variables: time (measured in weeks elapsed since the start of the study and centred at the beginning of the intervention), a dummy variable (‘pandemic restrictions’) indicating the pre- and post-intervention period (level change) and an interaction term that accounted for the trend during pandemic restrictions (time × pandemic restrictions, slope change). Models were adjusted for household characteristics, sociodemographic characteristics of the main food shopper/OOH reporter, region, season and festivals. Model specification was similar in zero and count parts of the models, except for OOH purchasing; due to collinearity, the variables region, presence of children and age group of the main reporter were omitted from the zero component.

We estimated mean weekly household/OOH purchasing and used pairwise comparisons to test the difference in marginal means of purchasing during pandemic restrictions and the counterfactual where restrictions had not happened. This outcome combined the change in both the level and slope of the pandemic restrictions period.

In secondary analyses, we used interaction terms to explore whether changes in food and drink purchasing differed according to (1) region, (2) presence of children in the household, (3) age of the main shopper/OOH reporter (categories < 45, 45–54, 55–46 and 65+ years), (4) occupational social grade of the main shopper/OOH reporter and (5) usual purchasing levels of each outcome. All subgroup analyses were limited by sample size and uneven distributions of households and individuals within categories. Results from these analyses are therefore descriptive and hypothesis-generating.

We present marginalised results relative to the counterfactual in the main paper. Coefficients from underlying models are available in online Supplementary Material 2 (main analysis), 3 (secondary analysis) and 4 (sensitivity analysis). All analyses were conducted in R version 4.1.3.

### Sensitivity analysis

We chose 16 March 2020 as the intervention (‘interruption’) point, which preceded the implementation of lockdown by 1 week, to include an anticipation effect^(6)^. To test this, we ‘moved’ the intervention 1 week later. To assess the implications of assuming true absence of purchasing in household-weeks without reported purchases, we restricted the analysis of take-home purchase outcomes to household-weeks with at least one purchasing occasion (84 955, 95·0 %). We assessed the impact of excluding OOH purchases from household members other than the main reporter on observed findings by repeating the analyses of the OOH sample using purchases from all household members, aggregated to the household level. This increased the number of purchasing occasions by 7·1 %. Finally, two-part models may not be appropriate for panel data regarding assumptions around the nature of zeros^(24)^. Thus, we repeated the analysis using mixed-effects negative binomial models, which account for panel data but not for zero-inflation.

## Results

Our sample comprised 2145 households reporting take-home purchases and 226 OOH-reporting individuals, of whom 43·5 % and 38·5 % resided in London, respectively. Table 1 presents household and individual characteristics of the take-home and OOH sample. While samples were similar overall, there were small differences between the take-home and OOH sample such as lower age among the OOH sample (50·9 *v*. 54·4 years). Table 2 displays the unadjusted mean purchases for the whole study period, as well as before and during pandemic restrictions.

**Table 1.** Descriptive characteristics of the study sample

Characteristic	Sub-category	Take-home (*n* 1245)		OOH (*n* 226)	
		*n*	%	*n*	%
Household characteristics					
Region, *n* (%)	London	541	43·45	87	38·50
	North of England	704	56·55	139	61·50
		Mean	sd	Mean	sd
Number of adults in the household, mean (sd)		2·08	0·89	2·03	0·81
		*n*	%	*n*	%
Children in the household, *n* (%)	Yes	318	25·54	63	27·88
	No	927	74·46	163	72·12
Main food shopper/OOH reporter characteristics					
Sex, *n* (%)	Female	890	71·49	161	71·24
	Male	355	28·51	65	28·76
		Mean	sd	Mean	sd
Age (years), mean (sd)		54·4	13·4	50·9	11·4
		*n*	%	*n*	%
Social grade, *n* (%)	High	271	22·01	48	21·24
	Middle	751	60·32	142	62·83
	Low	220	17·67	36	15·93

OOH, out-of-home.

**Table 2.** Unadjusted purchase outcomes during the whole study period, pre- and post-intervention, mean (sd)

Purchase outcome	Total (76 weeks)		Pre-intervention (63 weeks)		Post-intervention (13 weeks)	
	Mean	sd	Mean	sd	Mean	sd
Take-home purchasing (*n* 1245)						
Weekly energy purchased (kcal)	12 274·04	9423·57	11 874·19	9121·26	14 233·77	10 566·63
Weekly energy from F&V (kcal)	532·34	617·96	521·84	610·80	583·16	649·54
Energy from F&V (%)	4·88	6·94	4·94	7·04	4·59	6·45
Weekly energy from HFSS products (kcal)	6499·67	5991·76	6282·21	5834·70	7564·91	6605·80
Energy from HFSS products (%)	47·85	21·68	47·81	21·78	48·05	21·21
Weekly energy from UPF (kcal)	7133·24	5896·42	6932·00	5725·22	8119·04	6583·81
Energy from UPF (%)	55·86	23·99	56·22	24·07	54·11	23·51
Weekly energy from savoury snacks (kcal)	522·94	905·40	507·60	890·24	598·10	972·79
Energy from savoury snacks (%)	4·16	7·09	4·18	7·21	4·05	6·46
Weekly energy from chocolate and confectionery (kcal)	646·96	1184·79	623·71	1161·75	760·84	1285·70
Energy from chocolate and confectionery (%)	5·06	8·93	5·05	9·09	5·11	8·11
Weekly energy from low-sugar soft drinks (kcal)	45·00	158·46	43·16	156·95	54·01	165·32
Energy from low-sugar soft drinks (%)	0·42	2·32	0·43	2·44	0·40	1·55
Weekly energy from medium-sugar soft drinks (kcal)	4·98	56·94	4·81	55·19	5·78	64·83
Energy from medium-sugar soft drinks (%)	0·04	0·55	0·04	0·49	0·04	0·78
Weekly energy from high-sugar soft drinks (kcal)	27·31	180·38	26·57	170·89	30·90	221·00
Energy from high-sugar soft drinks (%)	0·23	1·82	0·24	1·90	0·19	1·34
Weekly alcoholic beverages per adult household member (ml)	572·48	1715·54	530·55	1605·35	777·88	2164·53
OOH purchasing (*n* 226)						
OOH purchasing occasions (days/week)	1·50	1·74	1·64	1·79	0·84	1·33

F&V, fruit and vegetables; HFSS, high in fat, salt and sugar; OOH, out-of-home; UPF, ultra-processed foods.

Energy is expressed per household member.

### Changes in food and drink purchases

**Figure 1.**
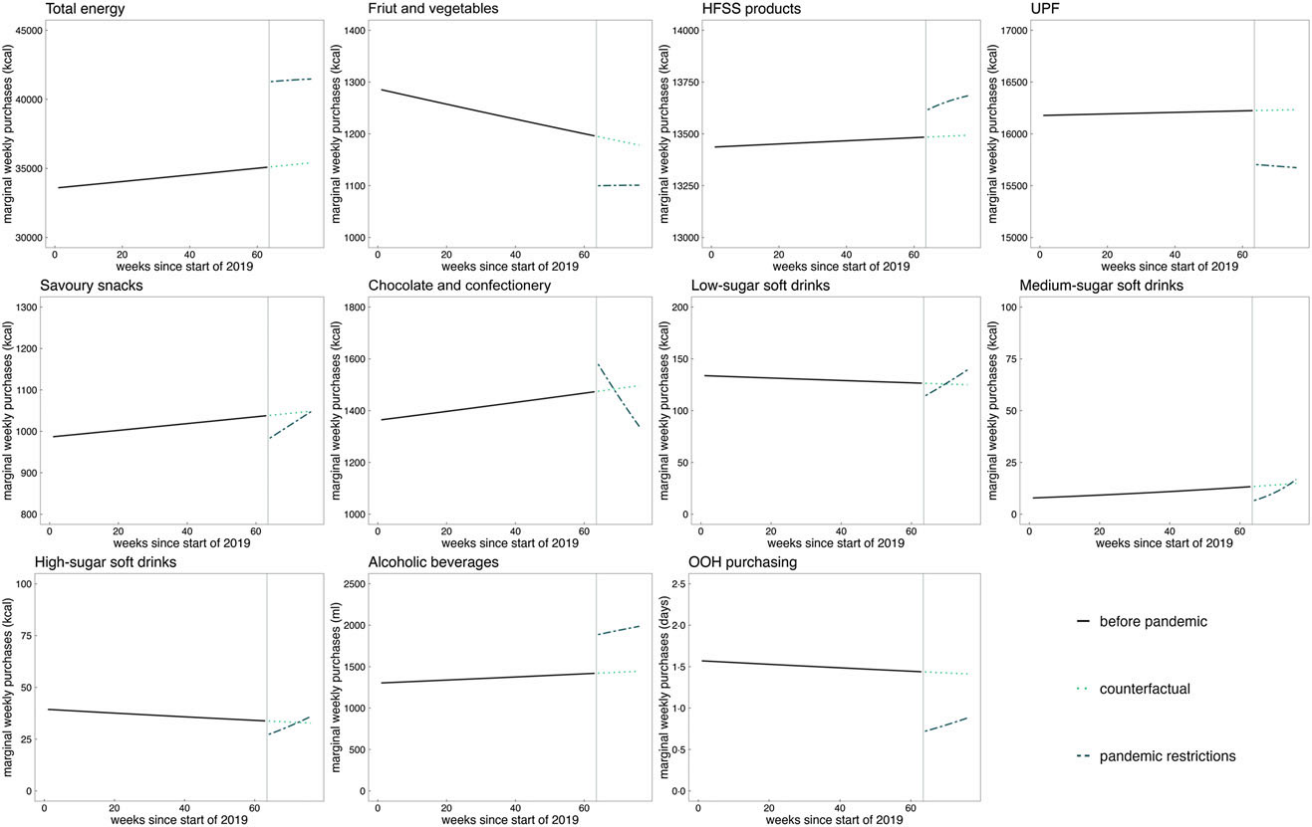
Adjusted weekly mean estimates of food and drink purchasing before and during pandemic restrictions, and the counterfactual. Vertical line = 16 March 2020, start of pandemic restrictions. The counterfactual was estimated by extrapolating the pre-pandemic trend. Marginal means were estimated from interrupted time series two-part models: part 1 (logit) and part 2 (generalised linear model) with negative binomial distribution. Models were adjusted for season, region, festivals, age, sex, and occupational social grade of the main shopper, number of adults, and presence of children. Cluster-robust se were used. Data period: 1 January 2019 to 14 June 2020. Y axes limits were set manually to best display changes; therefore, some do not originate in 0. HFSS, high in fat, salt and sugar; OOH, out-of-home; UPF, ultra-processed foods.

Pandemic restrictions were associated with an increase in average weekly household energy purchased of 6130·2 kcal (95 % CI 5240·2, 7020·2), or 17·4 % (95 % CI 14·9, 19·9), compared with the counterfactual pandemic restrictions had not happened (see Fig. 1 and Table 3). Pandemic restrictions were further linked to reductions in energy purchased from fruit and vegetables of 7·3 % (95 % CI –10·9, –3·6) as well as in energy purchased from UPF of 4·0 % (95 % CI –5·2, –2·8). Compared with the counterfactual, an increase of 164·8 kcal (95 % CI 12·9, 316·8), or 1·2 % (95 % CI 0·1, 2·4), in energy purchased from HFSS products was observed, as well as an increase in purchased volume of alcoholic beverages by 504·9 ml (95 % CI 335·9, 673·8), corresponding to 35·2 % (95 % CI 23·4, 47·0). OOH purchasing frequency fell by 0·6 days per week (95 % CI –0·8, –0·4), corresponding to a reduction of 44·0 % (95 % CI –58·3, –29·6). Pandemic restrictions were associated with a drop in purchasing of energy from fruit and vegetables, UPF, savoury snacks and all types of soft drinks, as well as OOH purchasing (Fig. 1). While energy purchased from fruit and vegetables and UPF as well as OOH purchasing remained lower during pandemic restrictions compared with the counterfactual, energy purchased from savoury snacks and soft drinks increased over the study period to pre-pandemic levels. Post-intervention level increases which persisted during the study period were observed for total energy purchased, energy purchased from HFSS products and purchased alcohol volume. Energy purchased from chocolate and confectionery, although initially higher compared with the counterfactual, decreased over time to below pre-pandemic levels.

**Table 3.** Marginal mean differences, in absolute and relative terms, during pandemic restrictions compared with the counterfactual

Outcome	Measure	Difference in marginal means	95 % CI
Energy purchased	kcal	6130·18	5240·21, 7020·15
	Percent	17·39	14·86, 19·91
Energy from fruit and vegetables	kcal	−85·96	−129·33, –42·59
	Percent	−7·25	−10·90, –3·59
Energy from HFSS products	kcal	164·83	12·86, 316·80
	Percent	1·22	0·10, 2·35
Energy from UPF	kcal	−540·74	−707·23, –374·25
	Percent	−4·01	−5·24, –2·77
Energy from savoury snacks	kcal	−28·06	−76·75, 20·64
	Percent	−2·69	−7·36, 1·98
Energy from chocolate and confectionery	kcal	−29·41	−103·73, 44·91
	Percent	−1·98	−6·98, 3·02
Energy from low-sugar soft drinks	kcal	0·95	−17·66, 19·55
	Percent	0·75	−14·05, 15·56
Energy from medium-sugar soft drinks	kcal	−3·14	−7·99, 1·72
	Percent	−22·24	−56·64, 12·16
Energy from high-sugar soft drinks	kcal	−1·81	−8·59, 4·98
	Percent	−5·43	−25·83, 14·97
Alcohol volume	ml	504·86	335·87, 673·84
	Percent	35·23	23·44, 47·03
OOH purchasing	Occasions	−0·63	−0·83, –0·42
	Percent	−43·95	−58·34, –29·56

HFSS, high in fat, salt and sugar; UPF, ultra-processed foods; OOH, out-of-home.

Models were adjusted for season, region, festivals, age, sex, and occupational social grade of the main shopper, number of adults, and presence of children.

### Subgroup analysis

Full results of the subgroup analysis can be found in Supplementary Material 3 (model coefficients and marginal mean differences, online Supplementary Tables S1–S5). We observed that households with children experienced a greater increase in total energy purchased (22·3 %, 95 % CI 18·1, 26·6 *v*. 15·9 %, 95 % CI 12·8, 18·9) (online Supplementary Material 3, Table S2). They further purchased more energy from HFSS products (3·0 %, 95 % CI 1·4, 4·7), while there was no change observed for households without children. Households without children decreased energy from savoury snacks (–5·7 %, 95 % CI –11·4, –0·1), while households with children did not change savoury snack purchases. Reductions in energy from UPF were greater in households without children (–3·8 %, 95 % CI –5·0, –2·6 *v*. –1·8 %, 95 % CI –3·3, –0·2). On average, the increase of purchased volume of alcoholic beverages was greater for households with children than for those without (64·7 %, 95 % CI 38·2, 89·2 *v*. 28·9 %, 16·3, 41·5).

Age and social grade of the main reporter moderated the association between pandemic restrictions and most purchase outcomes (online Supplementary Material 3, Tables S3 and S4). For instance, main shoppers aged 65 years and older were associated with the smallest increase in total energy purchased during pandemic restrictions compared with other age groups (4·7 %, 95 % CI 0·3, 9·0). Among main shoppers with high social grade, the highest increases in purchased total energy (22·4 %, 95 % CI 16·9, 27·9) and alcoholic beverages (39·1 %, 95 % CI 17·9, 60·2) were observed, alongside the greatest reduction in energy purchased from UPF (–5·0 %, 95 % CI –7·0, –3·1).

Usual purchasing levels moderated the relationship between pandemic restrictions and all purchasing outcomes, with varying directions of the relationship (online Supplementary Material 3, Table S5). For most outcomes, we observed that higher usual purchasing levels were linked to greater reductions during pandemic restrictions, and lower usual purchasing was associated with greater increases during pandemic restrictions. Total energy, for instance, increased in the overall sample, but households with lowest usual purchasing had the largest increase of 41·2 % (95 % CI 35·8, 46·5), while those in the highest quartile did not change total energy purchased during pandemic restrictions. The relative increase in purchasing of alcoholic beverages also followed this pattern, but the absolute increase did not. Higher usual purchasing of alcoholic beverages was linked to a greater absolute increase during pandemic restrictions (lowest tertile 123·2 ml, 95 % CI 71·3, 175·0; highest tertile 708·3 ml, 95 % CI 381·3, 1035·3).

### Sensitivity analyses

Detailed results of the sensitivity analysis can be found in Supplementary Material 4. Results of the analysis which ‘moved’ the intervention 1 week later support our modelling choice: when considering 23 March 2020 as the intervention date, effects of lower magnitude were observed for total energy purchased, and no effect was observed for purchasing of HFSS products (see online Supplementary Material 4, Table S6). When using only household-weeks during which food and drink purchasing occurred, results were similar to those observed in the main analysis which allowed for weeks with zero purchasing. Hence, potential underreporting does not appear to have influenced results. Considering OOH purchasing by all household members and not only of the main reporter led to similar results as when considering the main reporter alone, suggesting that OOH purchasing within the household was similar to the main reporter’s purchasing frequency. Finally, using mixed-effects, instead of two-part, models yielded similar results to those observed in the main analysis, with the exception of UPF: the decrease in UPF energy during pandemic restrictions observed in the main analysis was not replicated in this sensitivity analysis, suggesting that changes in this outcome were dependent on model choice and should be interpreted with caution.

## Discussion

This study, using large-scale objectively collected consumer purchase data with an interrupted time series design, investigated changes in food and drink purchases during the first 13 weeks of pandemic restrictions in England. We found that pandemic restrictions were linked to increases of 17·4 % in total energy purchased, 1·2 % in energy purchased from HFSS products and 35 % in volume of take-home alcoholic beverages compared with the counterfactual where pandemic restrictions had not happened. We found reductions in energy purchased from fruit and vegetables of 7·3 % and UPF of 4·0 %, as well as in the frequency of OOH purchasing frequency of 44·0 %. There were short-lived changes in energy purchased from chocolate and confectionery, savoury snacks and soft drinks which levelled off over the study period and approached pre-pandemic levels towards the end of the observation period. We also observed that changes in food and drink purchasing varied across household sociodemographic characteristics and according to usual purchasing.

### Interpretation of findings

As expected against the backdrop of the closure of the OOH sector for eating-in, the frequency of purchasing for OOH consumption fell from the announcement of pandemic-related restrictions and slowly increased during the study period, while remaining well below pre-pandemic levels. This is in line with previous research^(11)^.

Overall energy purchased was 17·4 % higher in the present study over the study period compared with the counterfactual where pandemic restrictions had not happened, which is also in line with previous investigations^(6)^. It is important to note that the energy estimates presented here do not account for the potential substitution effects from OOH purchasing, hence not reflecting total energy and subsequent consumption. Specifically, it is unknown how much of the observed increase in total energy was attributable to a substitution effect of energy which would have been purchased for OOH consumption. However, O’Connell et al. combined take-home and OOH purchases and reported that purchased energy had increased by 15 % by May 2020 and remained higher during 2020 compared with the pre-pandemic period^(11)^.

Previous research reported that adults in the UK cooked from scratch and consumed healthier meals more often, and reduced purchases of processed foods during the first lockdown^(7,25)^. Our finding of decreased UPF purchasing in the main analysis supports these observations. For many, pandemic restrictions led to more time at home as offices and workplaces as well as opportunities for leisure activities were closed. Time saved could be allocated to food-related activities such as meal preparation and people reported enjoying time spent on taking meals together with household members^(26)^. The observed increase in purchased energy from HFSS foods may have been partly driven by elevated purchasing of ingredients, as products such as table sugar and cooking oils are classified as HFSS^(27)^.

Despite indications of increased home cooking, energy purchased from fruit and vegetables was lower during pandemic restrictions compared with the counterfactual in our study. However, it should be noted that purchased energy from fruit and vegetables was calculated as a function of total energy purchased, that is, the observed decrease refers to the relative energy contribution of fruit and vegetables. It is plausible that the amount of fruit and vegetables purchased by a household did not change or increased at a lower rate than overall energy^(28)^, as fresh produce may be less suitable to stockpile compared products with long shelf lives.

The changes in soft drink purchasing observed in this study, which dropped initially and increased to pre-pandemic levels over the study period, partly reflect prior observations that sugar-sweetened beverage (SSB) consumption decreased during pandemic restrictions^(12)^.

Secondary analyses indicated that changes in purchasing during pandemic restrictions varied by individual characteristics and levels of usual purchasing. The presence of children in the household was associated with greater increases in total energy purchased, indicating increased home cooking as suggested by survey findings^(29)^. On the other hand, households with children also increased purchases of HFSS products and alcoholic beverages during pandemic restrictions more compared with households without children. The latter reported greater decreases in energy from UPF as well as savoury snacks. This reflects differences in the responses by families to pandemic-related restrictions, with some enjoying increased home cooking and spending time with family, and others buying more energy-dense foods, snacks and takeaways^(26,30)^. Greater increases in purchased alcohol consumption of households with children compared with households without have been noted before and linked to stress and anxiety during home confinement^(31)^.

With regard to changes according to age group, Bann et al. report that among British cohort studies, younger cohorts reported more favourable changes with respect to health, while older cohorts reported fewer changes^(13)^. Our findings partly support these observations, as older age groups were overall less likely to change their purchasing.

We found indications that social grade was associated with changes in most of the examined purchasing outcomes. In our study, main shoppers with high social grade increased total take-home energy purchased most during pandemic restrictions compared with lower social grades. This is in line with prior analyses^(6,11)^. Main shoppers with high social grade also saw the largest reduction in energy purchased from UPF and greatest increase in volume of alcoholic beverages for at-home consumption. This may be due to substitution of the OOH sector, given the substantial decrease in OOH purchasing observed in this group (42 %), and that households with high socio-economic status tend to visit restaurants more frequently compared with households with low socio-economic status^(32,33)^.

We observed that changes in purchasing during pandemic restrictions were heavily dependent on usual purchasing. Previously, surveys reported that greater pre-lockdown consumption was associated with an increase in the respective food or drink during lockdown^(12,34)^. Our findings indicate ‘aligning’ effects for all outcomes except alcohol purchasing, with those who usually purchased most reporting the smallest increase or greatest decrease, and vice versa, those usually purchasing least increasing their purchasing most, even though purchasing in this group remained lowest compared with all other households.

Concerningly, absolute changes in alcohol purchasing did not follow this pattern. Purchased volume of take-home alcoholic beverages increased across the full sample, in line with many surveys reporting on increased alcohol consumption during pandemic restrictions^(10,35–37)^. However, Anderson et al. established that while there was an increase in alcohol purchasing of about 40·6 % across the population, this disappeared when adjusting for expected normal purchasing from on-licensed premises, suggesting that missing on-site consumption was offset by increased at-home consumption^(38)^. While there was no change at the population level, prior studies suggest that the heaviest drinkers, an already at-risk population, increased their consumption most^(39,40)^. The alcohol-related mortality rate corroborates these observations: alcohol-related premature mortality increased by 20 % in 2020 compared with 2019, mainly driven by alcoholic liver disease^(40)^, and this trend persisted through 2021^(41)^.

### Implications for future research and policy

The observed increases in total purchases of energy as well as alcohol volume may have negative health consequences. O’Connell et al. estimated that even if purchased energy was back to pre-pandemic levels during 2021, prevalence of overweight would increase by 5 % in 2022^(11)^. A modelling study estimates an additional 207 597 alcohol-attributable hospital admissions and 7153 alcohol-related deaths at an additional cost of £1·1 bn to the NHS by 2042, compared with if alcohol consumption had remained at 2019 levels^(42)^. Future research needs to establish if elevated purchasing and subsequent consumption persist, and if these translate into changes in diet-related health outcomes. Equally, there is a need to ascertain if increased home cooking as observed during pandemic restrictions and indicated by this study’s findings persisted as potentially healthier dietary habits, either population-wide or for some population subgroups. Long-term consequences of reported weight gains during pandemic restrictions linked to decreased exercise and increased food intake and worsened diet quality during pandemic restrictions need to be carefully monitored^(43)^. Pandemic restrictions may have led to improvements in lifestyle and dietary habits of some, but to deteriorations for others, and the long-term health consequences are unclear. A better understanding of these will help inform and target policy interventions.

Potential substitution effects merit further investigation, as home confinement led to shifts in dietary habits. For instance, some eating-out occasions were likely to have been replaced by ordering takeaway food for at-home consumption, as there was steep rise in online food delivery services^(44)^. Another example are snack foods which were usually consumed away from home, for example, at the workplace, and now consumed at home. As a consequence, increased purchasing of respective foods for at-home consumption would be observed, but that does not necessarily translate into greater consumption. While published research explored such substitution effects with regard to energy and alcoholic beverages purchased, as discussed above^(11,38)^, the same could be applied to the dietary health-related outcomes analysed in the present research, including snack foods, HFSS products, UPF and soft drinks.

### Limitations and strengths

A crucial limitation is that we were not able to estimate the total nutritional content of food and drink purchasing, as the available OOH data lack nutritional information. Previously, O’Connell et al. linked Kantar data to other data sources, including the Living Costs and Food Survey, and demonstrated the importance of including OOH purchasing to estimate total diet^(11)^. As the scope of the present study was limited to the Kantar dataset only, we acknowledge this limitation and emphasise that our estimates only indicate shifts in purchasing rather than diets. However, our estimates are still informative as take-home purchasing accounts for most of the total food and drink expenditure^(45)^, and rather than absolute quantities we assessed relative contributions of specific foods and drinks. Since data were available through another study, the present study was restricted to London and the North of England only, and findings cannot be generalised to the whole of England. Further, it is unknown from the household information available whether household composition changed during pandemic restrictions, for example, grown-up children moving back in with their parents. However, there is evidence that household composition remained stable for 95·5 % of households^(46)^. Further, due to available data restricted to London and the North of England, generalisability to Britain overall may be limited. Another limitation relates to the study design, as balanced observations pre- and post-intervention are recommended to maximise statistical power^(15)^. This was not possible as data availability restricted the study period. However, for 63 weeks pre- and 13 weeks post-intervention, even unbalanced, 80 % power to detect small to moderate effects can be expected according to a simulation study^(47)^. Finally, findings based on OOH purchasing models need to be interpreted with caution owing to the small sample size compared with the take-home sample as well as the fact that some subgroup effects could not be modelled in the zero-component due to multicollinearity issues.

The strengths of this study are its use of objectively recorded, granular purchase data as well as its quasi-experimental design^(48)^. Our study does not rely on individual recall and complements the predominantly survey-based evidence on changes in purchasing and consumption following the onset of pandemic restrictions^(10,34,43)^. Furthermore, the detailed nutritional information included in the Kantar data allowed us to investigate changes in food and drink purchasing categories that are current UK policy targets. We furthermore investigated changes in purchasing of UPF, which have been shown to negatively impact dietary health^(49,50)^ but are currently not addressed in UK policies. Previous comprehensive investigations of altered grocery shopping focused on purchases in total as well as broad categories^(6,11)^. In contrast, this study examined purchased energy from specific food groups as a function of total energy, investigating relative changes.

### Conclusions

This study presented an analysis of changes in food and drink purchasing following the onset of restrictions in response to the COVID-19 pandemic in England using large-scale, objectively recorded consumer purchase data and a quasi-experimental design. Pandemic restrictions were associated with abrupt changes in food and drink purchasing, some of which levelled off over time to approach pre-pandemic levels. There were indications that changes in purchasing differed by individual characteristics and usual purchasing habits. Future research needs to ascertain if changes are sustained and whether policy needs to target efforts accordingly to improve population diet.

## Supporting information

Kalbus et al. supplementary material 1 Kalbus et al. supplementary material

Kalbus et al. supplementary material 2 Kalbus et al. supplementary material

Kalbus et al. supplementary material 3 Kalbus et al. supplementary material

Kalbus et al. supplementary material 4 Kalbus et al. supplementary material

## Supplementary material

For supplementary material/s referred to in this article, please visit https://doi.org/10.1017/S1368980024001071

## References

[1] UK Government (2020) Prime Minister’s Statement on Coronavirus (COVID-19): 16 March 2020. https://www.gov.uk/government/speeches/pm-statement-on-coronavirus-16-march-2020 (accessed June 2020).

[2] UK Government (2020) PM Address to the Nation on Coronavirus: 23 March 2020. https://www.gov.uk/government/speeches/pm-address-to-the-nation-on-coronavirus-23-march-2020 (accessed June 2020).

[3] Ruiz-Roso MB , Knott-Torcal C , Matilla-Escalante DC et al. (2020) Covid-19 lockdown and changes of the dietary pattern and physical activity habits in a cohort of patients with type 2 diabetes mellitus. Nutrients 12, 1–16. 10.3390/nu12082327 PMC7468739 32759636

[4] Ferrante G , Camussi E , Piccinelli C et al. (2020) Did social isolation during the SARS-CoV-2 epidemic have an impact on the lifestyles of citizens? Epidemiol Prev 44, 353–362. 33412829 10.19191/EP20.5-6.S2.137

[5] Johnson AN , Clockston RLM , Fremling L et al. (2023) Changes in adults’ eating behaviors during the initial months of the COVID-19 pandemic: a narrative review. J Acad Nutr Diet 123, 144–194.e30. Elsevier. 36075551 10.1016/j.jand.2022.08.132 PMC9444582

[6] Public Health England (2020) Impact of COVID-19 Pandemic on Grocery Shopping Behaviours. London: PHE Publications.

[7] Murphy B , Benson T , Mccloat A et al. (2021) Changes in consumers’ food practices during the COVID-19 lockdown, implications for diet quality and the food system: a cross-continental comparison. Nutrients 13, 20. 10.3390/nu13010020 PMC7822477 33374619

[8] Robinson E , Gillespie S & Jones A (2020) Weight-related lifestyle behaviours and the COVID-19 crisis: an online survey study of UK adults during social lockdown. Obes Sci Pract 6, 735–740. Wiley-Blackwell. 33354349 10.1002/osp4.442 PMC7746963

[9] Ammar A , Brach M , Trabelsi K et al. (2020) Effects of COVID-19 home confinement on eating behaviour and physical activity: results of the ECLB-COVID19 international online survey. Nutrients 12, 1583. MDPI AG. 32481594 10.3390/nu12061583 PMC7352706

[10] Naughton F , Ward E , Khondoker M et al. (2021) Health behaviour change during the UK COVID-19 lockdown: findings from the first wave of the C-19 health behaviour and well-being daily tracker study. Br J Health Psychol 26, 624–643. John Wiley and Sons Ltd. 33410229 10.1111/bjhp.12500 PMC9291054

[11] O’Connell M , Smith K & Stroud R (2022) The dietary impact of the COVID-19 pandemic. J Health Econ 84, 102641. North-Holland. 35689864 10.1016/j.jhealeco.2022.102641 PMC9159790

[12] Lomann M , Claassen MA & Papies EK (2022) The influence of COVID-19 lockdown in the UK on the consumption of sugar-sweetened beverages and water. Appetite 169, 105497. Elsevier.

[13] Bann D , Villadsen A , Maddock J et al. (2021) Changes in the behavioural determinants of health during the COVID-19 pandemic: gender, socioeconomic and ethnic inequalities in five British cohort studies. J Epidemiol Community Health 75, 1136–1142. BMJ Publishing Group Ltd. 34039660 10.1136/jech-2020-215664 PMC8159672

[14] Kirkpatrick SI , Reedy J , Butler EN et al. (2014) dietary assessment in food environment research: a systematic review. Am J Prev Med 46, 94–102. 24355678 10.1016/j.amepre.2013.08.015 PMC4558887

[15] Bernal JL , Cummins S & Gasparrini A (2017) Interrupted time series regression for the evaluation of public health interventions: a tutorial. Int J Epidemiol 46, 348–355. 27283160 10.1093/ije/dyw098 PMC5407170

[16] Cummins S (2019) Protocol ISRCTN - ISRCTN19928803: Restricting Advertising of High Fat, Salt and Sugar Foods and Drinks on the Transport for London Network: Evaluation of a Natural Experiment. Available online 10.1186/ISRCTN19928803 (accessed August 2023).

[17] Berger N , Cummins S , Smith RD et al. (2019) Recent trends in energy and nutrient content of take-home food and beverage purchases in Great Britain: an analysis of 225 million food and beverage purchases over 6 years. BMJ Nutr Prev Health 2, 63. 10.1136/bmjnph-2019-000036 PMC7664498 33235959

[18] Department of Health and Social Care (2011) The Nutrient Profiling Model. https://www.gov.uk/government/publications/the-nutrient-profiling-model (accessed November 2021).

[19] Yau A , Berger N , Law C et al. (2022) Changes in household food and drink purchases following restrictions on the advertisement of high fat, salt, and sugar products across the Transport for London network: a controlled interrupted time series analysis. PLoS Med 19, e1003915. 35176022 10.1371/journal.pmed.1003915 PMC8853584

[20] UK Government (2018) Business Tax: Soft Drinks Industry Levy - Detailed Information. https://www.gov.uk/topic/business-tax/soft-drinks-industry-levy (accessed June 2022).

[21] National Readership Survey (2018) Social Grade. https://www.nrs.co.uk/nrs-print/lifestyle-and-classification-data/social-grade/ (accessed March 2022).

[22] Spence C (2021) Explaining seasonal patterns of food consumption. Int J Gastron Food Sci 24, 100332. Elsevier.

[23] Pérez-Rodrigo C , Citores MG , Bárbara GH et al. (2020) Changes in eating habits during lockdown period due to the COVID-19 pandemic in Spain. Rev Espanola Nutricion Comunitaria 26, 101–111.

[24] Farewell VT , Long DL , Tom BDM et al. (2017) Two-part and related regression models for longitudinal data. Annu Rev Stat Appl 4, 283–315. 28890906 10.1146/annurev-statistics-060116-054131 PMC5590716

[25] Food Standards Agency, Our IM , Mullis V et al. (2020) *Covid-19 Consumer Tracker Waves 5–8.* London: Food Standards Agency.

[26] Scott L & Ensaff H (2022) COVID-19 and the national lockdown: how food choice and dietary habits changed for families in the United Kingdom. Front Nutr 9, 847547. Frontiers. 35685879 10.3389/fnut.2022.847547 PMC9171510

[27] UK Department of Health (2011) Nutrient Profiling Technical Guidance. Available online https://assets.publishing.service.gov.uk/government/uploads/system/uploads/attachment_data/file/216094/dh_123492.pdf (accessed August 2023).

[28] Revoredo-Giha C , Russo C & Twum EK (2022) Purchases of fruit and vegetables for at home consumption during COVID-19 in the UK: trends and determinants. Front Nutr 9, 847996. 35433787 10.3389/fnut.2022.847996 PMC9012448

[29] Bite Back 2030 (2020) Hungry for Change Report. Available online https://urbanhealth.org.uk/insights/reports/hungry-for-change (accessed August 2023).

[30] Porter L , Cox JS , Wright KA et al. (2022) The impact of COVID-19 on the eating habits of families engaged in a healthy eating pilot trial: a thematic analysis. Health Psychol Behav Med 10, 241–261. Routledge. http://mc.manuscriptcentral.com/HPBM 35251773 10.1080/21642850.2022.2043750 PMC8890518

[31] Alcohol Change UK (2020) Drinking in Lockdown Press Release: New Research Reveals that Without Action Lockdown Drinking Habits may be here to Stay. https://alcoholchange.org.uk/blog/2020/drinking-in-the-uk-during-lockdown-and-beyond (accessed October 2022).

[32] Miura K , Giskes K & Turrell G (2012) Socio-economic differences in takeaway food consumption among adults. Public Health Nutr 15, 218–226. Cambridge University Press. 21740620 10.1017/S136898001100139X

[33] Adams J , Goffe L , Brown T et al. (2015) Frequency and socio-demographic correlates of eating meals out and take-away meals at home: cross-sectional analysis of the UK national diet and nutrition survey, waves 1–4 (2008–2012). Int J Behav Nutr Phys Act 12, 51. 25889159 10.1186/s12966-015-0210-8 PMC4404110

[34] Dicken SJ , Mitchell JJ , Newberry Le Vay J et al. (2022) Impact of the COVID-19 pandemic on diet behaviour among UK adults: a longitudinal analysis of the HEBECO study. Front Nutr 8, 788043. 35096934 10.3389/fnut.2021.788043 PMC8793888

[35] EIT Food (2020) COVID-19 Impact on Consumer Food Behaviours in Europe. Available online https://www.eitfood.eu/media/documents/COVID-19_Study_-_European_Food_Behaviours_-_Report.pdf (accessed August 2023).

[36] COVID Symptom Study (2020) The Silent Pandemic: How Lockdown is Affecting Future Health. https://covid.joinzoe.com/post/lockdown-weight-gain (accessed August 2020).

[37] British Nutrition Foundation (2020) BNF Survey Reveals Stress, Anxiety, Tiredness and Boredom are the Main Causes of Unhealthy Eating Habits in Lockdown. https://www.nutrition.org.uk/news/2020/bnf-survey-reveals-stress-anxiety-tiredness-and-boredom-are-the-main-causes-of-unhealthy-eating-habits-in-lockdown/ (accessed August 2022).

[38] Anderson P , Llopis EJ , O’Donnell A et al. (2020) Impact of COVID-19 confinement on alcohol purchases in Great Britain: controlled interrupted time-series analysis during the first half of 2020 compared with 2015–2018. Alcohol Alcohol 56, 307–316. 10.1093/alcalc/agaa128 PMC7717153 33211796

[39] Department of Health and Social Care & Office for National Statistics (2021) Direct and Indirect Health Impacts of COVID-19 in England – Long Paper, 9 September 2021. Available online https://www.gov.uk/government/publications/dhsc-direct-and-indirect-health-impacts-of-covid-19-in-england-long-paper-9-september-2021 (accessed August 2023).

[40] Public Health England (2021) *Monitoring Alcohol Consumption and Harm during the COVID-19 Pandemic.* London: PHE Publications.

[41] Boniface S , Card-Gowers J , Martin A et al. (2022) The COVID Hangover: Addressing Long-Term Health Impacts of Changes in Alcohol Consumption during the Pandemic The COVID Hangover: Addressing Long-Term Health Impacts of Changes in Alcohol Consumption during the Pandemic. London: Institute of Alcohol Studies & HealthLumen.

[42] Angus C , Henney M , Pryce R et al. (2022) Modelling the Impact of Changes in Alcohol Consumption during the COVID-19 Pandemic on Future Alcohol-Related Harm in England. Available online https://orda.shef.ac.uk/articles/report/Modelling_the_impact_of_changes_in_alcohol_consumption_during_the_COVID-19_pandemic_on_future_alcohol-related_harm_in_England/19597249?file=36419202 (accessed August 2023).

[43] Robinson E , Boyland E , Chisholm A et al. (2021) Obesity, eating behavior and physical activity during COVID-19 lockdown: a study of UK adults. Appetite 156, 104853. Elsevier Ltd. 33038479 10.1016/j.appet.2020.104853 PMC7540284

[44] Kalbus A , Ballatore A , Cornelsen L et al. (2023) Associations between area deprivation and changes in the digital food environment during the COVID-19 pandemic: longitudinal analysis of three online food delivery platforms. Health Place 80, 102976. Elsevier Ltd. 36758447 10.1016/j.healthplace.2023.102976 PMC9899780

[45] Cornelsen L , Berger N , Cummins S et al. (2019) Socio-economic patterning of expenditures on ‘out-of-home’ food and non-alcoholic beverages by product and place of purchase in Britain. Soc Sci Med 235, 112361. Elsevier. 31262504 10.1016/j.socscimed.2019.112361

[46] Evandrou M , Falkingham J , Qin M et al. (2020) Changing Living Arrangements, Family Dynamics and Stress during Lockdown: Evidence from Four Birth Cohorts in the UK [Pre-print]. SocArXiv. Available online https://osf.io/preprints/socarxiv/kv8dg (accessed August 2023).

[47] Zhang F , Wagner AK & Ross-Degnan D (2011) Simulation-based power calculation for designing interrupted time series analyses of health policy interventions. J Clin Epidemiol 64, 1252–1261. 21640554 10.1016/j.jclinepi.2011.02.007

[48] Chan GCK , Lim C , Sun T et al. (2022) Causal inference with observational data in addiction research. Addict 117, 2736–2744. John Wiley and Sons Inc. 10.1111/add.15972 PMC9545953 35661462

[49] Elizabeth L , Machado P , Zinöcker M et al. (2020) Ultra-processed food and health outcomes: a narrative review. Nutrients 12, 1955. 32630022 10.3390/nu12071955 PMC7399967

[50] Lane MM , Davis JA , Beattie S et al. (2021) Ultraprocessed food and chronic noncommunicable diseases: a systematic review and meta-analysis of 43 observational studies. Obes Rev 22, e13146. 33167080 10.1111/obr.13146

