# Supplementary material for: Changes in food and drink purchasing behaviour in England during the first 3 months of the COVID-19 pandemic: an interrupted time series analysis
Source: Public Health Nutr. 2024 Nov 22;27(1):e252. doi: 10.1017/S1368980024001071 (PMC11705008; doi:10.1017/S1368980024001071)
Supplement: Kalbus et al. supplementary material 1 — Kalbus et al. supplementary material [file S1368980024001071sup001.pdf]

Supplementary Material 4. Sensitivity Analysis

Sensitivity Analysis 1: setting the intervention time 1 week later to 23rd March 2020

Table S6. Marginal mean differences, in absolute and relative terms, during pandemic restrictions compared to the counterfactual (intervention considered as starting 23rd March 2020)

Outcome	Measure	Difference in marginal means	95% CI
Energy purchased	kcal	5463.97	4537.08, 6390.86
	Percent	15.18	12.60, 17.76
Energy from fruit & vegetables	kcal	-86.60	-131.10, -42.10
	Percent	-0.24	-0.36, -0.12
Energy from HFSS products	kcal	88.04	-67.68, 243.76
	Percent	0.65	-0.50, 1.80
Energy from UPF	kcal	-602.05	-770.60, -433.5
	Percent	-3.71	-4.75, -2.67
Energy from savoury snacks	kcal	-34.43	-83.41, 14.56
	Percent	-3.30	-8.01, 1.40
Energy from chocolate & confectionery	kcal	6.5	-68.43, 81.44
	Percent	0.44	-4.63, 5.51
Energy from low-sugar soft drinks	kcal	-3.20	-21.23, 14.87
	Percent	-2.53	-16.83, 11.77
Energy from medium-sugar soft drinks	kcal	-3.42	-8.20, 1.36
	Percent	-24.91	-59.69, 9.87
Energy from high-sugar soft drinks	kcal	-0.72	-7.47, 6.03
	Percent	-2.23	-23.09, 18.63
Alcohol volume	ml	448.88	285.96, 611.80
	Percent	30.08	19.16, 41.00
OOH purchasing	Occasions	-0.65	-0.86, -0.44
	Percent	-47.03	-62.48, -31.58

HFSS = high in fat, salt and sugar; OOH = out-of-home; UPF = Ultra-processed foods. Models were adjusted for season, region, festivals, age, sex, and occupational social grade of the main shopper, number of adults, and presence of children.

Sensitivity analysis 1: setting the intervention time 1 week later to the 23rd March 2020; all outcomes
HFSS = high in fat, salt and sugar; UPF = ultra-processed food; OOH = out-of-home
Due to multicollineraity, the variables region, presence of children, and age of the main shopper were not included in the OOH models

Outcome	Total Energy purchased					Energy purchased from fruit & vegetables				
Term	Exp. estimate	SE	95%CI low	95%CI high	p value	Exp. estimate	SE	95%CI low	95%CI high	p value
count Constant	15463.682	1.046	15108.884	15826.812	<0.001	0.082	1.089	0.079	0.084	<0.001
count Time	1.001	1.000	1.001	1.001	<0.001	0.999	1.000	0.998	0.999	<0.001
count Pandemic - during pandemic	1.158	1.014	1.124	1.192	<0.001	0.895	1.026	0.860	0.930	<0.001
count Season - 2	1.005	1.006	0.991	1.019	0.427	1.033	1.013	1.013	1.052	0.011
count Season - 3	0.961	1.007	0.948	0.975	<0.001	1.028	1.013	1.009	1.048	0.028
count Season - 4	1.055	1.007	1.040	1.072	<0.001	0.898	1.013	0.880	0.917	<0.001
count Age - 45-54 yrs	1.147	1.029	1.131	1.163	<0.001	0.911	1.051	0.894	0.929	0.061
count Age - 55-64 yrs	1.256	1.032	1.237	1.275	<0.001	0.862	1.060	0.844	0.880	0.010
count Age - 65+ yrs	1.271	1.032	1.251	1.290	<0.001	0.867	1.061	0.849	0.886	0.017
count Sex - male	0.971	1.023	0.961	0.981	0.202	0.971	1.038	0.958	0.985	0.429
count Social grade - C1C2	1.002	1.029	0.990	1.014	0.946	1.145	1.045	1.125	1.164	0.002
count Social grade - AB	0.928	1.034	0.914	0.941	0.027	1.422	1.053	1.394	1.451	<0.001
count - Number of adults	0.864	1.012	0.859	0.868	<0.001	0.890	1.021	0.883	0.896	<0.001
count Presence of children - Yes	0.806	1.026	0.796	0.816	<0.001	0.826	1.046	0.812	0.841	<0.001
count Region - North of England	1.042	1.020	1.033	1.052	0.039	0.757	1.035	0.747	0.767	<0.001
count Festival - Valentine's Day	0.990	1.012	0.961	1.018	0.380	0.929	1.020	0.893	0.966	<0.001
count Festival - Easter	1.046	1.013	1.015	1.079	<0.001	0.987	1.025	0.947	1.029	0.593
countFestival - Halloween	0.922	1.017	0.884	0.962	<0.001	1.067	1.039	1.007	1.131	0.085
count Festival - Christmas	0.853	1.025	0.817	0.890	<0.001	1.077	1.046	1.014	1.144	0.104
count Interaction Time*Pandemic	1.000	1.001	0.996	1.003	0.872	1.002	1.003	0.997	1.007	0.467
zero Constant	0.085	1.174	0.073	0.099	<0.001	0.469	1.198	0.421	0.524	<0.001
zero Time	1.000	1.001	0.998	1.002	0.750	1.000	1.001	0.999	1.001	0.952
zero Pandemic - during pandemic	1.135	1.103	0.936	1.376	0.198	0.802	1.081	0.687	0.936	0.005
zero Season - 2	1.072	1.045	0.974	1.180	0.114	0.985	1.033	0.920	1.055	0.644
zero Season - 3	1.372	1.041	1.255	1.500	0.000	1.045	1.037	0.978	1.118	0.225
zero Season - 4	1.063	1.051	0.957	1.180	0.219	1.189	1.036	1.107	1.276	0.000
zero Age - 45-54 yrs	0.737	1.097	0.677	0.803	0.001	0.834	1.120	0.780	0.891	0.108
zero Age - 55-64 yrs	0.549	1.122	0.499	0.605	<0.001	0.694	1.139	0.646	0.745	0.005
zero Age - 65+ yrs	0.467	1.123	0.422	0.516	<0.001	0.472	1.141	0.438	0.509	<0.001
zero Sex - male	0.871	1.083	0.812	0.935	0.085	1.335	1.094	1.273	1.401	0.001
zero Social grade - C1C2	0.980	1.106	0.900	1.066	0.838	0.631	1.111	0.597	0.667	<0.001
zero Social grade - AB	1.108	1.121	1.005	1.223	0.367	0.550	1.143	0.512	0.591	<0.001
zero - Number of adults	0.922	1.043	0.889	0.957	0.058	0.689	1.066	0.669	0.710	<0.001
zero Presence of children - Yes	0.866	1.092	0.799	0.939	0.102	0.662	1.116	0.620	0.707	<0.001
zero Region - North of England	0.995	1.069	0.936	1.058	0.942	1.107	1.086	1.057	1.158	0.221
zero Festival - Valentine's Day	0.912	1.113	0.737	1.128	0.389	1.105	1.061	0.964	1.266	0.095
zero Festival - Easter	1.312	1.099	1.092	1.576	0.004	0.976	1.081	0.832	1.144	0.751
zeroFestival - Halloween	1.646	1.122	1.301	2.083	<0.001	0.879	1.099	0.718	1.076	0.173
zero Festival - Christmas	2.512	1.103	2.048	3.082	<0.001	1.327	1.093	1.107	1.591	0.002
zero Interaction Time*Pandemic	0.995	1.011	0.972	1.018	0.636	0.992	1.009	0.973	1.012	0.410
Observations	89,382					89,382				

Outcome	Energy purchased from HFSS	Energy purchased from UPF

Term	Exp. estimate	SE	95%CI low	95%CI high	p value	Exp. estimate	SE	95%CI low	95%CI high	p value
count Constant	0.520	1.021	0.513	0.527	<0.001	0.601	1.026	0.593	0.610	<0.001
count Time	1.000	1.000	1.000	1.000	0.392	1.000	1.000	1.000	1.000	0.808
count Pandemic - during pandemic	1.007	1.007	0.990	1.025	0.306	0.961	1.007	0.945	0.977	<0.001
count Season - 2	1.033	1.003	1.025	1.042	<0.001	1.019	1.003	1.011	1.027	<0.001
count Season - 3	1.038	1.004	1.030	1.047	<0.001	1.025	1.003	1.017	1.033	<0.001
count Season - 4	1.072	1.004	1.062	1.081	<0.001	1.027	1.003	1.019	1.036	<0.001
count Age - 45-54 yrs	0.998	1.013	0.990	1.007	0.903	1.019	1.017	1.011	1.027	0.268
count Age - 55-64 yrs	0.993	1.015	0.985	1.002	0.663	0.994	1.020	0.986	1.003	0.775
count Age - 65+ yrs	0.997	1.016	0.988	1.006	0.863	0.985	1.020	0.977	0.994	0.453
count Sex - male	1.006	1.011	1.000	1.013	0.545	0.998	1.013	0.992	1.004	0.887
count Social grade - C1C2	0.971	1.012	0.964	0.978	0.017	0.955	1.015	0.948	0.962	0.002
count Social grade - AB	0.941	1.015	0.932	0.949	<0.001	0.900	1.018	0.893	0.908	<0.001
count - Number of adults	0.992	1.006	0.989	0.995	0.159	0.992	1.007	0.989	0.995	0.212
count Presence of children - Yes	1.015	1.012	1.007	1.023	0.215	1.055	1.015	1.047	1.063	<0.001
count Region - North of England	1.015	1.009	1.010	1.021	0.095	1.059	1.012	1.054	1.065	<0.001
count Festival - Valentine's Day	1.022	1.007	1.004	1.039	0.002	1.015	1.006	0.998	1.032	0.010
count Festival - Easter	0.983	1.007	0.966	1.001	0.019	1.002	1.006	0.984	1.020	0.795
countFestival - Halloween	0.995	1.010	0.970	1.019	0.579	1.012	1.008	0.987	1.036	0.171
count Festival - Christmas	0.993	1.012	0.969	1.019	0.571	0.992	1.011	0.967	1.017	0.418
count Interaction Time*Pandemic	1.000	1.001	0.998	1.002	0.809	1.001	1.001	0.999	1.003	0.305
zero Constant	0.100	1.238	0.080	0.124	<0.001	0.025	1.316	0.018	0.035	<0.001
zero Time	0.998	1.001	0.995	1.001	0.123	0.996	1.002	0.992	1.000	0.061
zero Pandemic - during pandemic	1.560	1.152	1.201	2.026	0.002	1.695	1.227	1.155	2.488	0.010
zero Season - 2	0.944	1.066	0.830	1.074	0.368	0.984	1.109	0.810	1.195	0.873
zero Season - 3	0.965	1.067	0.847	1.099	0.577	1.168	1.101	0.964	1.414	0.106
zero Season - 4	0.816	1.082	0.700	0.950	0.010	0.906	1.116	0.718	1.143	0.370
zero Age - 45-54 yrs	0.620	1.153	0.544	0.707	0.001	0.652	1.238	0.541	0.785	0.044
zero Age - 55-64 yrs	0.520	1.170	0.454	0.597	<0.001	0.487	1.227	0.399	0.595	<0.001
zero Age - 65+ yrs	0.562	1.167	0.493	0.641	<0.001	0.530	1.226	0.438	0.642	0.002
zero Sex - male	1.227	1.115	1.119	1.347	0.060	1.215	1.156	1.061	1.391	0.181
zero Social grade - C1C2	1.059	1.144	0.935	1.200	0.670	1.300	1.190	1.063	1.589	0.131
zero Social grade - AB	1.337	1.166	1.161	1.540	0.059	1.852	1.217	1.488	2.305	0.002
zero - Number of adults	0.663	1.074	0.625	0.704	<0.001	0.788	1.089	0.727	0.854	0.005
zero Presence of children - Yes	0.455	1.147	0.396	0.522	<0.001	0.345	1.195	0.279	0.426	<0.001
zero Region - North of England	0.690	1.103	0.632	0.754	<0.001	0.600	1.131	0.526	0.684	<0.001
zero Festival - Valentine's Day	0.817	1.155	0.609	1.095	0.159	0.987	1.237	0.649	1.501	0.950
zero Festival - Easter	0.935	1.151	0.702	1.246	0.634	1.065	1.219	0.714	1.588	0.750
zeroFestival - Halloween	0.992	1.250	0.638	1.542	0.970	1.024	1.393	0.534	1.965	0.943
zero Festival - Christmas	2.007	1.197	1.430	2.816	<0.001	3.142	1.247	2.060	4.793	<0.001
zero Interaction Time*Pandemic	0.945	1.018	0.914	0.978	0.002	0.974	1.028	0.929	1.020	0.327
Observations	89,382					89,382				

Outcome	Energy purchased from savoury snacks					Enegy purchased from chocolate & confectionery				
Term	Exp. estimate	SE	95%CI low	95%CI high	p value	Exp. estimate	SE	95%CI low	95%CI high	p value
count Constant	0.138	1.077	0.133	0.143	<0.001	0.149	1.064	0.143	0.155	<0.001

count Time	1.000	1.000	1.000	1.001	0.174	1.001	1.000	1.000	1.001	0.079
count Pandemic - during pandemic	0.913	1.028	0.873	0.955	0.001	1.001	1.027	0.954	1.049	0.979
count Season - 2	1.016	1.013	0.994	1.039	0.206	1.036	1.014	1.011	1.061	0.011
count Season - 3	1.067	1.013	1.044	1.091	<0.001	1.049	1.015	1.025	1.075	0.002
count Season - 4	1.081	1.015	1.056	1.107	<0.001	1.180	1.016	1.151	1.210	<0.001
count Age - 45-54 yrs	0.919	1.044	0.900	0.939	0.051	0.997	1.045	0.974	1.021	0.947
count Age - 55-64 yrs	0.813	1.052	0.795	0.833	<0.001	0.937	1.044	0.913	0.961	0.127
count Age - 65+ yrs	0.734	1.060	0.716	0.752	<0.001	0.870	1.046	0.847	0.893	0.002
count Sex - male	1.132	1.037	1.114	1.151	0.001	1.040	1.032	1.022	1.059	0.217
count Social grade - C1C2	0.905	1.041	0.888	0.923	0.014	0.899	1.038	0.880	0.917	0.004
count Social grade - AB	0.921	1.046	0.900	0.943	0.067	0.975	1.045	0.951	1.000	0.573
count - Number of adults	0.898	1.017	0.891	0.905	<0.001	0.872	1.018	0.864	0.880	<0.001
count Presence of children - Yes	0.804	1.043	0.789	0.820	<0.001	0.876	1.039	0.857	0.894	0.001
count Region - North of England	0.880	1.029	0.867	0.892	<0.001	0.984	1.028	0.969	1.000	0.558
count Festival - Valentine's Day	1.081	1.026	1.034	1.130	0.003	1.046	1.030	0.997	1.097	0.125
count Festival - Easter	0.974	1.026	0.929	1.021	0.297	1.184	1.030	1.129	1.241	<0.001
countFestival - Halloween	1.006	1.041	0.942	1.075	0.879	1.123	1.040	1.050	1.201	0.003
count Festival - Christmas	1.243	1.043	1.160	1.332	<0.001	1.170	1.057	1.083	1.265	0.004
count Interaction Time*Pandemic	1.002	1.003	0.997	1.008	0.481	0.986	1.003	0.980	0.991	<0.001
zero Constant	1.706	1.133	1.590	1.830	<0.001	1.614	1.123	1.505	1.731	<0.001
zero Time	0.999	1.001	0.998	1.000	0.123	0.999	1.001	0.998	1.000	0.026
zero Pandemic - during pandemic	0.961	1.044	0.880	1.050	0.362	0.733	1.046	0.671	0.800	<0.001
zero Season - 2	0.916	1.020	0.879	0.955	0.000	0.912	1.022	0.875	0.950	<0.001
zero Season - 3	0.968	1.022	0.929	1.009	0.134	0.946	1.022	0.908	0.986	0.010
zero Season - 4	0.872	1.024	0.833	0.912	<0.001	0.720	1.024	0.688	0.754	<0.001
zero Age - 45-54 yrs	0.882	1.086	0.845	0.920	0.127	0.843	1.082	0.809	0.880	0.031
zero Age - 55-64 yrs	0.922	1.094	0.881	0.965	0.367	0.722	1.090	0.690	0.755	<0.001
zero Age - 65+ yrs	1.223	1.096	1.168	1.280	0.029	0.808	1.090	0.771	0.846	0.013
zero Sex - male	1.011	1.067	0.981	1.043	0.861	1.286	1.063	1.248	1.326	<0.001
zero Social grade - C1C2	0.907	1.081	0.874	0.942	0.210	1.057	1.075	1.018	1.097	0.445
zero Social grade - AB	1.037	1.094	0.992	1.084	0.690	1.250	1.089	1.196	1.306	0.009
zero - Number of adults	0.805	1.034	0.791	0.818	<0.001	0.888	1.033	0.873	0.902	<0.001
zero Presence of children - Yes	0.595	1.080	0.572	0.619	<0.001	0.676	1.075	0.651	0.703	<0.001
zero Region - North of England	0.980	1.057	0.953	1.007	0.714	0.757	1.056	0.737	0.779	<0.001
zero Festival - Valentine's Day	0.871	1.041	0.799	0.950	0.001	0.804	1.040	0.738	0.877	<0.001
zero Festival - Easter	1.023	1.043	0.933	1.121	0.592	0.704	1.045	0.640	0.774	<0.001
zeroFestival - Halloween	1.097	1.059	0.966	1.245	0.106	0.948	1.060	0.834	1.078	0.367
zero Festival - Christmas	1.227	1.064	1.078	1.396	0.001	1.701	1.067	1.495	1.935	<0.001
zero Interaction Time*Pandemic	0.991	1.005	0.980	1.001	0.067	1.015	1.005	1.004	1.026	0.003
Observations	89,382					89,382				

Outcome	Energy purchased from low-sugar soft drinks					Energy from medium-sugar soft drinks				
Term	Exp. estimate	SE	95%CI low	95%CI high	p value	Exp. estimate	SE	95%CI low	95%CI high	p value
count Constant	0.020	1.202	0.019	0.022	<0.001	0.038	1.256	0.030	0.048	<0.001
count Time	0.999	1.001	0.998	1.000	0.418	1.005	1.002	1.001	1.008	0.065
count Pandemic - during pandemic	0.821	1.076	0.746	0.903	0.007	0.601	1.249	0.450	0.803	0.022

count Season - 2	1.015	1.047	0.969	1.064	0.740	0.911	1.088	0.795	1.043	0.267
count Season - 3	1.123	1.044	1.073	1.176	0.007	0.962	1.103	0.841	1.099	0.691
count Season - 4	1.004	1.041	0.953	1.057	0.927	0.915	1.127	0.796	1.052	0.458
count Age - 45-54 yrs	0.738	1.116	0.706	0.771	0.005	1.234	1.186	1.079	1.411	0.218
count Age - 55-64 yrs	0.860	1.149	0.819	0.903	0.278	1.179	1.161	1.015	1.369	0.270
count Age - 65+ yrs	0.674	1.140	0.639	0.710	0.003	0.974	1.178	0.839	1.131	0.874
count Sex - male	1.105	1.095	1.067	1.145	0.269	1.086	1.105	0.981	1.202	0.408
count Social grade - C1C2	0.743	1.124	0.712	0.774	0.011	0.656	1.221	0.579	0.745	0.035
count Social grade - AB	0.621	1.136	0.590	0.654	<0.001	0.685	1.220	0.590	0.795	0.057
count - Number of adults	0.909	1.047	0.895	0.923	0.037	0.845	1.062	0.805	0.886	0.005
count Presence of children - Yes	0.725	1.098	0.697	0.754	0.001	1.114	1.177	0.978	1.268	0.507
count Region - North of England	0.875	1.097	0.847	0.904	0.148	0.940	1.101	0.860	1.027	0.520
count Festival - Valentine's Day	1.084	1.078	0.981	1.198	0.282	1.451	1.234	1.095	1.923	0.076
count Festival - Easter	1.123	1.103	1.017	1.240	0.238	0.834	1.135	0.618	1.126	0.152
countFestival - Halloween	0.983	1.138	0.852	1.134	0.892	1.223	1.298	0.794	1.885	0.439
count Festival - Christmas	1.791	1.239	1.529	2.097	0.007	0.642	1.228	0.454	0.908	0.031
count Interaction Time*Pandemic	1.016	1.008	1.004	1.027	0.052	1.054	1.034	1.019	1.089	0.115
zero Constant	3.347	1.174	3.097	3.617	<0.001	51.839	1.325	40.912	65.684	<0.001
zero Time	1.000	1.001	0.999	1.001	0.444	0.996	1.002	0.993	0.999	0.084
zero Pandemic - during pandemic	0.962	1.046	0.874	1.060	0.396	1.464	1.188	1.079	1.986	0.027
zero Season - 2	0.867	1.022	0.828	0.908	<0.001	0.790	1.077	0.684	0.912	0.002
zero Season - 3	0.807	1.023	0.771	0.844	<0.001	0.841	1.086	0.731	0.968	0.036
zero Season - 4	0.954	1.023	0.908	1.002	0.040	0.726	1.081	0.629	0.837	<0.001
zero Age - 45-54 yrs	0.873	1.113	0.834	0.914	0.204	0.839	1.176	0.730	0.964	0.280
zero Age - 55-64 yrs	1.021	1.124	0.972	1.073	0.859	0.849	1.222	0.731	0.987	0.414
zero Age - 65+ yrs	1.292	1.124	1.228	1.359	0.029	0.984	1.219	0.841	1.151	0.936
zero Sex - male	1.183	1.084	1.144	1.223	0.037	1.289	1.152	1.160	1.432	0.073
zero Social grade - C1C2	0.956	1.104	0.918	0.995	0.647	0.823	1.197	0.723	0.936	0.277
zero Social grade - AB	1.343	1.123	1.279	1.411	0.011	0.844	1.213	0.725	0.982	0.380
zero - Number of adults	0.758	1.046	0.744	0.772	<0.001	0.943	1.076	0.896	0.992	0.423
zero Presence of children - Yes	0.654	1.103	0.627	0.682	<0.001	1.194	1.156	1.048	1.360	0.222
zero Region - North of England	0.559	1.074	0.542	0.576	<0.001	1.242	1.143	1.134	1.359	0.106
zero Festival - Valentine's Day	1.040	1.041	0.946	1.144	0.331	0.899	1.159	0.670	1.206	0.469
zero Festival - Easter	0.946	1.044	0.855	1.046	0.197	1.078	1.157	0.786	1.478	0.609
zeroFestival - Halloween	0.995	1.063	0.866	1.143	0.937	1.516	1.238	0.962	2.391	0.051
zero Festival - Christmas	1.218	1.069	1.056	1.404	0.003	0.916	1.204	0.633	1.325	0.636
zero Interaction Time*Pandemic	0.980	1.005	0.969	0.992	<0.001	0.964	1.017	0.931	0.997	0.032
Observations	89,382					89,382				

Outcome	Energy from high-sugar soft drinks					Alcohol volume				
Term	Exp. estimate	SE	95%CI low	95%CI high	p value	Exp. estimate	SE	95%CI low	95%CI high	p value
count Constant	0.100	1.173	0.089	0.114	<0.001	3771.642	1.171	3533.696	4025.611	<0.001
count Time	0.999	1.001	0.997	1.000	0.246	1.002	1.001	1.001	1.002	0.003
count Pandemic - during pandemic	0.825	1.101	0.696	0.978	0.046	1.113	1.045	1.037	1.194	0.015
count Season - 2	0.929	1.057	0.858	1.006	0.187	1.097	1.022	1.059	1.137	<0.001
count Season - 3	1.154	1.074	1.064	1.252	0.044	1.082	1.027	1.044	1.121	0.003

count Season - 4	0.944	1.067	0.864	1.032	0.380	1.106	1.024	1.065	1.148	<0.001
count Age - 45-54 yrs	0.753	1.114	0.701	0.809	0.009	1.161	1.096	1.117	1.207	0.105
count Age - 55-64 yrs	0.629	1.141	0.583	0.680	<0.001	1.059	1.110	1.017	1.103	0.584
count Age - 65+ yrs	0.665	1.197	0.606	0.729	0.023	0.918	1.130	0.881	0.957	0.487
count Sex - male	1.151	1.104	1.082	1.224	0.156	1.112	1.075	1.084	1.141	0.143
count Social grade - C1C2	0.829	1.115	0.776	0.886	0.087	0.792	1.117	0.767	0.819	0.035
count Social grade - AB	0.795	1.116	0.730	0.865	0.036	0.723	1.121	0.696	0.751	0.005
count - Number of adults	0.854	1.056	0.832	0.876	0.003	0.695	1.038	0.686	0.704	<0.001
count Presence of children - Yes	0.690	1.091	0.645	0.738	<0.001	0.885	1.091	0.853	0.917	0.160
count Region - North of England	0.852	1.097	0.806	0.900	0.082	1.376	1.070	1.343	1.410	<0.001
count Festival - Valentine's Day	0.846	1.088	0.712	1.006	0.048	0.902	1.038	0.838	0.970	0.006
count Festival - Easter	0.945	1.096	0.812	1.099	0.536	1.093	1.040	1.016	1.175	0.025
countFestival - Halloween	0.833	1.119	0.652	1.063	0.103	0.912	1.058	0.822	1.013	0.103
count Festival - Christmas	0.838	1.124	0.675	1.041	0.131	1.034	1.064	0.931	1.147	0.593
count Interaction Time*Pandemic	1.014	1.011	0.994	1.034	0.227	0.997	1.005	0.989	1.005	0.555
zero Constant	10.462	1.332	9.067	12.072	<0.001	8.303	1.189	7.675	8.983	<0.001
zero Time	1.002	1.001	0.999	1.004	0.242	0.999	1.001	0.998	1.000	0.156
zero Pandemic - during pandemic	0.970	1.099	0.804	1.170	0.745	0.808	1.046	0.737	0.886	<0.001
zero Season - 2	0.846	1.049	0.774	0.925	<0.001	0.835	1.023	0.798	0.874	<0.001
zero Season - 3	0.890	1.048	0.812	0.974	0.013	0.822	1.024	0.786	0.860	<0.001
zero Season - 4	0.840	1.054	0.761	0.928	0.001	0.694	1.024	0.661	0.728	<0.001
zero Age - 45-54 yrs	1.691	1.201	1.558	1.835	0.004	0.764	1.125	0.730	0.801	0.022
zero Age - 55-64 yrs	1.792	1.249	1.641	1.957	0.009	0.675	1.135	0.643	0.709	0.002
zero Age - 65+ yrs	3.133	1.268	2.824	3.477	<0.001	0.725	1.138	0.689	0.762	0.013
zero Sex - male	0.888	1.157	0.832	0.949	0.416	1.011	1.092	0.978	1.044	0.903
zero Social grade - C1C2	1.562	1.185	1.453	1.680	0.008	0.782	1.119	0.751	0.815	0.029
zero Social grade - AB	1.992	1.213	1.814	2.189	<0.001	0.801	1.139	0.763	0.840	0.087
zero - Number of adults	0.810	1.078	0.785	0.835	0.005	0.843	1.048	0.828	0.857	<0.001
zero Presence of children - Yes	1.238	1.197	1.147	1.338	0.234	1.337	1.115	1.281	1.396	0.007
zero Region - North of England	1.364	1.141	1.286	1.447	0.018	0.612	1.083	0.594	0.631	<0.001
zero Festival - Valentine's Day	0.961	1.086	0.793	1.166	0.634	0.827	1.039	0.753	0.908	<0.001
zero Festival - Easter	0.720	1.080	0.607	0.854	<0.001	0.981	1.039	0.891	1.080	0.620
zeroFestival - Halloween	1.052	1.125	0.801	1.382	0.666	1.197	1.056	1.047	1.369	0.001
zero Festival - Christmas	0.737	1.126	0.577	0.942	0.010	1.200	1.068	1.049	1.373	0.005
zero Interaction Time*Pandemic	0.991	1.011	0.969	1.013	0.392	0.989	1.005	0.978	0.999	0.017
Observations	89,382					89,382				

Outcome			OOH purchasing		
Term	Exp. estimate	SE	95%CI low	95%CI high	p value
count Constant	1.331	1.352	1.211	1.462	0.343
count Time	0.998	1.001	0.997	0.999	0.011
count Pandemic - during pandemic	0.404	1.121	0.352	0.463	<0.001
count Season - 2	0.990	1.026	0.939	1.043	0.683
count Season - 3	1.014	1.024	0.964	1.067	0.555
count Season - 4	1.018	1.024	0.963	1.077	0.441
count Age - 45-54 yrs	0.912	1.181	0.871	0.956	0.581

count Age - 55-64 yrs	1.046	1.191	0.993	1.102	0.797
count Age - 65+ yrs	0.717	1.261	0.667	0.771	0.152
count Sex - male	1.503	1.137	1.446	1.563	0.001
count Social grade - C1C2	1.025	1.219	0.973	1.081	0.899
count Social grade - AB	1.030	1.301	0.962	1.104	0.910
count - Number of adults	1.005	1.078	0.983	1.028	0.942
count Presence of children - Yes	0.995	1.173	0.950	1.043	0.976
count Region - North of England	1.109	1.125	1.070	1.149	0.380
count Festival - Valentine's Day	1.026	1.027	0.924	1.139	0.327
count Festival - Easter	0.913	1.041	0.805	1.035	0.023
countFestival - Halloween	1.011	1.041	0.871	1.174	0.782
count Festival - Christmas	0.729	1.060	0.617	0.860	<0.001
count Interaction Time*Pandemic	1.044	1.010	1.027	1.061	<0.001
zero Constant	0.073	4.156	0.031	0.169	0.066
zero Time	1.007	1.013	0.993	1.022	0.557
zero Pandemic - during pandemic	3.791	2.975	1.078	13.333	0.222
zero Season - 2	2.299	1.640	1.325	3.988	0.092
zero Season - 3	0.991	1.446	0.548	1.791	0.980
zero Season - 4	1.225	1.463	0.669	2.242	0.594
zero Age - 45-54 yrs	-				
zero Age - 55-64 yrs	-				
zero Age - 65+ yrs	-				
zero Sex - male	8.709	3.683	5.103	14.864	0.097
zero Social grade - C1C2	<0.001	7.257	0.000	Inf	<0.001
zero Social grade - AB	5.727	4.572	3.231	10.150	0.251
zero - Number of adults	0.359	2.170	0.279	0.462	0.186
zero Presence of children - Yes	-				
zero Region - North of England	-				
zero Festival - Valentine's Day	-				
zero Festival - Easter	-				
zeroFestival - Halloween	-				
zero Festival - Christmas	-				
zero Interaction Time*Pandemic	0.750	1.153	0.613	0.918	0.043
Observations	16,806				

Sensitivity analysis 2: including only weeks with take-home purchasing; all take-home purchase outcomes.
Note that as a consequence, total calories were modelled using the same specification as before but with generalised linear models with a negative binomial distribution instead of 2-part models. This is because when considering exclusively weeks with food and drink purchasing, total energy is effectively truncated at values exceeding 0. Since energy from specific foods and drinks may still take on the value of 0, these were continued to be modelled using [illegible] inflated models.

Outcome	Total Energy purchased					Energy purchased from fruit & vegetables				
Term	Exp. estimate	SE	95%CI low	95%CI high	p value	Exp. estimate	SE	95%CI low	95%CI high	p value
count Constant	15129.192	1.047	14776.503	15490.820	<0.001	0.082	1.089	0.080	0.085	<0.001
count Time	1.001	1.000	1.000	1.001	<0.001	0.999	1.000	0.998	0.999	<0.001
count Pandemic - during pandemic	1.194	1.012	1.164	1.225	<0.001	0.907	1.024	0.876	0.938	<0.001
count Season - 2	1.007	1.006	0.993	1.021	0.207	1.029	1.012	1.010	1.048	0.022
count Season - 3	0.970	1.007	0.956	0.983	<0.001	1.025	1.013	1.006	1.045	0.053
count Season - 4	1.070	1.007	1.054	1.087	<0.001	0.894	1.013	0.876	0.913	<0.001
count Age - 45-54 yrs	1.147	1.029	1.131	1.163	<0.001	0.911	1.051	0.894	0.929	0.060
count Age - 55-64 yrs	1.256	1.032	1.237	1.275	<0.001	0.862	1.060	0.844	0.880	0.011
count Age - 65+ yrs	1.271	1.032	1.252	1.291	<0.001	0.867	1.061	0.849	0.885	0.017
count Sex - male	0.971	1.023	0.961	0.981	0.198	0.971	1.038	0.958	0.985	0.432
count Social grade - C1C2	1.002	1.029	0.990	1.014	0.944	1.144	1.045	1.125	1.164	0.002
count Social grade - AB	0.928	1.034	0.914	0.941	0.027	1.422	1.053	1.394	1.451	<0.001
count - Number of adults	0.864	1.013	0.859	0.868	<0.001	0.890	1.021	0.883	0.896	<0.001
count Presence of children - Yes	0.806	1.026	0.796	0.817	<0.001	0.826	1.046	0.812	0.841	<0.001
count Region - North of England	1.042	1.020	1.033	1.052	0.040	0.757	1.035	0.748	0.767	<0.001
count Festival - Valentine's Day	0.998	1.012	0.970	1.027	0.877	0.926	1.020	0.890	0.963	<0.001
count Festival - Easter	1.042	1.013	1.011	1.074	0.001	0.983	1.025	0.944	1.024	0.488
countFestival - Halloween	0.922	1.017	0.884	0.962	<0.001	1.067	1.039	1.008	1.131	0.084
count Festival - Christmas	0.855	1.025	0.819	0.893	<0.001	1.076	1.046	1.013	1.143	0.106
count Interaction Time*Pandemic	0.999	1.001	0.996	1.002	0.326	1.000	1.003	0.996	1.004	0.939
zero Constant	-					0.473	1.198	0.423	0.528	<0.001
zero Time	-					1.000	1.001	0.999	1.002	0.894
zero Pandemic - during pandemic	-					0.842	1.072	0.737	0.962	0.013
zero Season - 2	-					0.977	1.033	0.913	1.046	0.478
zero Season - 3	-					1.040	1.037	0.972	1.113	0.283
zero Season - 4	-					1.182	1.037	1.100	1.271	<0.001
zero Age - 45-54 yrs	-					0.834	1.120	0.780	0.891	0.108
zero Age - 55-64 yrs	-					0.694	1.139	0.646	0.745	0.005
zero Age - 65+ yrs	-					0.472	1.141	0.438	0.509	<0.001
zero Sex - male	-					1.335	1.094	1.273	1.401	0.001
zero Social grade - C1C2	-					0.631	1.111	0.597	0.667	<0.001
zero Social grade - AB	-					0.550	1.143	0.512	0.591	<0.001
zero - Number of adults	-					0.689	1.066	0.669	0.710	<0.001
zero Presence of children - Yes	-					0.662	1.116	0.620	0.707	<0.001
zero Region - North of England	-					1.107	1.086	1.057	1.158	0.221
zero Festival - Valentine's Day	-					1.099	1.061	0.959	1.260	0.112
zero Festival - Easter	-					0.964	1.080	0.823	1.129	0.636
zeroFestival - Halloween	-					0.879	1.099	0.718	1.076	0.173
zero Festival - Christmas	-					1.327	1.093	1.106	1.591	0.002
zero Interaction Time*Pandemic	-					0.987	1.009	0.970	1.004	0.116
Observations	84,955					84,955				

Outcome	Energy purchased from HFSS					Energy purchased from UPF				
Term	Exp. estimate	SE	95%CI low	95%CI high	p value	Exp. estimate	SE	95%CI low	95%CI high	p value

count Constant	0.519	1.021	0.512	0.526	<0.001	0.602	1.026	0.594	0.610	<0.001
count Time	1.000	1.000	1.000	1.000	0.763	1.000	1.000	1.000	1.000	0.756
count Pandemic - during pandemic	1.015	1.006	0.999	1.030	0.022	0.970	1.006	0.955	0.984	<0.001
count Season - 2	1.033	1.003	1.025	1.041	<0.001	1.017	1.003	1.009	1.025	<0.001
count Season - 3	1.039	1.004	1.031	1.048	<0.001	1.024	1.003	1.016	1.032	<0.001
count Season - 4	1.073	1.004	1.064	1.083	<0.001	1.026	1.003	1.017	1.036	<0.001
count Age - 45-54 yrs	0.998	1.013	0.990	1.007	0.901	1.019	1.017	1.011	1.027	0.267
count Age - 55-64 yrs	0.993	1.015	0.985	1.002	0.662	0.995	1.020	0.986	1.003	0.776
count Age - 65+ yrs	0.997	1.016	0.988	1.006	0.861	0.985	1.020	0.977	0.994	0.454
count Sex - male	1.006	1.011	1.000	1.013	0.545	0.998	1.013	0.992	1.004	0.887
count Social grade - C1C2	0.971	1.012	0.964	0.978	0.017	0.955	1.015	0.948	0.962	0.002
count Social grade - AB	0.941	1.015	0.932	0.949	<0.001	0.900	1.018	0.893	0.908	<0.001
count - Number of adults	0.992	1.006	0.989	0.995	0.160	0.992	1.007	0.989	0.995	0.212
count Presence of children - Yes	1.015	1.012	1.007	1.023	0.216	1.055	1.015	1.047	1.063	<0.001
count Region - North of England	1.015	1.009	1.010	1.021	0.095	1.059	1.012	1.054	1.065	<0.001
count Festival - Valentine's Day	1.022	1.007	1.005	1.040	0.001	1.014	1.006	0.997	1.031	0.016
count Festival - Easter	0.982	1.007	0.965	1.000	0.011	0.999	1.006	0.982	1.017	0.914
countFestival - Halloween	0.994	1.010	0.970	1.019	0.573	1.012	1.008	0.987	1.036	0.170
count Festival - Christmas	0.994	1.012	0.969	1.019	0.590	0.991	1.011	0.967	1.017	0.415
count Interaction Time*Pandemic	1.000	1.001	0.998	1.002	0.711	1.000	1.001	0.998	1.002	0.735
zero Constant	0.101	1.238	0.081	0.126	<0.001	0.026	1.314	0.019	0.037	<0.001
zero Time	0.998	1.001	0.995	1.001	0.218	0.997	1.002	0.993	1.001	0.164
zero Pandemic - during pandemic	1.317	1.130	1.043	1.662	0.024	1.337	1.190	0.942	1.898	0.096
zero Season - 2	0.962	1.065	0.846	1.093	0.534	1.008	1.108	0.831	1.222	0.941
zero Season - 3	0.978	1.066	0.858	1.115	0.728	1.176	1.100	0.971	1.425	0.090
zero Season - 4	0.816	1.083	0.699	0.952	0.011	0.896	1.116	0.709	1.133	0.318
zero Age - 45-54 yrs	0.618	1.152	0.542	0.704	0.001	0.653	1.235	0.543	0.786	0.044
zero Age - 55-64 yrs	0.517	1.169	0.451	0.593	<0.001	0.486	1.225	0.398	0.593	<0.001
zero Age - 65+ yrs	0.557	1.166	0.488	0.635	<0.001	0.526	1.224	0.435	0.637	0.002
zero Sex - male	1.228	1.115	1.119	1.347	0.059	1.222	1.155	1.067	1.399	0.164
zero Social grade - C1C2	1.052	1.143	0.929	1.191	0.707	1.281	1.187	1.049	1.564	0.149
zero Social grade - AB	1.331	1.165	1.156	1.533	0.061	1.833	1.214	1.475	2.278	0.002
zero - Number of adults	0.666	1.073	0.628	0.706	<0.001	0.791	1.088	0.730	0.857	0.006
zero Presence of children - Yes	0.453	1.146	0.394	0.520	<0.001	0.348	1.192	0.282	0.429	<0.001
zero Region - North of England	0.690	1.102	0.632	0.754	<0.001	0.598	1.131	0.525	0.681	<0.001
zero Festival - Valentine's Day	0.817	1.155	0.608	1.096	0.160	0.981	1.237	0.645	1.494	0.930
zero Festival - Easter	0.990	1.146	0.746	1.314	0.944	1.161	1.210	0.785	1.717	0.433
zeroFestival - Halloween	0.987	1.250	0.635	1.535	0.953	1.017	1.392	0.530	1.950	0.959
zero Festival - Christmas	1.993	1.197	1.420	2.796	<0.001	3.094	1.247	2.029	4.718	<0.001
zero Interaction Time*Pandemic	0.961	1.016	0.932	0.991	0.013	0.994	1.024	0.952	1.037	0.788
Observations	84,955					84,955				

Outcome	Energy purchased from savoury snacks					Enegy purchased from chocolate & confectionery				
Term	Exp. estimate	SE	95%CI low	95%CI high	p value	Exp. estimate	SE	95%CI low	95%CI high	p value
count Constant	0.138	1.077	0.133	0.144	<0.001	0.150	1.064	0.144	0.156	<0.001
count Time	1.000	1.000	1.000	1.001	0.173	1.001	1.000	1.000	1.001	0.021

count Pandemic - during pandemic	0.932	1.025	0.896	0.970	0.004	0.966	1.025	0.925	1.007	0.161
count Season - 2	1.012	1.013	0.991	1.034	0.334	1.039	1.014	1.015	1.063	0.005
count Season - 3	1.065	1.013	1.042	1.089	<0.001	1.046	1.016	1.022	1.072	0.003
count Season - 4	1.079	1.015	1.053	1.105	<0.001	1.173	1.016	1.144	1.204	<0.001
count Age - 45-54 yrs	0.919	1.044	0.900	0.939	0.051	0.997	1.045	0.974	1.021	0.948
count Age - 55-64 yrs	0.813	1.052	0.795	0.832	<0.001	0.936	1.044	0.913	0.960	0.126
count Age - 65+ yrs	0.734	1.060	0.716	0.752	<0.001	0.870	1.046	0.847	0.893	0.002
count Sex - male	1.132	1.037	1.114	1.151	0.001	1.040	1.032	1.022	1.059	0.214
count Social grade - C1C2	0.906	1.041	0.888	0.924	0.014	0.899	1.038	0.881	0.917	0.004
count Social grade - AB	0.921	1.046	0.900	0.943	0.067	0.975	1.045	0.951	1.000	0.574
count - Number of adults	0.898	1.017	0.891	0.905	<0.001	0.872	1.018	0.865	0.880	<0.001
count Presence of children - Yes	0.804	1.043	0.789	0.820	<0.001	0.876	1.039	0.857	0.895	0.001
count Region - North of England	0.880	1.029	0.867	0.892	<0.001	0.984	1.028	0.969	1.000	0.560
count Festival - Valentine's Day	1.078	1.026	1.031	1.127	0.004	1.043	1.030	0.994	1.094	0.154
count Festival - Easter	0.969	1.026	0.924	1.015	0.208	1.192	1.030	1.137	1.249	<0.001
countFestival - Halloween	1.006	1.041	0.942	1.075	0.879	1.124	1.040	1.051	1.202	0.003
count Festival - Christmas	1.243	1.043	1.160	1.332	<0.001	1.169	1.057	1.081	1.263	0.005
count Interaction Time*Pandemic	1.000	1.003	0.995	1.005	0.989	0.988	1.003	0.983	0.994	<0.001
zero Constant	1.710	1.133	1.592	1.836	<0.001	1.638	1.123	1.526	1.759	<0.001
zero Time	0.999	1.001	0.998	1.000	0.151	0.999	1.001	0.998	1.000	0.069
zero Pandemic - during pandemic	0.966	1.038	0.895	1.044	0.361	0.768	1.041	0.711	0.829	<0.001
zero Season - 2	0.915	1.020	0.878	0.953	<0.001	0.901	1.021	0.865	0.939	<0.001
zero Season - 3	0.967	1.022	0.928	1.008	0.123	0.937	1.022	0.899	0.977	0.002
zero Season - 4	0.870	1.024	0.831	0.911	0.000	0.712	1.024	0.680	0.746	<0.001
zero Age - 45-54 yrs	0.882	1.086	0.845	0.920	0.127	0.843	1.082	0.808	0.879	0.030
zero Age - 55-64 yrs	0.922	1.094	0.881	0.965	0.366	0.721	1.090	0.689	0.755	<0.001
zero Age - 65+ yrs	1.222	1.096	1.167	1.280	0.029	0.807	1.090	0.771	0.845	0.013
zero Sex - male	1.011	1.067	0.981	1.043	0.861	1.286	1.063	1.248	1.326	<0.001
zero Social grade - C1C2	0.907	1.081	0.874	0.942	0.210	1.057	1.075	1.018	1.097	0.446
zero Social grade - AB	1.037	1.094	0.992	1.084	0.690	1.249	1.089	1.195	1.306	0.009
zero - Number of adults	0.805	1.034	0.792	0.818	<0.001	0.888	1.033	0.874	0.903	<0.001
zero Presence of children - Yes	0.595	1.080	0.572	0.619	<0.001	0.676	1.075	0.650	0.703	<0.001
zero Region - North of England	0.980	1.057	0.953	1.007	0.714	0.757	1.056	0.737	0.779	<0.001
zero Festival - Valentine's Day	0.870	1.041	0.798	0.949	0.001	0.797	1.040	0.731	0.869	<0.001
zero Festival - Easter	1.021	1.043	0.932	1.119	0.615	0.695	1.045	0.633	0.764	<0.001
zeroFestival - Halloween	1.097	1.059	0.967	1.245	0.106	0.949	1.060	0.835	1.078	0.371
zero Festival - Christmas	1.227	1.064	1.078	1.397	0.001	1.700	1.067	1.494	1.934	<0.001
zero Interaction Time*Pandemic	0.990	1.005	0.980	1.000	0.030	1.009	1.005	0.999	1.019	0.052
Observations	84,955					84,955				

Outcome	Energy purchased from low-sugar soft drinks					Energy from medium-sugar soft drinks				
Term	Exp. estimate	SE	95%CI low	95%CI high	p value	Exp. estimate	SE	95%CI low	95%CI high	p value
count Constant	0.021	1.205	0.019	0.022	<0.001	0.038	1.256	0.030	0.048	<0.001
count Time	0.999	1.001	0.998	1.000	0.419	1.004	1.002	1.001	1.007	0.076
count Pandemic - during pandemic	0.871	1.070	0.801	0.948	0.042	0.697	1.219	0.542	0.896	0.067
count Season - 2	1.005	1.046	0.960	1.053	0.908	0.889	1.087	0.778	1.017	0.160

count Season - 3	1.119	1.045	1.068	1.172	0.010	0.952	1.103	0.832	1.089	0.615
count Season - 4	1.000	1.042	0.949	1.054	0.991	0.908	1.126	0.788	1.046	0.416
count Age - 45-54 yrs	0.738	1.116	0.706	0.772	0.006	1.242	1.188	1.086	1.420	0.208
count Age - 55-64 yrs	0.860	1.149	0.819	0.903	0.278	1.185	1.162	1.021	1.376	0.258
count Age - 65+ yrs	0.674	1.140	0.639	0.710	0.003	0.984	1.180	0.848	1.142	0.921
count Sex - male	1.105	1.095	1.067	1.144	0.271	1.077	1.105	0.973	1.192	0.456
count Social grade - C1C2	0.743	1.124	0.713	0.774	0.011	0.656	1.221	0.578	0.744	0.035
count Social grade - AB	0.622	1.136	0.590	0.654	<0.001	0.690	1.219	0.594	0.801	0.061
count - Number of adults	0.909	1.047	0.895	0.923	0.037	0.843	1.062	0.803	0.884	0.004
count Presence of children - Yes	0.725	1.098	0.697	0.754	0.001	1.126	1.179	0.989	1.281	0.474
count Region - North of England	0.875	1.097	0.848	0.904	0.148	0.939	1.101	0.859	1.026	0.510
count Festival - Valentine's Day	1.080	1.079	0.977	1.194	0.309	1.439	1.233	1.084	1.909	0.083
count Festival - Easter	1.106	1.104	1.003	1.221	0.304	0.802	1.132	0.596	1.080	0.075
countFestival - Halloween	0.983	1.138	0.852	1.133	0.891	1.218	1.295	0.790	1.878	0.445
count Festival - Christmas	1.791	1.239	1.529	2.098	0.007	0.640	1.229	0.452	0.906	0.031
count Interaction Time*Pandemic	1.009	1.007	0.999	1.020	0.186	1.038	1.031	1.008	1.069	0.224
zero Constant	3.390	1.175	3.134	3.668	<0.001	50.709	1.325	39.895	64.455	<0.001
zero Time	1.000	1.001	0.999	1.001	0.793	0.996	1.002	0.993	0.999	0.069
zero Pandemic - during pandemic	0.926	1.040	0.851	1.008	0.053	1.426	1.165	1.087	1.871	0.020
zero Season - 2	0.868	1.022	0.830	0.908	<0.001	0.799	1.076	0.694	0.921	0.002
zero Season - 3	0.803	1.023	0.767	0.840	<0.001	0.852	1.086	0.740	0.981	0.051
zero Season - 4	0.946	1.024	0.900	0.995	0.018	0.737	1.082	0.638	0.853	<0.001
zero Age - 45-54 yrs	0.873	1.113	0.834	0.915	0.205	0.839	1.176	0.730	0.964	0.280
zero Age - 55-64 yrs	1.021	1.124	0.972	1.073	0.858	0.849	1.222	0.731	0.987	0.414
zero Age - 65+ yrs	1.292	1.124	1.228	1.359	0.029	0.984	1.219	0.842	1.151	0.936
zero Sex - male	1.183	1.084	1.144	1.223	0.037	1.288	1.152	1.159	1.432	0.074
zero Social grade - C1C2	0.956	1.104	0.918	0.995	0.647	0.823	1.197	0.723	0.936	0.277
zero Social grade - AB	1.343	1.123	1.279	1.411	0.011	0.844	1.213	0.725	0.982	0.380
zero - Number of adults	0.758	1.046	0.744	0.772	<0.001	0.943	1.076	0.896	0.992	0.423
zero Presence of children - Yes	0.654	1.103	0.627	0.682	<0.001	1.194	1.156	1.048	1.360	0.221
zero Region - North of England	0.559	1.074	0.542	0.576	<0.001	1.242	1.143	1.134	1.359	0.106
zero Festival - Valentine's Day	1.035	1.041	0.941	1.139	0.392	0.910	1.158	0.679	1.222	0.522
zero Festival - Easter	0.952	1.044	0.862	1.052	0.259	1.087	1.156	0.794	1.489	0.563
zeroFestival - Halloween	0.996	1.063	0.867	1.144	0.942	1.516	1.238	0.962	2.392	0.051
zero Festival - Christmas	1.216	1.069	1.054	1.401	0.004	0.918	1.203	0.635	1.327	0.642
zero Interaction Time*Pandemic	0.983	1.005	0.973	0.993	<0.001	0.968	1.016	0.938	0.999	0.040
Observations	84,955					84,955				

Outcome		Energy from high-sugar soft drinks					Alcohol volume			
Term	Exp. estimate	SE	95%CI low	95%CI high	p value	Exp. estimate	SE	95%CI low	95%CI high	p value
count Constant	0.102	1.174	0.090	0.116	<0.001	3672.106	1.172	3436.500	3923.867	<0.001
count Time	0.999	1.001	0.997	1.001	0.369	1.001	1.001	1.000	1.002	0.057
count Pandemic - during pandemic	0.810	1.092	0.695	0.944	0.017	1.171	1.041	1.099	1.248	<0.001
count Season - 2	0.926	1.056	0.856	1.002	0.161	1.098	1.022	1.060	1.137	<0.001
count Season - 3	1.145	1.074	1.055	1.243	0.057	1.093	1.027	1.055	1.133	0.001
count Season - 4	0.933	1.068	0.852	1.021	0.291	1.125	1.024	1.083	1.170	<0.001

count Age - 45-54 yrs	0.753	1.114	0.701	0.808	0.008	1.161	1.096	1.116	1.206	0.105
count Age - 55-64 yrs	0.630	1.141	0.583	0.680	<0.001	1.060	1.110	1.017	1.104	0.579
count Age - 65+ yrs	0.667	1.198	0.608	0.732	0.025	0.919	1.130	0.881	0.958	0.490
count Sex - male	1.151	1.104	1.082	1.224	0.156	1.112	1.075	1.084	1.141	0.143
count Social grade - C1C2	0.830	1.115	0.777	0.887	0.088	0.792	1.117	0.766	0.818	0.034
count Social grade - AB	0.796	1.116	0.731	0.866	0.037	0.723	1.121	0.696	0.751	0.005
count - Number of adults	0.854	1.055	0.832	0.876	0.003	0.695	1.038	0.686	0.704	<0.001
count Presence of children - Yes	0.690	1.091	0.645	0.738	<0.001	0.885	1.091	0.853	0.917	0.160
count Region - North of England	0.852	1.097	0.806	0.900	0.082	1.375	1.070	1.342	1.409	<0.001
count Festival - Valentine's Day	0.839	1.088	0.706	0.998	0.038	0.911	1.038	0.847	0.981	0.012
count Festival - Easter	0.946	1.093	0.814	1.100	0.533	1.084	1.040	1.008	1.166	0.040
countFestival - Halloween	0.834	1.119	0.653	1.064	0.106	0.911	1.058	0.821	1.011	0.098
count Festival - Christmas	0.836	1.124	0.674	1.039	0.126	1.037	1.064	0.934	1.151	0.561
count Interaction Time*Pandemic	1.014	1.011	0.995	1.033	0.218	0.995	1.005	0.987	1.002	0.265
zero Constant	10.398	1.333	8.987	12.031	<0.001	7.752	1.192	7.149	8.405	<0.001
zero Time	1.001	1.001	0.999	1.003	0.304	1.000	1.001	0.999	1.001	0.436
zero Pandemic - during pandemic	1.002	1.086	0.846	1.186	0.985	0.794	1.041	0.731	0.863	<0.001
zero Season - 2	0.844	1.048	0.773	0.922	<0.001	0.825	1.023	0.789	0.864	<0.001
zero Season - 3	0.891	1.048	0.813	0.976	0.014	0.794	1.025	0.758	0.831	<0.001
zero Season - 4	0.843	1.054	0.763	0.933	0.001	0.678	1.025	0.645	0.712	<0.001
zero Age - 45-54 yrs	1.691	1.201	1.558	1.835	0.004	0.780	1.127	0.745	0.818	0.038
zero Age - 55-64 yrs	1.792	1.249	1.641	1.957	0.009	0.702	1.138	0.668	0.738	0.006
zero Age - 65+ yrs	3.133	1.268	2.824	3.477	<0.001	0.761	1.141	0.723	0.801	0.038
zero Sex - male	0.888	1.157	0.832	0.949	0.416	1.020	1.093	0.987	1.054	0.824
zero Social grade - C1C2	1.562	1.185	1.453	1.680	0.008	0.781	1.121	0.749	0.814	0.030
zero Social grade - AB	1.992	1.213	1.813	2.189	<0.001	0.790	1.141	0.752	0.830	0.074
zero - Number of adults	0.810	1.078	0.785	0.835	0.005	0.844	1.049	0.830	0.859	<0.001
zero Presence of children - Yes	1.238	1.197	1.147	1.337	0.234	1.358	1.117	1.301	1.419	0.006
zero Region - North of England	1.364	1.141	1.286	1.447	0.018	0.607	1.084	0.589	0.626	<0.001
zero Festival - Valentine's Day	0.963	1.087	0.794	1.168	0.649	0.822	1.039	0.747	0.903	<0.001
zero Festival - Easter	0.715	1.078	0.603	0.847	<0.001	0.955	1.039	0.867	1.053	0.237
zeroFestival - Halloween	1.052	1.125	0.801	1.382	0.668	1.157	1.057	1.009	1.325	0.008
zero Festival - Christmas	0.738	1.126	0.577	0.943	0.011	1.090	1.069	0.950	1.252	0.197
zero Interaction Time*Pandemic	0.988	1.010	0.968	1.008	0.225	0.987	1.004	0.977	0.997	0.003
Observations	84,955					84,955				

Sensitivity analysis 3: including OOH purchasing from all household members instead of the (known) main shopper only; OOH purchasing

Although the total number of household-weeks did not change, the number of purchase transactions was 27,037, which was 7.1% higher than the 25,235 reported by the main reporters in the household and included in the mai analysis.

OOH = out-of-home. Due to multicollineraity, the variables region, presence of children, and age of the main shopper were not included in the OOH models

	OOH purchasing				
Term	Exp. estimate	SE	95%CI low	95%CI high	p value
count Constant	1.337	1.340	1.219	1.468	0.321
count Time	0.998	1.001	0.997	0.999	0.003
count Pandemic - during pandemic	0.497	1.080	0.446	0.553	<0.001
count Season - 2	0.952	1.022	0.905	1.001	0.027
count Season - 3	0.991	1.022	0.943	1.041	0.662
count Season - 4	0.995	1.022	0.941	1.051	0.806
count Age - 45-54 yrs	0.935	1.175	0.893	0.979	0.677
count Age - 55-64 yrs	1.084	1.182	1.031	1.141	0.629
count Age - 65+ yrs	0.722	1.247	0.672	0.774	0.139
count Sex - male	1.518	1.133	1.462	1.576	0.001
count Social grade - C1C2	1.003	1.219	0.953	1.056	0.987
count Social grade - AB	1.021	1.296	0.956	1.092	0.935
count - Number of adults	1.033	1.078	1.011	1.056	0.665
count Presence of children - Yes	0.967	1.165	0.925	1.012	0.828
count Region - North of England	1.161	1.122	1.121	1.202	0.196
count Festival - Valentine's Day	1.005	1.026	0.908	1.112	0.850
count Festival - Easter	0.886	1.039	0.786	0.999	0.001
countFestival - Halloween	1.001	1.041	0.866	1.157	0.983
count Festival - Christmas	0.760	1.055	0.648	0.891	<0.001
count Interaction Time*Pandemic	1.022	1.007	1.008	1.036	0.003
zero Constant	0.105	3.435	0.049	0.224	0.067
zero Time	1.008	1.013	0.995	1.022	0.522
zero Pandemic - during pandemic	2.252	1.969	0.883	5.747	0.231
zero Season - 2	2.243	1.621	1.330	3.782	0.095
zero Season - 3	0.965	1.409	0.546	1.706	0.918
zero Season - 4	1.306	1.403	0.741	2.301	0.431
zero Age - 45-54 yrs	-				
zero Age - 55-64 yrs	-				
zero Age - 65+ yrs	-				
zero Sex - male	7.926	3.252	4.867	12.908	0.079
zero Social grade - C1C2	<0.001	38.974	0.000	22976938.731	0.028
zero Social grade - AB	4.574	4.441	2.754	7.597	0.308
zero - Number of adults	0.345	2.144	0.270	0.441	0.163
zero Presence of children - Yes	-				
zero Region - North of England	-				
zero Festival - Valentine's Day	-				
zero Festival - Easter	-				
zeroFestival - Halloween	-				
zero Festival - Christmas	-				
zero Interaction Time*Pandemic	0.833	1.096	0.730	0.951	0.046
Observations	16,806				

Sensitivity analysis 4: using mixed effects negative binomial models instead of 2-part models; all outcomes
HFSS = high in fat, salt and sugar; UPF = ultra-processed food; OOH = out-of-home

Outcome	Total Energy purchased					Energy purchased from fruit & vegetables				
Term	Exp. estimate	SE	95%CI low	95%CI high	p value	Exp. estimate	SE	95%CI low	95%CI high	p value
Constant	9437.811	372.704	8734.879	10197.311	<0.001	668.298	46.837	582.525	766.700	<0.001
Time	1.015	0.005	1.004	1.025	0.007	0.985	0.007	0.970	0.999	0.037
Pandemic - during pandemic	1.186	0.093	1.016	1.384	0.031	0.870	0.095	0.702	1.078	0.202
Season - 2	1.005	0.011	0.984	1.026	0.662	1.031	0.016	1.000	1.061	0.046
Season - 3	0.952	0.010	0.932	0.972	<0.001	0.973	0.015	0.945	1.003	0.078
Season - 4	1.062	0.013	1.037	1.087	<0.001	0.941	0.016	0.910	0.973	<0.001
Age - 45-54 yrs	1.173	0.036	1.104	1.247	<0.001	1.078	0.060	0.967	1.202	0.174
Age - 55-64 yrs	1.298	0.044	1.215	1.387	<0.001	1.147	0.069	1.020	1.291	0.022
Age - 65+ yrs	1.343	0.046	1.255	1.436	<0.001	1.272	0.078	1.128	1.435	<0.001
Sex - male	0.969	0.022	0.926	1.013	0.168	0.849	0.035	0.784	0.920	<0.001
Social grade - C1C2	1.015	0.028	0.961	1.073	0.591	1.295	0.065	1.174	1.428	<0.001
Social grade - AB	0.929	0.031	0.870	0.991	0.026	1.508	0.089	1.343	1.694	<0.001
Number of adults	0.879	0.009	0.861	0.897	<0.001	1.237	0.024	1.191	1.284	<0.001
Presence of children - Yes	0.816	0.024	0.771	0.863	<0.001	1.359	0.070	1.229	1.503	<0.001
Region - North of England	1.052	0.022	1.010	1.096	0.015	0.890	0.033	0.827	0.957	0.002
Festival - Valentine's Day	1.004	0.023	0.960	1.050	0.867	0.937	0.030	0.881	0.997	0.041
Festival - Easter	1.027	0.024	0.981	1.077	0.256	1.028	0.034	0.963	1.097	0.406
countFestival - Halloween	0.900	0.030	0.844	0.960	0.001	0.964	0.044	0.881	1.054	0.418
Festival - Christmas	0.808	0.027	0.757	0.862	<0.001	0.843	0.039	0.770	0.923	<0.001
Interaction Time*Pandemic	1.004	0.056	0.900	1.120	0.945	1.219	0.094	1.048	1.417	0.010
Observations	89,382					89,382				
Groups (households/individuals)	1,245					1,245				

Outcome	Energy purchased from HFSS					Energy purchased from UPF				
Term	Exp. estimate	SE	95%CI low	95%CI high	p value	Exp. estimate	SE	95%CI low	95%CI high	p value

Constant	9195.136	442.237	8367.967	10104.071	<0.001	9682.282	482.023	8782.162	10674.659	<0.001
Time	1.014	0.006	1.002	1.027	0.022	1.014	0.006	1.003	1.025	0.015
Pandemic - during pandemic	1.245	0.114	1.040	1.490	0.017	1.161	0.097	0.986	1.368	0.073
Season - 2	1.037	0.013	1.011	1.063	0.004	1.023	0.012	1.000	1.046	0.048
Season - 3	0.985	0.013	0.961	1.010	0.238	0.973	0.011	0.951	0.995	0.017
Season - 4	1.148	0.016	1.117	1.180	<0.001	1.098	0.014	1.070	1.126	<0.001
Age - 45-54 yrs	1.106	0.042	1.027	1.192	0.008	1.150	0.045	1.065	1.242	<0.001
Age - 55-64 yrs	1.239	0.051	1.143	1.343	<0.001	1.263	0.054	1.162	1.374	<0.001
Age - 65+ yrs	1.288	0.054	1.186	1.398	<0.001	1.296	0.056	1.190	1.412	<0.001
Sex - male	0.935	0.026	0.885	0.988	0.016	0.922	0.027	0.871	0.977	0.006
Social grade - C1C2	0.999	0.034	0.934	1.068	0.971	0.996	0.035	0.929	1.068	0.917
Social grade - AB	0.887	0.036	0.819	0.960	0.003	0.861	0.036	0.793	0.935	<0.001
Number of adults	1.270	0.017	1.238	1.303	<0.001	1.262	0.017	1.229	1.296	<0.001
Presence of children - Yes	1.496	0.053	1.396	1.603	<0.001	1.573	0.057	1.464	1.690	<0.001
Region - North of England	1.114	0.028	1.060	1.171	<0.001	1.187	0.031	1.127	1.250	<0.001
Festival - Valentine's Day	1.024	0.027	0.972	1.078	0.377	1.010	0.024	0.963	1.059	0.681
Festival - Easter	1.021	0.028	0.967	1.078	0.457	1.033	0.026	0.984	1.086	0.192
countFestival - Halloween	0.892	0.034	0.828	0.961	0.003	0.907	0.032	0.847	0.970	0.005
Festival - Christmas	0.815	0.031	0.756	0.879	<0.001	0.792	0.028	0.739	0.848	<0.001
Interaction Time*Pandemic	0.986	0.064	0.868	1.119	0.823	1.006	0.059	0.896	1.129	0.922
Observations	89,382					89,382				
Groups (households/individuals)	1,245					1,245				

Outcome	Energy purchased from savoury snacks					Energy purchased from chocolate & confectionery				
Term	Exp. estimate	SE	95%CI low	95%CI high	p value	Exp. estimate	SE	95%CI low	95%CI high	p value
Constant	593.673	62.391	483.161	729.463	<0.001	679.607	64.217	564.712	817.878	<0.001
Time	1.025	0.016	0.993	1.057	0.124	1.031	0.017	0.999	1.064	0.059
Pandemic - during pandemic	1.014	0.236	0.642	1.602	0.952	2.583	0.614	1.622	4.114	<0.001
Season - 2	1.086	0.035	1.020	1.157	0.010	1.090	0.036	1.022	1.163	0.009
Season - 3	1.029	0.034	0.966	1.097	0.373	1.008	0.034	0.944	1.076	0.812
Season - 4	1.340	0.049	1.248	1.439	<0.001	1.568	0.058	1.458	1.686	<0.001
Age - 45-54 yrs	1.152	0.095	0.980	1.353	0.086	1.160	0.086	1.004	1.341	0.044
Age - 55-64 yrs	1.181	0.106	0.991	1.407	0.062	1.371	0.110	1.171	1.604	<0.001
Age - 65+ yrs	0.853	0.078	0.713	1.019	0.080	1.233	0.101	1.051	1.448	0.010
Sex - male	0.942	0.057	0.836	1.062	0.329	0.800	0.044	0.718	0.890	<0.001
Social grade - C1C2	1.119	0.083	0.968	1.294	0.129	0.911	0.060	0.800	1.037	0.159
Social grade - AB	0.986	0.087	0.830	1.171	0.872	0.845	0.067	0.724	0.986	0.033
Number of adults	1.302	0.037	1.232	1.376	<0.001	1.200	0.031	1.142	1.261	<0.001
Presence of children - Yes	1.711	0.131	1.473	1.987	<0.001	1.554	0.107	1.358	1.777	<0.001
Region - North of England	1.021	0.056	0.917	1.137	0.703	1.273	0.063	1.155	1.402	<0.001
Festival - Valentine's Day	1.144	0.077	1.002	1.306	0.047	1.125	0.077	0.983	1.288	0.086
Festival - Easter	1.000	0.070	0.871	1.148	1.000	1.545	0.111	1.342	1.779	<0.001
countFestival - Halloween	0.794	0.077	0.656	0.961	0.018	0.999	0.099	0.822	1.213	0.990
Festival - Christmas	0.990	0.098	0.816	1.202	0.922	0.695	0.070	0.571	0.847	<0.001
Interaction Time*Pandemic	1.130	0.186	0.818	1.561	0.458	0.623	0.104	0.448	0.865	0.005
Observations	89,382					89,382				
Groups (households/individuals)	1,245					1,245				

Outcome	Energy purchased from low-sugar soft drinks					Energy from medium-sugar soft drinks				
Term	Exp. estimate	SE	95%CI low	95%CI high	p value	Exp. estimate	SE	95%CI low	95%CI high	p value
Constant	23.210	4.594	15.747	34.210	<0.001	0.299	0.164	0.102	0.877	0.028
Time	0.994	0.017	0.961	1.028	0.731	1.131	0.135	0.895	1.429	0.303

Pandemic - during pandemic	0.584	0.146	0.357	0.954	0.032	0.362	0.639	0.011	11.558	0.565
Season - 2	1.115	0.039	1.041	1.193	0.002	1.791	0.423	1.127	2.846	0.014
Season - 3	1.414	0.050	1.319	1.516	<0.001	1.446	0.348	0.903	2.317	0.125
Season - 4	1.203	0.047	1.114	1.300	<0.001	2.600	0.695	1.539	4.390	<0.001
Age - 45-54 yrs	1.107	0.174	0.814	1.506	0.516	1.612	0.652	0.729	3.563	0.238
Age - 55-64 yrs	1.075	0.183	0.770	1.501	0.669	0.977	0.440	0.404	2.362	0.958
Age - 65+ yrs	0.844	0.146	0.601	1.186	0.329	1.007	0.460	0.411	2.467	0.988
Sex - male	0.733	0.085	0.584	0.921	0.008	0.638	0.199	0.346	1.176	0.150
Social grade - C1C2	0.946	0.134	0.717	1.247	0.693	1.980	0.781	0.914	4.289	0.083
Social grade - AB	0.550	0.092	0.396	0.764	<0.001	2.654	1.208	1.088	6.475	0.032
Number of adults	1.528	0.082	1.374	1.698	<0.001	1.592	0.227	1.205	2.105	0.001
Presence of children - Yes	1.809	0.263	1.360	2.406	<0.001	1.136	0.427	0.544	2.372	0.734
Region - North of England	1.979	0.208	1.610	2.432	<0.001	0.960	0.266	0.558	1.652	0.883
Festival - Valentine's Day	1.009	0.075	0.872	1.166	0.908	1.301	0.634	0.500	3.383	0.590
Festival - Easter	1.134	0.086	0.978	1.315	0.096	0.773	0.399	0.281	2.124	0.617
countFestival - Halloween	0.811	0.086	0.659	0.999	0.048	0.542	0.427	0.116	2.537	0.437
Festival - Christmas	1.169	0.125	0.948	1.441	0.143	2.236	1.758	0.479	10.440	0.306
Interaction Time*Pandemic	1.937	0.344	1.368	2.744	<0.001	2.132	2.661	0.185	24.603	0.544
Observations	89,382					89,382				
Groups (households/individuals)	1,245									

Outcome		Energy from high-sugar soft drinks						Alcohol volume		
Term	Exp. estimate	SE	95%CI low	95%CI high	p value	Exp. estimate	SE	95%CI low	95%CI high	p value
Constant	0.144	0.160	0.016	1.282	0.082	9.029	3.604	4.129	19.743	<0.001
Time	0.919	0.070	0.791	1.068	0.273	1.008	0.026	0.958	1.061	0.753
Pandemic - during pandemic	0.075	0.080	0.009	0.602	0.015	0.616	0.229	0.297	1.278	0.193
Season - 2	1.521	0.227	1.136	2.037	0.005	1.360	0.070	1.230	1.504	<0.001
Season - 3	1.678	0.261	1.236	2.277	0.001	1.485	0.078	1.339	1.646	<0.001
Season - 4	2.536	0.441	1.803	3.567	<0.001	2.649	0.161	2.351	2.984	<0.001
Age - 45-54 yrs	0.195	0.173	0.034	1.113	0.066	2.991	0.946	1.610	5.560	0.001
Age - 55-64 yrs	0.025	0.023	0.004	0.150	<0.001	3.201	1.099	1.633	6.275	0.001
Age - 65+ yrs	0.009	0.008	0.002	0.052	<0.001	2.591	0.907	1.304	5.145	0.007
Sex - male	0.910	0.485	0.320	2.584	0.859	0.839	0.196	0.531	1.326	0.452
Social grade - C1C2	0.354	0.233	0.098	1.285	0.114	2.427	0.692	1.388	4.244	0.002
Social grade - AB	0.421	0.331	0.090	1.969	0.272	2.327	0.786	1.200	4.513	0.012
Number of adults	5.858	1.489	3.560	9.640	<0.001	1.254	0.137	1.013	1.553	0.037
Presence of children - Yes	1.974	1.619	0.396	9.852	0.407	1.130	0.332	0.636	2.009	0.676
Region - North of England	1.069	0.524	0.409	2.795	0.892	3.655	0.774	2.414	5.534	<0.001
Festival - Valentine's Day	1.147	0.360	0.619	2.123	0.663	1.348	0.150	1.084	1.677	0.007
Festival - Easter	1.897	0.587	1.035	3.478	0.038	1.188	0.133	0.954	1.480	0.124
countFestival - Halloween	0.429	0.183	0.186	0.988	0.047	0.621	0.102	0.450	0.857	0.004
Festival - Christmas	744.808	487.240	206.635	2684.635	<0.001	3.494	0.696	2.365	5.163	<0.001
Interaction Time*Pandemic	8.930	6.805	2.006	39.762	0.004	2.266	0.599	1.350	3.806	0.002
Observations	89,382					89,382				
Groups (households/individuals)	1,245					1,245				

Outcome		OOH purchasing			
Term	Exp. estimate	SE	95%CI low	95%CI high	p value
Constant	0.933	0.226	0.580	1.501	0.776
Time	0.975	0.009	0.958	0.992	0.004
Pandemic - during pandemic	0.251	0.045	0.177	0.355	<0.001
Season - 2	0.950	0.018	0.915	0.985	0.006

Season - 3	1.004	0.018	0.969	1.041	0.817
Season - 4	0.983	0.020	0.944	1.023	0.400
Age - 45-54 yrs	1.070	0.181	0.767	1.491	0.691
Age - 55-64 yrs	1.204	0.226	0.833	1.740	0.324
Age - 65+ yrs	0.853	0.207	0.530	1.374	0.513
Sex - male	1.341	0.182	1.028	1.750	0.030
Social grade - C1C2	1.103	0.190	0.787	1.546	0.568
Social grade - AB	0.813	0.169	0.541	1.222	0.320
Number of adults	1.029	0.063	0.912	1.161	0.645
Presence of children - Yes	0.921	0.152	0.666	1.273	0.618
Region - North of England	1.074	0.136	0.838	1.376	0.573
Festival - Valentine's Day	1.012	0.039	0.939	1.091	0.751
Festival - Easter	0.896	0.043	0.814	0.985	0.023
countFestival - Halloween	1.027	0.056	0.923	1.143	0.626
Festival - Christmas	0.715	0.046	0.629	0.812	<0.001
Interaction Time*Pandemic	1.741	0.215	1.368	2.217	<0.001
Observations	16,806				
Groups (households/individuals)	226				
