# Supplementary material for: Changes in food and drink purchasing behaviour in England during the first 3 months of the COVID-19 pandemic: an interrupted time series analysis
Source: Public Health Nutr. 2024 Nov 22;27(1):e252. doi: 10.1017/S1368980024001071 (PMC11705008; doi:10.1017/S1368980024001071)
Supplement: Kalbus et al. supplementary material 2 — Kalbus et al. supplementary material [file S1368980024001071sup002.docx]

**Supplementary Material 1. Definition of analysed food and drink categories.**

| **Category** | **Description** |
| --- | --- |
| Fruit and vegetables | Fresh, frozen, tinned and dried fruit, fresh, frozen and tinned vegetables (excluding legumes and potatoes) |
| HFSS products | Food and drink products classified as HFSS according to the NPM^1^ |
| UPF | Food and drink products classified as UPF according to the NOVA classification^2^ |
| Savoury snacks | Crisps, popcorn, savoury crackers and biscuits, pork scratchings, poppadoms and prawn crackers |
| Chocolate and confectionery | Chocolate confectionery, sugar confectionery and sweet spreads (e.g. jams and chocolate spreads) |
| Soft drinks* | Soft drinks potentially eligible for the SDIL^3^, including products that are either ready to drink or to be diluted with water, and have added sugar (excluding alcohol-replacement drinks, fruit juice and milk-based drinks) |
| Low-sugar soft drinks | Soft drinks with sugar content < 5 g/100 ml (no levy) |
| Medium-sugar soft drinks | Soft drinks with sugar content 5-8 g/100 ml (lower levy) |
| High-sugar soft drinks | Soft drinks with sugar content > 8 g/100 ml (higher levy) |
| Alcohol | All alcoholic beverages (excluding non-alcoholic drinks) |
| HFSS = high in fat, salt and sugar; NPM = nutrient profiling model; UPF = ultra-processed foods; SDIL = Soft Drinks Industry Levy.  *Where eligible products were intended to be diluted such as cordials, we applied the manufacturer’s dilution advice to determine the drink’s levy status. We classified soft drinks exclusively based on their sugar content, while in reality, small producers (i.e. producing less than 1 million litres of liable drinks annually) are exempt from the levy^3^.  ^1^UK Department of Health 2011. Nutrient Profiling Technical Guidance. London.  ^2^Monteiro CA, Cannon G, Levy RB, Moubarac JC, Louzada MLC, Rauber F, et al. Ultra-processed foods: What they are and how to identify them. Public Health Nutr. 2019;22:936–41.  ^3^UK Government. (2018). *Business tax: Soft Drinks Industry Levy - detailed information*. https://www.gov.uk/topic/business-tax/soft-drinks-industry-levy | |
