# Supplementary material for: Changes in food and drink purchasing behaviour in England during the first 3 months of the COVID-19 pandemic: an interrupted time series analysis
Source: Public Health Nutr. 2024 Nov 22;27(1):e252. doi: 10.1017/S1368980024001071 (PMC11705008; doi:10.1017/S1368980024001071)
Supplement: Kalbus et al. supplementary material 3 — Kalbus et al. supplementary material [file S1368980024001071sup003.pdf]

Model coefficients from main analysis; main effetcs
HFSS = high in fat, salt and sugar; UPF = ultra-processed food; OOH = out-of-home
Due to multicollineraity, the variables region, presence of children, and age of the main shopper were not included in the OOH models

Outcome	Total Energy purchased					Energy purchased from fruit & vegetables				
Term	Exp. estimate	SE	95%CI low	95%CI high	p value	Exp. estimate	SE	95%CI low	95%CI high	p value
count Constant	15131.362	1.046	14778.568	15492.577	<0.001	0.082	1.089	0.080	0.085	<0.001
count Time	1.001	1.000	1.000	1.001	<0.001	0.999	1.000	0.998	0.999	<0.001
count Pandemic - during pandemic	1.194	1.012	1.164	1.225	<0.001	0.907	1.024	0.876	0.938	<0.001
count Season - 2	1.007	1.006	0.993	1.021	0.209	1.029	1.012	1.010	1.048	0.022
count Season - 3	0.970	1.007	0.956	0.983	<0.001	1.025	1.013	1.006	1.045	0.053
count Season - 4	1.071	1.007	1.054	1.087	<0.001	0.894	1.013	0.876	0.913	<0.001
count Age - 45-54 yrs	1.147	1.029	1.131	1.163	<0.001	0.911	1.051	0.894	0.929	0.060
count Age - 55-64 yrs	1.255	1.032	1.237	1.274	<0.001	0.862	1.060	0.844	0.880	0.011
count Age - 65+ yrs	1.271	1.032	1.252	1.290	<0.001	0.867	1.061	0.849	0.885	0.017
count Sex - male	0.971	1.023	0.961	0.981	0.199	0.971	1.038	0.958	0.985	0.432
count Social grade - C1C2	1.002	1.029	0.990	1.014	0.946	1.144	1.045	1.125	1.164	0.002
count Social grade - AB	0.928	1.034	0.914	0.941	0.026	1.422	1.053	1.394	1.451	<0.001
count - Number of adults	0.864	1.012	0.859	0.868	<0.001	0.890	1.021	0.883	0.896	<0.001
count Presence of children - Yes	0.806	1.026	0.796	0.817	<0.001	0.826	1.046	0.812	0.841	<0.001
count Region - North of England	1.042	1.020	1.033	1.052	0.039	0.757	1.035	0.748	0.767	<0.001
count Festival - Valentine's Day	0.998	1.012	0.970	1.027	0.877	0.926	1.020	0.890	0.963	<0.001
count Festival - Easter	1.042	1.013	1.011	1.074	0.001	0.983	1.025	0.944	1.024	0.488
countFestival - Halloween	0.922	1.017	0.884	0.961	<0.001	1.067	1.039	1.008	1.131	0.084
count Festival - Christmas	0.855	1.025	0.819	0.893	<0.001	1.076	1.046	1.013	1.143	0.106
count Interaction Time*Pandemic	0.999	1.001	0.996	1.002	0.328	1.000	1.003	0.996	1.004	0.939
zero Constant	0.078	1.175	0.067	0.092	<0.001	0.473	1.198	0.423	0.528	<0.001
zero Time	0.998	1.001	0.996	1.001	0.146	1.000	1.001	0.999	1.002	0.894
zero Pandemic - during pandemic	1.456	1.087	1.233	1.719	<0.001	0.842	1.072	0.737	0.962	0.013
zero Season - 2	1.060	1.045	0.964	1.165	0.184	0.977	1.033	0.913	1.046	0.478
zero Season - 3	1.411	1.041	1.289	1.545	<0.001	1.040	1.037	0.972	1.113	0.283
zero Season - 4	1.120	1.053	1.005	1.247	0.029	1.182	1.037	1.100	1.271	<0.001
zero Age - 45-54 yrs	0.737	1.098	0.677	0.803	0.001	0.834	1.120	0.780	0.891	0.108
zero Age - 55-64 yrs	0.549	1.122	0.499	0.605	<0.001	0.694	1.139	0.646	0.745	0.005
zero Age - 65+ yrs	0.466	1.123	0.422	0.516	<0.001	0.472	1.141	0.438	0.509	<0.001
zero Sex - male	0.871	1.083	0.812	0.934	0.084	1.335	1.094	1.273	1.401	0.001
zero Social grade - C1C2	0.980	1.106	0.900	1.066	0.839	0.631	1.111	0.597	0.667	<0.001
zero Social grade - AB	1.109	1.121	1.005	1.223	0.367	0.550	1.143	0.512	0.591	<0.001
zero - Number of adults	0.922	1.044	0.889	0.957	0.057	0.689	1.066	0.669	0.710	<0.001
zero Presence of children - Yes	0.866	1.092	0.799	0.939	0.102	0.662	1.116	0.620	0.707	<0.001
zero Region - North of England	0.995	1.069	0.936	1.058	0.941	1.107	1.086	1.057	1.158	0.221
zero Festival - Valentine's Day	0.937	1.114	0.758	1.159	0.547	1.099	1.061	0.959	1.260	0.112
zero Festival - Easter	1.251	1.098	1.043	1.501	0.016	0.964	1.080	0.823	1.129	0.636
zeroFestival - Halloween	1.641	1.122	1.297	2.076	<0.001	0.879	1.099	0.718	1.076	0.173
zero Festival - Christmas	2.542	1.103	2.073	3.118	<0.001	1.327	1.093	1.106	1.591	0.002
zero Interaction Time*Pandemic	0.979	1.010	0.959	0.999	0.029	0.987	1.009	0.970	1.004	0.116
Observations	89,382					89,382				

Outcome	Energy purchased from HFSS					Energy purchased from UPF				
Term	Exp. estimate	SE	95%CI low	95%CI high	p value	Exp. estimate	SE	95%CI low	95%CI high	p value
count Constant	0.519	1.021	0.512	0.526	<0.001	0.602	1.026	0.594	0.610	<0.001
count Time	1.000	1.000	1.000	1.000	0.776	1.000	1.000	1.000	1.000	0.763
count Pandemic - during pandemic		1.006	0.999	1.030	0.022	0.970	1.006	0.955	0.984	<0.001
count Season - 2	1.033	1.003	1.025	1.042	<0.001	1.017	1.003	1.009	1.025	<0.001
count Season - 3	1.039	1.004	1.031	1.048	<0.001	1.024	1.003	1.016	1.032	<0.001
count Season - 4	1.073	1.004	1.064	1.083	<0.001	1.026	1.003	1.017	1.036	<0.001
count Age - 45-54 yrs	0.998	1.013	0.990	1.007	0.903	1.019	1.017	1.011	1.027	0.267
count Age - 55-64 yrs	0.993	1.015	0.985	1.002	0.664	0.995	1.020	0.986	1.003	0.776
count Age - 65+ yrs	0.997	1.016	0.988	1.006	0.864	0.985	1.020	0.977	0.994	0.454
count Sex - male	1.006	1.011	1.000	1.013	0.545	0.998	1.013	0.992	1.004	0.886
count Social grade - C1C2	0.971	1.012	0.964	0.978	0.017	0.955	1.015	0.948	0.962	0.002
count Social grade - AB	0.941	1.015	0.932	0.949	<0.001	0.900	1.018	0.893	0.908	<0.001
count - Number of adults	0.992	1.006	0.989	0.995	0.159	0.992	1.007	0.989	0.995	0.212
count Presence of children - Yes	1.015	1.012	1.007	1.023	0.215	1.055	1.015	1.047	1.063	<0.001
count Region - North of England	1.015	1.009	1.010	1.021	0.095	1.059	1.012	1.054	1.065	<0.001
count Festival - Valentine's Day	1.022	1.007	1.005	1.040	0.001	1.014	1.006	0.998	1.031	0.016
count Festival - Easter	0.982	1.007	0.965	1.000	0.011	0.999	1.006	0.982	1.017	0.915
countFestival - Halloween	0.994	1.010	0.970	1.019	0.573	1.012	1.008	0.987	1.036	0.171
count Festival - Christmas	0.994	1.012	0.969	1.019	0.590	0.991	1.011	0.967	1.017	0.416
count Interaction Time*Pandemic	1.000	1.001	0.998	1.002	0.712	1.000	1.001	0.998	1.002	0.738
zero Constant	0.101	1.239	0.081	0.125	<0.001	0.026	1.318	0.019	0.036	<0.001
zero Time	0.998	1.001	0.995	1.001	0.225	0.997	1.002	0.992	1.001	0.153
zero Pandemic - during pandemic	1.301	1.131	1.030	1.644	0.032	1.306	1.194	0.918	1.860	0.131
zero Season - 2	0.966	1.065	0.850	1.097	0.579	1.014	1.108	0.837	1.230	0.889
zero Season - 3	0.967	1.067	0.848	1.103	0.608	1.164	1.101	0.960	1.412	0.115
zero Season - 4	0.814	1.084	0.697	0.950	0.010	0.893	1.117	0.705	1.129	0.305
zero Age - 45-54 yrs	0.620	1.153	0.544	0.707	0.001	0.651	1.237	0.541	0.785	0.044
zero Age - 55-64 yrs	0.520	1.170	0.454	0.597	<0.001	0.487	1.227	0.399	0.595	<0.001
zero Age - 65+ yrs	0.562	1.167	0.493	0.641	<0.001	0.530	1.226	0.438	0.642	0.002
zero Sex - male	1.228	1.115	1.119	1.347	0.060	1.215	1.156	1.061	1.392	0.180
zero Social grade - C1C2	1.060	1.144	0.935	1.200	0.668	1.300	1.190	1.063	1.589	0.131
zero Social grade - AB	1.338	1.166	1.161	1.541	0.058	1.852	1.217	1.488	2.305	0.002
zero - Number of adults	0.663	1.074	0.625	0.703	<0.001	0.788	1.089	0.727	0.854	0.005
zero Presence of children - Yes	0.455	1.147	0.396	0.522	<0.001	0.345	1.195	0.280	0.426	<0.001
zero Region - North of England	0.690	1.103	0.632	0.754	<0.001	0.600	1.131	0.526	0.684	<0.001
zero Festival - Valentine's Day	0.819	1.155	0.610	1.099	0.165	0.985	1.237	0.647	1.500	0.945
zero Festival - Easter	0.976	1.149	0.734	1.297	0.860	1.131	1.216	0.761	1.682	0.529
zeroFestival - Halloween	0.992	1.250	0.638	1.543	0.971	1.026	1.393	0.535	1.968	0.938
zero Festival - Christmas	2.002	1.197	1.426	2.809	<0.001	3.122	1.247	2.046	4.763	<0.001
zero Interaction Time*Pandemic	0.962	1.016	0.933	0.993	0.016	0.996	1.025	0.954	1.040	0.878
Observations	89,382					89,382				

Outcome	Energy purchased from savoury snacks					Enegy purchased from chocolate & confectionery				
Term	Exp. estimate	SE	95%CI low	95%CI high	p value	Exp. estimate	SE	95%CI low	95%CI high	p value
count Constant	0.138	1.077	0.133	0.144	<0.001	0.150	1.064	0.144	0.156	<0.001
count Time	1.000	1.000	1.000	1.001	0.176	1.001	1.000	1.000	1.001	0.021
count Pandemic - during pandemic	0.932	1.025	0.896	0.970	0.004	0.966	1.025	0.926	1.007	0.162
count Season - 2	1.012	1.013	0.991	1.034	0.335	1.039	1.014	1.015	1.063	0.005
count Season - 3	1.065	1.013	1.042	1.089	<0.001	1.046	1.016	1.022	1.072	0.003
count Season - 4	1.079	1.015	1.053	1.105	<0.001	1.174	1.016	1.144	1.204	<0.001
count Age - 45-54 yrs	0.919	1.044	0.900	0.938	0.050	0.997	1.045	0.974	1.021	0.947
count Age - 55-64 yrs	0.813	1.052	0.795	0.833	<0.001	0.936	1.044	0.913	0.961	0.126
count Age - 65+ yrs	0.734	1.060	0.716	0.752	<0.001	0.870	1.046	0.847	0.893	0.002
count Sex - male	1.132	1.037	1.114	1.151	0.001	1.040	1.032	1.022	1.059	0.215
count Social grade - C1C2	0.906	1.041	0.888	0.923	0.014	0.899	1.038	0.880	0.917	0.004
count Social grade - AB	0.921	1.046	0.900	0.943	0.067	0.975	1.045	0.951	1.000	0.572
count - Number of adults	0.898	1.017	0.891	0.905	<0.001	0.872	1.018	0.864	0.880	<0.001
count Presence of children - Yes	0.804	1.043	0.789	0.820	<0.001	0.876	1.039	0.857	0.895	0.001
count Region - North of England	0.880	1.029	0.867	0.892	<0.001	0.984	1.028	0.969	1.000	0.558
count Festival - Valentine's Day	1.078	1.026	1.031	1.127	0.004	1.043	1.030	0.994	1.094	0.154
count Festival - Easter	0.969	1.026	0.924	1.015	0.207	1.192	1.030	1.137	1.249	<0.001
countFestival - Halloween	1.006	1.041	0.942	1.075	0.879	1.124	1.040	1.051	1.201	0.003
count Festival - Christmas	1.243	1.043	1.160	1.332	<0.001	1.169	1.057	1.081	1.263	0.005
count Interaction Time*Pandemic	1.000	1.003	0.995	1.005	0.994	0.988	1.003	0.983	0.994	<0.001
zero Constant	1.709	1.133	1.591	1.836	<0.001	1.638	1.123	1.525	1.758	<0.001
zero Time	0.999	1.001	0.998	1.000	0.145	0.999	1.001	0.998	1.000	0.068
zero Pandemic - during pandemic	0.966	1.038	0.895	1.044	0.362	0.768	1.041	0.711	0.829	<0.001
zero Season - 2	0.915	1.020	0.878	0.954	<0.001	0.902	1.021	0.865	0.939	<0.001
zero Season - 3	0.967	1.022	0.928	1.008	0.124	0.938	1.022	0.900	0.978	0.003
zero Season - 4	0.871	1.024	0.831	0.912	<0.001	0.713	1.024	0.680	0.746	<0.001
zero Age - 45-54 yrs	0.882	1.086	0.845	0.920	0.127	0.843	1.082	0.809	0.880	0.031
zero Age - 55-64 yrs	0.922	1.094	0.881	0.965	0.367	0.722	1.090	0.690	0.755	<0.001
zero Age - 65+ yrs	1.223	1.096	1.168	1.280	0.029	0.808	1.090	0.771	0.846	0.013
zero Sex - male	1.011	1.067	0.981	1.043	0.861	1.286	1.063	1.248	1.326	<0.001
zero Social grade - C1C2	0.907	1.081	0.874	0.942	0.210	1.057	1.075	1.018	1.097	0.446
zero Social grade - AB	1.037	1.094	0.992	1.084	0.690	1.249	1.089	1.195	1.306	0.009
zero - Number of adults	0.805	1.034	0.791	0.818	<0.001	0.888	1.033	0.874	0.902	<0.001
zero Presence of children - Yes	0.595	1.080	0.572	0.619	<0.001	0.676	1.075	0.650	0.703	<0.001
zero Region - North of England	0.980	1.057	0.953	1.007	0.714	0.757	1.056	0.737	0.779	<0.001
zero Festival - Valentine's Day	0.870	1.041	0.798	0.950	0.001	0.797	1.040	0.731	0.870	<0.001
zero Festival - Easter	1.021	1.043	0.932	1.119	0.617	0.695	1.045	0.632	0.764	<0.001
zeroFestival - Halloween	1.097	1.059	0.966	1.245	0.106	0.949	1.060	0.835	1.078	0.369
zero Festival - Christmas	1.227	1.064	1.078	1.396	0.001	1.699	1.067	1.493	1.933	<0.001
zero Interaction Time*Pandemic	0.990	1.005	0.980	1.000	0.029	1.009	1.005	0.999	1.019	0.055
Observations	89,382					89,382				

Outcome	Energy purchased from low-sugar soft drinks					Energy from medium-sugar soft drinks				
Term	Exp. estimate	SE	95%CI low	95%CI high	p value	Exp. estimate	SE	95%CI low	95%CI high	p value
count Constant	0.021	1.205	0.019	0.022	<0.001	0.038	1.256	0.030	0.048	<0.001
count Time	0.999	1.001	0.998	1.000	0.419	1.004	1.002	1.001	1.007	0.076
count Pandemic - during pandemic	0.871	1.070	0.801	0.948	0.042	0.697	1.219	0.542	0.896	0.068
count Season - 2	1.005	1.046	0.960	1.053	0.908	0.890	1.087	0.778	1.017	0.161
count Season - 3	1.119	1.045	1.068	1.172	0.010	0.952	1.103	0.832	1.089	0.614
count Season - 4	1.000	1.042	0.949	1.054	0.991	0.908	1.126	0.788	1.046	0.415
count Age - 45-54 yrs	0.738	1.116	0.706	0.772	0.006	1.242	1.188	1.086	1.420	0.208
count Age - 55-64 yrs	0.860	1.149	0.819	0.903	0.278	1.185	1.162	1.021	1.376	0.258
count Age - 65+ yrs	0.674	1.140	0.639	0.710	0.003	0.984	1.180	0.848	1.142	0.921
count Sex - male	1.105	1.095	1.067	1.144	0.271	1.077	1.105	0.973	1.192	0.455
count Social grade - C1C2	0.743	1.124	0.713	0.774	0.011	0.656	1.221	0.578	0.744	0.035
count Social grade - AB	0.622	1.136	0.590	0.654	<0.001	0.690	1.219	0.594	0.801	0.061
count - Number of adults	0.909	1.047	0.895	0.923	0.037	0.843	1.062	0.803	0.884	0.004
count Presence of children - Yes	0.725	1.098	0.697	0.754	0.001	1.125	1.179	0.989	1.281	0.474
count Region - North of England	0.875	1.097	0.848	0.904	0.148	0.939	1.101	0.859	1.026	0.510
count Festival - Valentine's Day	1.080	1.079	0.977	1.194	0.309	1.439	1.233	1.085	1.909	0.083
count Festival - Easter	1.106	1.104	1.003	1.221	0.304	0.802	1.132	0.596	1.080	0.075
countFestival - Halloween	0.982	1.138	0.852	1.133	0.891	1.218	1.295	0.790	1.878	0.445
count Festival - Christmas	1.791	1.239	1.529	2.098	0.007	0.640	1.229	0.452	0.906	0.031
count Interaction Time*Pandemic	1.009	1.007	0.999	1.020	0.186	1.038	1.031	1.008	1.069	0.225
zero Constant	3.390	1.175	3.134	3.668	<0.001	50.704	1.325	39.891	64.448	<0.001
zero Time	1.000	1.001	0.999	1.001	0.793	0.996	1.002	0.993	0.999	0.069
zero Pandemic - during pandemic	0.926	1.040	0.851	1.008	0.053	1.427	1.165	1.087	1.872	0.020
zero Season - 2	0.868	1.022	0.830	0.908	<0.001	0.799	1.076	0.694	0.921	0.002
zero Season - 3	0.803	1.023	0.767	0.840	<0.001	0.852	1.086	0.740	0.981	0.051
zero Season - 4	0.946	1.024	0.900	0.995	0.018	0.737	1.082	0.638	0.853	<0.001
zero Age - 45-54 yrs	0.873	1.113	0.834	0.915	0.205	0.839	1.176	0.730	0.964	0.280
zero Age - 55-64 yrs	1.021	1.124	0.972	1.073	0.858	0.849	1.222	0.731	0.987	0.414
zero Age - 65+ yrs	1.292	1.124	1.228	1.359	0.029	0.984	1.219	0.841	1.151	0.936
zero Sex - male	1.183	1.084	1.144	1.223	0.037	1.289	1.152	1.159	1.432	0.073
zero Social grade - C1C2	0.956	1.104	0.918	0.995	0.647	0.823	1.197	0.723	0.936	0.277
zero Social grade - AB	1.343	1.123	1.279	1.411	0.011	0.844	1.213	0.725	0.982	0.380
zero - Number of adults	0.758	1.046	0.744	0.772	<0.001	0.943	1.076	0.896	0.992	0.423
zero Presence of children - Yes	0.654	1.103	0.627	0.682	<0.001	1.194	1.156	1.048	1.360	0.221
zero Region - North of England	0.559	1.074	0.542	0.576	<0.001	1.242	1.143	1.134	1.359	0.106
zero Festival - Valentine's Day	1.035	1.041	0.941	1.139	0.392	0.911	1.158	0.679	1.222	0.523
zero Festival - Easter	0.952	1.044	0.862	1.052	0.259	1.087	1.156	0.794	1.489	0.563
zeroFestival - Halloween	0.996	1.063	0.867	1.144	0.942	1.515	1.238	0.961	2.390	0.051
zero Festival - Christmas	1.216	1.069	1.054	1.401	0.004	0.918	1.203	0.635	1.327	0.643
zero Interaction Time*Pandemic	0.983	1.005	0.973	0.993	<0.001	0.968	1.016	0.938	0.999	0.040
Observations	89,382					89,382				

Outcome	Energy from high-sugar soft drinks					Alcohol volume				
Term	Exp. estimate	SE	95%CI low	95%CI high	p value	Exp. estimate	SE	95%CI low	95%CI high	p value
count Constant	0.102	1.174	0.090	0.116	<0.001	3672.106	1.172	3436.500	3923.867	<0.001
count Time	0.999	1.001	0.997	1.001	0.370	1.001	1.001	1.000	1.002	0.057
count Pandemic - during pandemic	0.810	1.092	0.694	0.944	0.017	1.171	1.041	1.099	1.248	<0.001
count Season - 2	0.926	1.056	0.856	1.003	0.162	1.098	1.022	1.060	1.137	<0.001
count Season - 3	1.145	1.074	1.055	1.243	0.057	1.093	1.027	1.055	1.133	0.001
count Season - 4	0.933	1.068	0.852	1.021	0.291	1.125	1.024	1.083	1.170	<0.001
count Age - 45-54 yrs	0.753	1.114	0.701	0.808	0.008	1.161	1.096	1.116	1.206	0.105
count Age - 55-64 yrs	0.630	1.141	0.583	0.680	<0.001	1.060	1.110	1.017	1.104	0.579
count Age - 65+ yrs	0.667	1.198	0.608	0.732	0.025	0.919	1.130	0.881	0.958	0.490
count Sex - male	1.151	1.104	1.082	1.224	0.156	1.112	1.075	1.084	1.141	0.143
count Social grade - C1C2	0.830	1.115	0.777	0.887	0.088	0.792	1.117	0.766	0.818	0.034
count Social grade - AB	0.796	1.116	0.731	0.866	0.037	0.723	1.121	0.696	0.751	0.005
count - Number of adults	0.854	1.055	0.832	0.876	0.003	0.695	1.038	0.686	0.704	<0.001
count Presence of children - Yes	0.690	1.091	0.645	0.738	<0.001	0.885	1.091	0.853	0.917	0.160
count Region - North of England	0.852	1.097	0.806	0.900	0.082	1.375	1.070	1.342	1.409	<0.001
count Festival - Valentine's Day	0.840	1.088	0.706	0.998	0.038	0.911	1.038	0.847	0.981	0.012
count Festival - Easter	0.946	1.093	0.814	1.100	0.536	1.084	1.040	1.008	1.166	0.040
countFestival - Halloween	0.833	1.119	0.653	1.064	0.105	0.911	1.058	0.821	1.011	0.098
count Festival - Christmas	0.836	1.124	0.673	1.039	0.126	1.037	1.064	0.934	1.151	0.561
count Interaction Time*Pandemic	1.014	1.011	0.996	1.033	0.217	0.995	1.005	0.987	1.002	0.265
zero Constant	10.398	1.333	8.987	12.030	<0.001	8.426	1.189	7.778	9.129	<0.001
zero Time	1.001	1.001	0.999	1.003	0.304	0.999	1.001	0.998	1.000	0.320
zero Pandemic - during pandemic	1.001	1.086	0.846	1.186	0.986	0.820	1.041	0.756	0.890	<0.001
zero Season - 2	0.844	1.048	0.773	0.922	<0.001	0.829	1.022	0.793	0.867	<0.001
zero Season - 3	0.891	1.048	0.813	0.976	0.014	0.816	1.024	0.779	0.854	<0.001
zero Season - 4	0.843	1.054	0.763	0.933	0.001	0.686	1.025	0.653	0.721	<0.001
zero Age - 45-54 yrs	1.691	1.201	1.558	1.835	0.004	0.764	1.125	0.730	0.801	0.022
zero Age - 55-64 yrs	1.792	1.249	1.641	1.957	0.009	0.675	1.135	0.643	0.709	0.002
zero Age - 65+ yrs	3.133	1.268	2.824	3.477	<0.001	0.725	1.138	0.689	0.762	0.013
zero Sex - male	0.888	1.157	0.832	0.949	0.416	1.011	1.092	0.978	1.044	0.903
zero Social grade - C1C2	1.562	1.185	1.453	1.680	0.008	0.782	1.119	0.751	0.815	0.029
zero Social grade - AB	1.992	1.213	1.813	2.189	<0.001	0.801	1.139	0.763	0.840	0.087
zero - Number of adults	0.810	1.078	0.785	0.835	0.005	0.843	1.048	0.828	0.857	<0.001
zero Presence of children - Yes	1.238	1.197	1.147	1.338	0.234	1.337	1.115	1.281	1.396	0.007
zero Region - North of England	1.364	1.141	1.286	1.447	0.018	0.612	1.083	0.594	0.631	<0.001
zero Festival - Valentine's Day	0.963	1.087	0.794	1.168	0.649	0.820	1.039	0.747	0.901	<0.001
zero Festival - Easter	0.715	1.078	0.604	0.848	<0.001	0.976	1.039	0.887	1.073	0.515
zeroFestival - Halloween	1.052	1.125	0.801	1.382	0.667	1.198	1.056	1.048	1.370	0.001
zero Festival - Christmas	0.738	1.126	0.578	0.943	0.011	1.198	1.068	1.048	1.371	0.006
zero Interaction Time*Pandemic	0.988	1.010	0.968	1.008	0.225	0.986	1.004	0.976	0.996	0.001
Observations	89,382					89,382				

Outcome	OOH purchasing				
Term	Exp. estimate	SE	95%CI low	95%CI high	p value
count Constant	1.373	1.351	1.248	1.510	0.292
count Time	0.999	1.001	0.997	1.000	0.037
count Pandemic - during pandemic	0.502	1.081	0.449	0.561	<0.001
count Season - 2	0.962	1.025	0.913	1.014	0.122
count Season - 3	0.995	1.024	0.945	1.047	0.822
count Season - 4	0.995	1.024	0.940	1.053	0.832
count Age - 45-54 yrs	0.913	1.181	0.871	0.956	0.581
count Age - 55-64 yrs	1.046	1.191	0.993	1.102	0.797
count Age - 65+ yrs	0.718	1.261	0.668	0.772	0.153
count Sex - male	1.503	1.137	1.446	1.563	0.002
count Social grade - C1C2	1.024	1.218	0.971	1.079	0.905
count Social grade - AB	1.031	1.300	0.962	1.104	0.908
count - Number of adults	1.005	1.078	0.983	1.027	0.948
count Presence of children - Yes	0.995	1.173	0.950	1.043	0.976
count Region - North of England	1.109	1.125	1.070	1.149	0.381
count Festival - Valentine's Day	1.006	1.027	0.906	1.117	0.826
count Festival - Easter	0.874	1.042	0.772	0.990	0.001
countFestival - Halloween	1.012	1.041	0.871	1.174	0.771
count Festival - Christmas	0.727	1.060	0.616	0.859	<0.001
count Interaction Time*Pandemic	1.018	1.008	1.004	1.033	0.025
zero Constant	0.072	4.029	0.031	0.168	0.059
zero Time	1.008	1.013	0.993	1.023	0.537
zero Pandemic - during pandemic	2.587	2.051	0.949	7.052	0.186
zero Season - 2	2.538	1.653	1.447	4.454	0.064
zero Season - 3	1.046	1.439	0.571	1.915	0.902
zero Season - 4	1.295	1.478	0.699	2.399	0.508
zero Age - 45-54 yrs	-				
zero Age - 55-64 yrs	-				
zero Age - 65+ yrs	-				
zero Sex - male	8.434	3.598	4.996	14.238	0.096
zero Social grade - C1C2	<0.001	8.971	<0.001	37201.403	<0.001
zero Social grade - AB	5.639	4.450	3.211	9.903	0.247
zero - Number of adults	0.362	2.156	0.281	0.465	0.186
zero Presence of children - Yes	-				
zero Region - North of England	-				
zero Festival - Valentine's Day	-				
zero Festival - Easter	-				
zeroFestival - Halloween	-				
zero Festival - Christmas	-				
zero Interaction Time*Pandemic	0.799	1.107	0.689	0.927	0.027
Observations	16,806				

count Interaction Time*Pandemic	1.009	1.011	0.994	1.024	0.380
count Interaction Pandemic*PPP - 2	1.037	1.190	0.872	1.233	0.835
count Interaction Pandemic*PPP - 3	-				
count Interaction Pandemic*PPP - 4	-				
zero Constant	<0.001	3.347	<0.001	Inf	<0.001
zero Time	1.093	1.066	1.026	1.164	0.164
zero Pandemic - during pandemic	4012206.126	12.898	<0.001	Inf	<0.001
zero Season - 2	3.909	1.453	2.071	7.376	<0.001
zero Season - 3	20.944	9.676	2.927	149.891	0.180
zero Season - 4	8.927	5.784	2.149	37.085	0.212
zero Age - 45-54 yrs	-				
zero Age - 55-64 yrs	-				
zero Age - 65+ yrs	-				
zero Sex - male	1.013	1.659	0.734	1.397	0.980
zero Social grade - C1C2	0.791	1.675	0.528	1.184	0.649
zero Social grade - AB	1.178	1.905	0.726	1.912	0.799
zero - Number of adults	1.082	1.377	0.887	1.320	0.805
zero Presence of children - Yes	-				
zero Region - North of England	-				
zero Festival - Valentine's Day	-				
zero Festival - Easter	-				
zeroFestival - Halloween	-				
zero Festival - Christmas	-				
zero PPP - 2	3595603.272	2.499	<0.001	Inf	<0.001
zero PPP - 3	-				
zero PPP - 4	-				
zero Interaction Time*Pandemic	0.779	1.101	0.705	0.860	0.009
zero Interaction Pandemic*PPP - 2	<0.001	12.575	<0.001	Inf	<0.001
zero Interaction Pandemic*PPP - 3	-				
zero Interaction Pandemic*PPP - 4	-				
Observations	16,806				
