# Supplementary material for: Changes in food and drink purchasing behaviour in England during the first 3 months of the COVID-19 pandemic: an interrupted time series analysis
Source: Public Health Nutr. 2024 Nov 22;27(1):e252. doi: 10.1017/S1368980024001071 (PMC11705008; doi:10.1017/S1368980024001071)
Supplement: Kalbus et al. supplementary material 4 — Kalbus et al. supplementary material [file S1368980024001071sup004.pdf]

Supplementary Material 3: Subgroup analysis results

It is important to note that the following results should be seen as hypothesis generating rather than testing. Tables below present marginal mean differences where interactions have been detected ($p<0.05$). Below the tables, coefficients from the underlying models are presented.

Region

Region moderated the association between pandemic restrictions and purchased take-home energy from fruit & vegetables, with a decrease observed in the North of England (-10.1%, 95% CI -14.7 to -5.5), but not in London.

Presence of children

Having one or more children moderated the association between pandemic restrictions and total energy purchased, energy from HFSS products, UPF, savoury snacks, medium-sugar soft drinks, as well as alcohol volume. Total energy purchased increased more in households with children (22.3%, 95% CI 18.1 to 26.6 vs 15.9%, 95% CI 12.8 to 18.9). Households with children purchased more energy from HFSS products (3.0%, 95% CI 1.4 to 4.7), and decreased energy from savoury snacks (-5.7%, 95% CI -11.4 to -0.1) and medium-sugar soft drinks (-39.9%, 95% CI -76.9 to -2.9), while there was no change observed for households without children. Reductions in energy from UPF were greater in households without children (-3.8%, 95% CI -5.0 to -2.6 vs -1.8%, 95% CI -3.3 to -0.2). While both households with and without children increased purchased volume of alcoholic beverages, the increase was greater for those with children (64.7%, 95% CI 38.2 to 89.2 vs 28.9%, 16.3 to 41.5).

Age

Age of the main reporter moderated the association between pandemic restrictions and most purchase outcomes. Main shoppers aged 65 years and older were associated with the smallest increase in total energy purchased during pandemic restrictions compared to other age groups (4.7%, 95% CI 0.3 to 9.0). Only among this age group, a decrease in energy purchased from chocolate & confectionery (-9.7%, 95% CI -18.5 to -1.0), and no change in volume of alcoholic beverages was observed. Households with main shoppers aged 45-54 years saw the largest increase in purchased alcohol volume of 63.2% (95% CI 38.2 to 88.2). Main shoppers aged between 45 and 64 years purchased less energy from fruit & vegetables (45-54 years -9.4%, 95% CI -15.2 to -3.5; 55-64 years -6.6%, 95% CI -12.5 to -0.8), while there was no change in

the youngest and oldest age groups. Energy purchased from HFSS products increased only among main shoppers under the age of 55 years (< 45 years 2.7%, 95% CI 0.7 to 4.6; 45-54 years 3.4%, 95% CI 1.5 to 5.4). Furthermore, 55- to 64-year-old main shoppers were the only group linked to a reduction of energy purchased from savoury snacks (-8.6%, 95% CI -16.6 to -0.5). Despite effect modification by age, purchased energy from low-sugar soft drinks did not change during pandemic restrictions in any age group. Energy from high-sugar soft drinks decreased in all but the oldest age group, potentially due to the already low consumption levels of this group, with the highest decrease observed in the youngest age group (-13.5%, 95% CI -43.4 to -16.5). OOH purchasing fell in all age groups, but most in the oldest group (-67.1%, 95% CI -106.4 to -27.9).

Social grade

Social grade of the main shopper moderated the relationship between pandemic restrictions and total energy purchased, energy purchased from fruit & vegetables, UPF, savoury snacks, chocolate & confectionery, and low-sugar soft drinks, alcohol volume, and OOH purchasing. The highest increase in total energy purchased was observed among households in social grade AB (22.4%, 95% CI 16.9 to 27.9). Social grade C1C2 was the only group associated with a reduction in energy purchased from fruit & vegetables (-10.1%, 95% CI -14.6 to -5.7). Energy purchased from UPF fell in all social grades, but those in the AB grades had the largest reduction (-5.0%, 95% CI 7.0 to -3.1). While main shoppers in group AB reported the greatest increase in purchased alcoholic beverages (39.1%, 95% CI 17.9 to 60.2) during pandemic restrictions, there was no change for group DE. Similarly, no change in OOH purchasing was observed among group DE. Despite effect modification by social grade, purchased energy from savoury snacks, chocolate & confectionery as well as low-sugar soft drinks did not change during pandemic restrictions in any group.

Usual purchasing

Usual purchasing levels moderated the relationship between pandemic restrictions and all purchasing outcomes, with varying directions of the relationship. For most outcomes we observed that higher usual purchasing levels were linked to greater reductions during pandemic restrictions, and lower usual purchasing was associated with greater increases during pandemic restrictions. While total energy purchased, for example, increased in the overall sample, households in the lowest quartile of usual purchasing had the largest increase of 41.2% (95% CI 35.8 to 46.5), while those in the highest quartile did not change the amount of energy

purchased during pandemic restrictions. Energy from fruit & vegetables, which decreased in the overall sample, increased for those in the lowest quartile of usual purchasing (23.5%, 95% CI 14.4 to 32.6), but decreased for those in the upper two quartiles (second-highest quartile: -9.2%, 95% CI -14.2 to -4.2; highest quartile: -14.2%, 95% CI -19.2 to -9.2). While the relative increase in purchasing of alcoholic beverages also followed this pattern, the absolute increases did not. Higher usual purchasing of alcoholic beverages was linked to a greater absolute increase during pandemic restrictions (lowest tertile 123.2 ml, 95% CI 71.3 to 175.0; highest tertile 708.3 ml, 95% CI 381.3 to 1035.3).

Table S1. Marginal mean differences in purchase outcomes by region

Outcome		London	North of England
Total energy purchased	kcal (95% CI)	–	
	% (95% CI)		
Energy purchased from fruit & vegetables	kcal (95% CI)	-58.69 (-138.34, 20.96)	-120.22 (-175.19, -65.25)
	% (95% CI)	-3.74 (-8.80, 1.33)	-10.08 (-14.69, -5.47)
Energy purchased from HFSS products	kcal (95% CI)	–	
	% (95% CI)		
Energy purchased from UPF	kcal (95% CI)	–	
	% (95% CI)		
Energy purchased from savoury snacks	kcal (95% CI)	–	
	% (95% CI)		
Energy purchased from chocolate & confectionery	kcal (95% CI)	–	
	% (95% CI)		
Energy purchased from low-sugar soft drinks	kcal (95% CI)	–	
	% (95% CI)		
Energy purchased from medium-sugar soft drinks	kcal (95% CI)	–	
	% (95% CI)		
Energy purchased from high-sugar soft drinks	kcal (95% CI)	–	
	% (95% CI)		
Volume of alcoholic beverages purchased	ml (95% CI)	–	
	% (95% CI)		
OOH purchasing	days (95% CI)	–	
	% (95% CI)		

95% CI = 95% confidence interval; HFSS = high in fat, salt and sugar; OOH = out-of-home; UPF = ultra-processed foods. Results shown from models were interaction terms with pandemic restrictions in either count or zero component had $p<0.05$. Models were adjusted for season, festivals, age, sex, and occupational social grade of the main shopper, number of adults, and presence of children.

Table S2. Marginal mean differences in purchase outcomes by presence of children

Outcome		Children	No children
Total energy purchased	kcal (95% CI)	6330.17 (5130.97, 7529.37)	5602.85 (4522.00, 6683.71)
	% (95% CI)	22.32 (18.09, 26.55)	15.85 (12.80, 18.91)
Energy purchased from fruit & vegetables	kcal (95% CI)	–	
	% (95% CI)		
Energy purchased from HFSS products	kcal (95% CI)	418.18 (192.01, 644.34)	85.77 (-98.54, 270.08)
	% (95% CI)	3.04 (1.39, 4.68)	0.64 (-0.73, 2.00)
Energy purchased from UPF	kcal (95% CI)	-300.23 (-566.75, -33.71)	-618.65 (-818.81, -418.49)
	% (95% CI)	-1.75 (-3.31, -0.20)	-3.81 (-5.04, -2.58)
Energy purchased from savoury snacks	kcal (95% CI)	35.83 (-30.96, 102.63)	3.50 (-3.03, 10.03)
	% (95% CI)	-60.28 (-119.51, -1.04)	-5.74 (-11.39, -0.10)
Energy purchased from chocolate & confectionery	kcal (95% CI)	–	
	% (95% CI)		
Energy purchased from low-sugar soft drinks	kcal (95% CI)	–	
	% (95% CI)		
Energy purchased from medium-sugar soft drinks	kcal (95% CI)	5.61 (-4.76, 15.98)	-5.86 (-11.30, -0.42)
	% (95% CI)	49.42 (-41.90, 140.75)	-39.86 (-76.86, -2.87)
Energy purchased from high-sugar soft drinks	kcal (95% CI)	–	
	% (95% CI)		
Volume of alcoholic beverages purchased	ml (95% CI)	635.90 (381.03, 890.77)	418.33 (236.06, 600.59)
	% (95% CI)	63.67 (38.15, 89.19)	28.93 (16.33, 41.54)
OOH purchasing	days (95% CI)	–	
	% (95% CI)		

95% CI = 95% confidence interval; HFSS = high in fat, salt and sugar; OOH = out-of-home; UPF = ultra-processed foods. Results shown from models were interaction terms with pandemic restrictions in either count or zero component had p<0.05. Models were adjusted for season, region, festivals, age, sex, and occupational social grade of the main shopper, and number of adults.

Table S3. Marginal mean differences in purchase outcomes by age of the main shopper

Outcome		< 45 years	45–54 years	55–64 years	65+ years
Total energy purchased	kcal (95% CI)	6700.39 (5385.97, 8014.82)	7579.26 (6189.58, 8968.93)	5684.45 (4216.67, 7152.22)	1704.19 (112.49, 3295.89)
	% (95% CI)	24.81 (19.95, 29.68)	16.09 (19.62, 28.44)	16.09 (11.93, 20.24)	4.66 (0.31, 9.01)
Energy purchased from fruit & vegetables	kcal (95% CI)	-85.49 (-175.51, 4.53)	-115.73 (-187.71, -43.75)	-78.68 (-148.27, -9.10)	-67.46 (-137.77, 2.85)
	% (95% CI)	-6.47 (-13.29, 0.34)	-9.36 (-15.18, -3.54)	-6.64 (-12.51, -0.77)	-5.50 (-11.24, 0.23)
Energy purchased from HFSS products	kcal (95% CI)	356.26 (95.74, 616.77	458.75 (197.13, 720.36)	104.75 (-147.52, 357.02)	-232.38 (-490.46, 25.70)
	% (95% CI)	2.67 (0.72, 4.62)	3.41 (1.46, 5.35)	0.78 (-1.09, 2.64	-1.71 (-3.61, 0.19)
Energy purchased from UPF	kcal (95% CI)	–			
	% (95% CI)				
Energy purchased from savoury snacks	kcal (95% CI)	28.43 (-66.90, 123.77	4.82 (-80.33, 89.97)	-90.15 (-174.71, -5.60	-21.66 (-88.43, 45.10)
	% (95% CI)	2.32 (-5.47, 10.11)	0.40 (-6.71, 7.51)	-8.55 (-16.56, -0.53)	-2.65 (-10.82, 5.52)
Energy purchased from chocolate & confectionery	kcal (95% CI)	42.56 (-73.71, 158.84)	67.47 (-47.38, 182.33)	-26.02 (-142.09, 90.05)	-129.73 (-246.60, -12.86)
	% (95% CI)	3.14 (-5.44, 11.72)	4.62 (-3.24, 12.48	-1.75 (-9.57, 6.06)	-9.72 (-18.48, -0.96)
Energy purchased from low-sugar soft drinks	kcal (95% CI)	-2.70 (-30.16, 24.77)	12.03 (-11.18, 35.24)	-16.03 (-45.36, 13.31)	7.99 (-14.23, 30.21)
	% (95% CI)	1.82 (-20.32, 16.68)	10.52 (-9.78, 30.82)	-12.43 (-35.19, 10.32)	9.33 (-16.63, 35.29)
Energy purchased from medium-sugar soft drinks	kcal (95% CI)	–			
	% (95% CI)				
Energy purchased from high-sugar soft drinks	kcal (95% CI)	–			
	% (95% CI)				
Volume of alcoholic beverages purchased	ml (95% CI)	565.93 (357.03, 774.84)	878.11 (530.92, 1225.30)	368.54 (160.64, 576.45)	118.60 (-42.38, 279.59)

	% (95% CI)	56.49 (35.64, 77.34	63.20 (38.21, 88.19)	25.34 (11.05, 39.64)	9.61 (-3.43, 22.66)
OOH purchasing	days (95% CI)	-0.61 (-0.90, -0.33)	-0.54 (-0.78, -0.30)	-0.80 (-1.11, -0.48)	-0.79 (-1.25, -0.33)
	% (95% CI)	-39.67 (-58.24, -21.09)	-38.35 (-55.23, -21.48)	-48.26 (-67.39, -29.14)	-67.12 (-106.35, -27.88)

95% CI = 95% confidence interval; HFSS = high in fat, salt and sugar; OOH = out-of-home; UPF = ultra-processed foods. Results shown from models were interaction terms with pandemic restrictions in either count or zero component had p<0.05. Models were adjusted for season, region, festivals, sex, and occupational social grade of the main shopper, number of adults, and presence of children.

Table S4. Marginal mean differences in purchase outcomes by occupational social grade of the main shopper

Outcome		DE (lowest)	C1C2	AB (highest)
Total energy purchased	kcal (95% CI)	2478.74 (934.64, 4022.84)	6563.27 (5452.24, 7674.30)	7217.35 (5449.77, 8984.94)
	% (95% CI)	6.94 (2.62, 11.27)	18.65 (15.49, 21.80)	22.37 (16.89, 27.85)
Energy purchased from fruit & vegetables	kcal (95% CI)	-26.65 -104.32, 51.01)	-120.64 (-173.77, -67.52)	-34.12 (-127.42, 59.19)
	% (95% CI)	-2.73 (-10.69, 5.23)	-10.11(-14.56, -5.66)	-2.31 (-8.62, 4.00)
Energy purchased from HFSS products	kcal (95% CI)	–		
	% (95% CI)			
Energy purchased from UPF	kcal (95% CI)	-451.40 (-807.77, -95.03)	-476.07 (-676.09, -276.06)	-769.99 (-1071.18, -468.80)
	% (95% CI)	-2.66 (-4.75, -0.56)	-2.94 (-4.17, -1.70)	-5.03 (-7.00, -3.06)
Energy purchased from savoury snacks	kcal (95% CI)	-94.16 (-194.98, 6.66)	-18.38 (-79.13, 42.37	-0.66 (-80.86, 79.55)
	% (95% CI)	-8.47 (-17.54, 0.60	-1.76 (-7.60, 4.07)	-0.07 (-8.16, 8.03)
Energy purchased from chocolate & confectionery	kcal (95% CI)	-44.60 (-214.24, 125.04)	-12.60 (-102.61, 77.40)	-65.41 (-183.98, 53.17)
	% (95% CI)	-2.63 (-12.65, 7.39)	-0.85 (-6.92, 5.22)	-4.34 (-12.21, 3.53)
Energy purchased from low-sugar soft drinks	kcal (95% CI)	12.37 (-34.97, 59.71)	-1.95 (-23.65, 19.75)	3.94 (-13.70, 21.58)
	% (95% CI)	7.57 (-21.39, 36.53)	-1.54 (-18.72, 15.64)	4.53 (-15.76, 24.83)
Energy purchased from medium-sugar soft drinks	kcal (95% CI)	–		
	% (95% CI)			
Energy purchased from high-sugar soft drinks	kcal (95% CI)	–		
	% (95% CI)			
Volume of alcoholic beverages purchased	ml (95% CI)	160.93 (-78.79, 400.65)	583.50 (374.97, 792.02)	501.04 (230.05, 772.04)
	% (95% CI)	10.09 (-4.94, 25.11)	41.04 (26.38, 55.71)	39.07 (17.94, 60.21)
OOH purchasing	days (95% CI)	-0.36 (-0.76, 0.03)	-0.63 (-0.85, -0.42)	-0.56 (-0.92, -0.21)
	% (95% CI)	-27.07 (-56.41, 2.26)	-44.55 (-59.67, -29.42)	-41.99 (-68.20, -15.79)

95% CI = 95% confidence interval; HFSS = high in fat, salt and sugar; OOH = out-of-home; UPF = ultra-processed foods. Results shown from models were interaction terms with pandemic restrictions in either count or zero component had p<0.05. Models were adjusted for season, region, festivals, age and sex of the main shopper, number of adults, and presence of children.

Table S5. Marginal mean differences in purchase outcomes by usual purchasing (in quartiles/tertiles/dichotomised)

Outcome		1 (lowest)	2	3	4 (highest)
Total energy purchased	kcal (95% CI)	7408.10 (6440.51, 8375.69)	6130.26 (5009.32, 7251.20)	5053.41 (3829.31, 6277.52)	1938.95 (-66.50, 3944.40)
	% (95% CI)	41.15 (35.77, 46.52)	23.53 (19.23, 27.83)	15.32 (11.61, 19.04)	4.07 (-0.14, 8.29)
Energy purchased from fruit & vegetables	kcal (95% CI)	139.90 (85.77, 194.04)	17.96 (-47.46, 83.38)	-123.06 (-189.70, -56.43)	-328.30 (-444.59, -212.01)
	% (95% CI)	23.50 (14.40, 32.59)	1.90 (-5.03, 8.84)	-9.24 (-14.24, -4.24)	-14. 17 (-19.20, -9.15)
Energy purchased from HFSS products	kcal (95% CI)	691.78 (428.58, 954.97)	475.10 (236.83, 713.37)	343.09 (111.34, 574.83)	-724.25 (-985.73, -462.77)
	% (95% CI)	6.33 (3.92, 8.74)	3.75 (1.87, 5.63)	2.45 (0.79, 4.10)	-4.58 (-6.23, -2.93
Energy purchased from UPF	kcal (95% CI)	57.62 (-212.36, 327.61)	-312.18 (-576.07, -48.28)	-620.99 (-897.04, -344.94)	-1041.20 (-1334.91, -747.50)
	% (95% CI)	0.48 (-1.76, 2.71)	-2.07 (-3.82, -0.32)	-3.65 (-5.27, -2.03)	-5.30 (-6.80, -3.81)
Energy purchased from savoury snacks	kcal (95% CI)	128.88 (63.90, 193.86)	41.37 (-22.12, 104.86)	-5.12 (-85.28, 75.03)	-223.46 (-345.79, -101.14)
	% (95% CI)	30.31 (15.03, 45.60)	5.49 (-2.93, 13.91)	-0.42 (-7.03, 6.18)	-10.77 (-16.66, -4.87)
Energy purchased from chocolate & confectionery	kcal (95% CI)	193.54 (121.26, 265.82)	100.74 (7.73, 193.76)	-5.00 (-117.47, 107.48)	-294.82 (-451.63, -138.00)
	% (95% CI)	35.91 (22.50, 49.32)	10.24 (0.79, 19.69)	-0.34 (-7.94, 7.26)	-11.82 (-18.10, -5.53)
Energy purchased from low-sugar soft drinks	kcal (95% CI)	19.31 (-1.22, 39.84)	21.44 (8.68, 34.20)	23.85 (6.14, 41.56)	-67.39 (-115.56, -19.22)
	% (95% CI)	69.01 (-4.39, 142.41)	46.47 (18.81, 74.13)	27.19 (7.00, 47.39)	-21.66 (-37.14, -6.17)
		1 (low)	2 (high)		
Energy purchased from medium-sugar soft drinks	kcal (95% CI)	0.41 (-3.02, 3.83)	-21.47 (-40.76, -2.18)		
	% (95% CI)	6.11 (-45.12, 57.34)	-44.38 (-84.24, -4.51)		

Energy purchased from high-sugar soft drinks	kcal (95% CI)	6.31 (0.47, 12.1)	-27.0 (-55.5, 1.49)	
	% (95% CI)	80.91 (6.08, 155.74)	-20.41 (-41.95, 1.12)	
OOH purchasing	days (95% CI)	-0.17 (-0.27, -0.07)	-0.68 (-0.97, -0.38)	
	% (95% CI)	-32.92 (-52.39, -13.45)	-32.71 (-46.81, -18.61)	
		1 (lowest)	2	3 (highest)
Volume of alcoholic beverages purchased	ml (95% CI)	123.15 (71.34, 174.95)	354.24 (241.84, 466.64)	708.28 (381.26, 1035.30)
	% (95% CI)	153.16 (88.73, 217.60)	86.29 (58.91, 113.67)	25.90 (13.94, 37.85)

95% CI = 95% confidence interval; HFSS = high in fat, salt and sugar; OOH = out-of-home; UPF = ultra-processed foods. Results shown from models were interaction terms with pandemic restrictions in either count or zero component had $p<0.05$. Models were adjusted for season, region, festivals, age, sex, and occupational social grade of the main shopper, number of adults, and presence of children.

Model coefficients from secondary analysis; interactions with region

HFSS = high in fat, salt and sugar; UPF = ultra-processed food; OOH = out-of-home

Due to multicollineraity, the variables region, presence of children, and age of the main shopper were not included in the OOH models

Outcome	Total Energy purchased					Energy purchased from fruit & vegetables				
Term	Exp. estimate	SE	95%CI low	95%CI high	p value	Exp. estimate	SE	95%CI low	95%CI high	p value
count Constant	15111.616	1.047	14757.438	15474.295	<0.001	0.082	1.089	0.079	0.085	<0.001
count Time	1.001	1.000	1.000	1.001	<0.001	0.999	1.000	0.998	0.999	<0.001
count Pandemic - during pandemic	1.203	1.017	1.168	1.238	<0.001	0.939	1.027	0.903	0.976	0.018
count Season - 2	1.007	1.006	0.993	1.021	0.208	1.029	1.012	1.010	1.048	0.023
count Season - 3	0.970	1.007	0.956	0.983	<0.001	1.025	1.013	1.006	1.045	0.052
count Season - 4	1.071	1.007	1.054	1.087	<0.001	0.894	1.013	0.876	0.913	<0.001
count Age - 45-54 yrs	1.147	1.029	1.131	1.163	<0.001	0.911	1.051	0.894	0.929	0.061
count Age - 55-64 yrs	1.256	1.032	1.237	1.275	<0.001	0.862	1.060	0.844	0.880	0.011
count Age - 65+ yrs	1.271	1.032	1.252	1.291	<0.001	0.867	1.061	0.849	0.886	0.017
count Sex - male	0.971	1.023	0.961	0.981	0.199	0.971	1.038	0.958	0.985	0.432
count Social grade - C1C2	1.002	1.029	0.990	1.014	0.946	1.144	1.045	1.125	1.164	0.002
count Social grade - AB	0.928	1.034	0.914	0.941	0.026	1.422	1.053	1.393	1.451	<0.001
count - Number of adults	0.864	1.012	0.859	0.868	<0.001	0.890	1.021	0.883	0.896	<0.001
count Presence of children - Yes	0.806	1.026	0.796	0.817	<0.001	0.826	1.046	0.812	0.841	<0.001
count Region - North of England	1.045	1.021	1.034	1.055	0.037	0.765	1.036	0.755	0.776	<0.001
count Festival - Valentine's Day	0.998	1.012	0.970	1.027	0.877	0.926	1.020	0.890	0.963	<0.001
count Festival - Easter	1.042	1.013	1.011	1.074	0.001	0.983	1.025	0.943	1.024	0.471
countFestival - Halloween	0.922	1.017	0.884	0.961	<0.001	1.067	1.039	1.008	1.131	0.084
count Festival - Christmas	0.855	1.025	0.819	0.893	<0.001	1.077	1.046	1.014	1.144	0.104
count Interaction Time*Pandemic	0.999	1.001	0.996	1.002	0.330	1.000	1.003	0.996	1.004	0.994
count Interaction Pandemic*Region	0.987	1.019	0.963	1.011	0.480	0.942	1.027	0.911	0.973	0.024
zero Constant	0.079	1.175	0.068	0.093	<0.001	0.478	1.199	0.427	0.534	<0.001
zero Time	0.998	1.001	0.996	1.001	0.146	1.000	1.001	0.999	1.002	0.895
zero Pandemic - during pandemic	1.336	1.097	1.104	1.616	0.002	0.779	1.091	0.667	0.910	0.004
zero Season - 2	1.060	1.045	0.964	1.165	0.185	0.977	1.033	0.913	1.046	0.478
zero Season - 3	1.411	1.041	1.289	1.545	<0.001	1.040	1.037	0.972	1.113	0.283
zero Season - 4	1.120	1.053	1.005	1.247	0.029	1.182	1.037	1.100	1.271	<0.001
zero Age - 45-54 yrs	0.737	1.098	0.677	0.802	0.001	0.833	1.120	0.779	0.891	0.107
zero Age - 55-64 yrs	0.549	1.122	0.499	0.605	<0.001	0.694	1.139	0.646	0.744	0.005
zero Age - 65+ yrs	0.466	1.123	0.422	0.516	<0.001	0.472	1.141	0.438	0.509	<0.001
zero Sex - male	0.871	1.083	0.812	0.934	0.085	1.335	1.094	1.273	1.401	0.001
zero Social grade - C1C2	0.979	1.106	0.900	1.066	0.836	0.631	1.111	0.597	0.667	<0.001
zero Social grade - AB	1.108	1.121	1.005	1.222	0.369	0.550	1.143	0.512	0.591	<0.001
zero - Number of adults	0.922	1.044	0.889	0.956	0.057	0.689	1.066	0.669	0.710	<0.001
zero Presence of children - Yes	0.866	1.092	0.799	0.939	0.101	0.662	1.116	0.620	0.707	<0.001
zero Region - North of England	0.968	1.072	0.905	1.036	0.642	1.087	1.088	1.035	1.142	0.324
zero Festival - Valentine's Day	0.937	1.114	0.758	1.160	0.547	1.099	1.061	0.959	1.260	0.112
zero Festival - Easter	1.252	1.098	1.043	1.501	0.016	0.965	1.080	0.824	1.130	0.638
zeroFestival - Halloween	1.641	1.122	1.298	2.077	<0.001	0.879	1.099	0.718	1.076	0.173
zero Festival - Christmas	2.543	1.103	2.073	3.119	<0.001	1.327	1.093	1.106	1.591	0.002
zero Interaction Time*Pandemic	0.979	1.010	0.959	0.999	0.029	0.987	1.009	0.970	1.004	0.115
zero Interaction Pandemic*Region	1.161	1.091	0.991	1.360	0.086	1.142	1.089	0.999	1.304	0.121
Observations	89,382					89,382				

Outcome	Energy purchased from HFSS					Energy purchased from UPF				
Term	Exp. estimate	SE	95%CI low	95%CI high	p value	Exp. estimate	SE	95%CI low	95%CI high	p value
count Constant	0.520	1.021	0.512	0.527	<0.001	0.603	1.026	0.594	0.611	<0.001
count Time	1.000	1.000	1.000	1.000	0.775	1.000	1.000	1.000	1.000	0.763
count Pandemic - during pandemic	1.006	1.008	0.989	1.024	0.424	0.964	1.008	0.948	0.980	<0.001
count Season - 2	1.033	1.003	1.025	1.042	<0.001	1.017	1.003	1.009	1.025	<0.001
count Season - 3	1.039	1.004	1.031	1.048	<0.001	1.024	1.003	1.016	1.032	<0.001
count Season - 4	1.073	1.004	1.064	1.083	<0.001	1.026	1.003	1.017	1.036	<0.001
count Age - 45-54 yrs	0.998	1.013	0.990	1.007	0.901	1.019	1.017	1.011	1.027	0.268
count Age - 55-64 yrs	0.993	1.015	0.985	1.002	0.662	0.994	1.020	0.986	1.003	0.775
count Age - 65+ yrs	0.997	1.016	0.988	1.006	0.864	0.985	1.020	0.977	0.994	0.454
count Sex - male	1.006	1.011	1.000	1.013	0.545	0.998	1.013	0.992	1.004	0.887
count Social grade - C1C2	0.971	1.012	0.964	0.978	0.017	0.955	1.015	0.948	0.962	0.002
count Social grade - AB	0.940	1.015	0.932	0.949	<0.001	0.900	1.018	0.893	0.908	<0.001
count - Number of adults	0.992	1.006	0.989	0.995	0.159	0.992	1.007	0.989	0.995	0.212
count Presence of children - Yes	1.015	1.012	1.007	1.023	0.215	1.055	1.015	1.047	1.063	<0.001
count Region - North of England	1.013	1.009	1.007	1.019	0.175	1.058	1.012	1.051	1.064	<0.001
count Festival - Valentine's Day	1.022	1.007	1.005	1.040	0.001	1.014	1.006	0.998	1.031	0.016
count Festival - Easter	0.982	1.007	0.965	1.000	0.011	0.999	1.006	0.982	1.017	0.917
countFestival - Halloween	0.994	1.010	0.970	1.019	0.574	1.012	1.008	0.987	1.036	0.170
count Festival - Christmas	0.994	1.012	0.969	1.019	0.588	0.991	1.011	0.967	1.017	0.416
count Interaction Time*Pandemic	1.000	1.001	0.998	1.002	0.709	1.000	1.001	0.998	1.002	0.732
count Interaction Pandemic*Region	1.015	1.008	1.000	1.029	0.056	1.010	1.008	0.996	1.025	0.187
zero Constant	0.103	1.240	0.082	0.128	<0.001	0.026	1.320	0.018	0.036	<0.001
zero Time	0.998	1.001	0.995	1.001	0.226	0.997	1.002	0.992	1.001	0.153
zero Pandemic - during pandemic	1.161	1.145	0.894	1.507	0.271	1.327	1.207	0.908	1.941	0.133
zero Season - 2	0.966	1.065	0.850	1.097	0.578	1.014	1.108	0.837	1.230	0.889
zero Season - 3	0.967	1.067	0.848	1.103	0.608	1.164	1.101	0.960	1.412	0.115
zero Season - 4	0.814	1.084	0.697	0.950	0.010	0.893	1.117	0.705	1.130	0.306
zero Age - 45-54 yrs	0.619	1.153	0.543	0.706	0.001	0.651	1.238	0.541	0.785	0.044
zero Age - 55-64 yrs	0.520	1.170	0.453	0.596	<0.001	0.487	1.227	0.399	0.595	<0.001
zero Age - 65+ yrs	0.562	1.167	0.493	0.641	<0.001	0.530	1.226	0.438	0.642	0.002
zero Sex - male	1.228	1.115	1.119	1.347	0.060	1.215	1.156	1.061	1.392	0.179
zero Social grade - C1C2	1.059	1.144	0.935	1.200	0.671	1.300	1.190	1.063	1.589	0.131
zero Social grade - AB	1.337	1.166	1.161	1.540	0.059	1.852	1.217	1.488	2.305	0.002
zero - Number of adults	0.663	1.074	0.624	0.703	<0.001	0.788	1.089	0.727	0.854	0.005
zero Presence of children - Yes	0.454	1.147	0.396	0.522	<0.001	0.345	1.195	0.280	0.426	<0.001
zero Region - North of England	0.663	1.109	0.602	0.731	<0.001	0.604	1.144	0.522	0.698	<0.001
zero Festival - Valentine's Day	0.819	1.155	0.610	1.099	0.165	0.985	1.237	0.647	1.500	0.944
zero Festival - Easter	0.976	1.149	0.734	1.298	0.863	1.130	1.216	0.760	1.681	0.533
zeroFestival - Halloween	0.992	1.250	0.638	1.543	0.970	1.026	1.393	0.535	1.968	0.938
zero Festival - Christmas	2.002	1.197	1.426	2.810	<0.001	3.124	1.247	2.048	4.765	<0.001
zero Interaction Time*Pandemic	0.963	1.016	0.933	0.993	0.016	0.996	1.025	0.954	1.040	0.878
zero Interaction Pandemic*Region	1.268	1.161	1.003	1.602	0.113	0.962	1.220	0.688	1.346	0.847
Observations	89,382					89,382				

Outcome	Energy purchased from savoury snacks					Enegy purchased from chocolate & confectionery				
Term	Exp. estimate	SE	95%CI low	95%CI high	p value	Exp. estimate	SE	95%CI low	95%CI high	p value
count Constant	0.138	1.078	0.133	0.144	<0.001	0.151	1.064	0.145	0.157	<0.001
count Time	1.000	1.000	1.000	1.001	0.176	1.001	1.000	1.000	1.001	0.021
count Pandemic - during pandemic	0.930	1.030	0.889	0.973	0.013	0.952	1.033	0.907	0.999	0.125
count Season - 2	1.012	1.013	0.991	1.034	0.336	1.039	1.014	1.015	1.063	0.005
count Season - 3	1.065	1.013	1.042	1.089	<0.001	1.046	1.016	1.022	1.072	0.003
count Season - 4	1.079	1.015	1.053	1.105	<0.001	1.174	1.016	1.144	1.204	<0.001
count Age - 45-54 yrs	0.919	1.044	0.900	0.938	0.050	0.997	1.045	0.974	1.020	0.944
count Age - 55-64 yrs	0.813	1.052	0.795	0.833	<0.001	0.936	1.044	0.913	0.960	0.125
count Age - 65+ yrs	0.734	1.060	0.716	0.752	<0.001	0.870	1.046	0.847	0.893	0.002
count Sex - male	1.132	1.037	1.114	1.151	0.001	1.040	1.032	1.022	1.059	0.215
count Social grade - C1C2	0.906	1.041	0.888	0.923	0.014	0.899	1.038	0.880	0.917	0.004
count Social grade - AB	0.921	1.046	0.900	0.943	0.067	0.975	1.045	0.951	1.000	0.571
count - Number of adults	0.898	1.017	0.891	0.905	<0.001	0.872	1.018	0.865	0.880	<0.001
count Presence of children - Yes	0.804	1.043	0.789	0.820	<0.001	0.876	1.039	0.857	0.894	0.001
count Region - North of England	0.879	1.030	0.865	0.893	<0.001	0.979	1.029	0.963	0.997	0.475
count Festival - Valentine's Day	1.078	1.026	1.031	1.127	0.004	1.043	1.030	0.994	1.094	0.155
count Festival - Easter	0.969	1.026	0.924	1.015	0.207	1.192	1.030	1.137	1.249	<0.001
countFestival - Halloween	1.006	1.041	0.942	1.075	0.879	1.124	1.040	1.051	1.202	0.003
count Festival - Christmas	1.243	1.043	1.160	1.332	<0.001	1.168	1.057	1.081	1.263	0.005
count Interaction Time*Pandemic	1.000	1.003	0.995	1.005	0.993	0.988	1.003	0.983	0.994	<0.001
count Interaction Pandemic*Region	1.004	1.028	0.967	1.042	0.895	1.025	1.031	0.985	1.067	0.415
zero Constant	1.712	1.133	1.593	1.839	<0.001	1.651	1.123	1.538	1.774	<0.001
zero Time	0.999	1.001	0.998	1.000	0.144	0.999	1.001	0.998	1.000	0.068
zero Pandemic - during pandemic	0.959	1.047	0.879	1.047	0.366	0.732	1.049	0.671	0.799	<0.001
zero Season - 2	0.915	1.020	0.878	0.954	<0.001	0.901	1.021	0.865	0.939	<0.001
zero Season - 3	0.967	1.022	0.928	1.008	0.124	0.938	1.022	0.900	0.978	0.003
zero Season - 4	0.871	1.024	0.831	0.912	<0.001	0.712	1.024	0.680	0.746	<0.001
zero Age - 45-54 yrs	0.882	1.086	0.845	0.920	0.127	0.843	1.082	0.809	0.879	0.031
zero Age - 55-64 yrs	0.922	1.094	0.881	0.965	0.367	0.721	1.090	0.689	0.755	<0.001
zero Age - 65+ yrs	1.223	1.096	1.167	1.280	0.029	0.807	1.090	0.771	0.845	0.013
zero Sex - male	1.011	1.067	0.981	1.043	0.861	1.286	1.063	1.248	1.326	<0.001
zero Social grade - C1C2	0.907	1.081	0.874	0.942	0.210	1.057	1.075	1.018	1.097	0.446
zero Social grade - AB	1.037	1.094	0.991	1.084	0.691	1.249	1.089	1.195	1.306	0.009
zero - Number of adults	0.805	1.034	0.791	0.818	<0.001	0.888	1.033	0.873	0.902	<0.001
zero Presence of children - Yes	0.595	1.080	0.572	0.619	<0.001	0.676	1.075	0.650	0.703	<0.001
zero Region - North of England	0.978	1.059	0.948	1.008	0.692	0.747	1.057	0.724	0.770	<0.001
zero Festival - Valentine's Day	0.870	1.041	0.798	0.950	0.001	0.797	1.040	0.731	0.869	<0.001
zero Festival - Easter	1.021	1.043	0.932	1.119	0.616	0.695	1.045	0.633	0.764	<0.001
zeroFestival - Halloween	1.097	1.059	0.966	1.245	0.106	0.949	1.061	0.835	1.078	0.369
zero Festival - Christmas	1.227	1.064	1.078	1.396	0.001	1.699	1.067	1.493	1.933	<0.001
zero Interaction Time*Pandemic	0.990	1.005	0.980	1.000	0.029	1.009	1.005	0.999	1.019	0.055
zero Interaction Pandemic*Region	1.013	1.049	0.941	1.091	0.783	1.090	1.052	1.012	1.174	0.089
Observations	89,382					89,382				

Outcome	Energy purchased from low-sugar soft drinks					Energy from medium-sugar soft drinks				
Term	Exp. estimate	SE	95%CI low	95%CI high	p value	Exp. estimate	SE	95%CI low	95%CI high	p value
count Constant	0.021	1.210	0.019	0.022	<0.001	0.038	1.259	0.030	0.048	<0.001
count Time	0.999	1.001	0.998	1.000	0.421	1.004	1.002	1.001	1.007	0.079
count Pandemic - during pandemic	0.837	1.110	0.758	0.923	0.088	0.755	1.220	0.571	0.999	0.157
count Season - 2	1.005	1.046	0.960	1.053	0.910	0.892	1.087	0.780	1.021	0.171
count Season - 3	1.119	1.045	1.068	1.172	0.010	0.954	1.103	0.834	1.092	0.634
count Season - 4	1.000	1.042	0.949	1.054	0.997	0.910	1.127	0.790	1.048	0.426
count Age - 45-54 yrs	0.739	1.116	0.707	0.772	0.006	1.229	1.179	1.073	1.406	0.212
count Age - 55-64 yrs	0.860	1.148	0.819	0.903	0.275	1.179	1.159	1.015	1.369	0.265
count Age - 65+ yrs	0.674	1.140	0.639	0.710	0.003	0.973	1.176	0.837	1.130	0.865
count Sex - male	1.105	1.095	1.067	1.144	0.271	1.080	1.104	0.976	1.195	0.439
count Social grade - C1C2	0.742	1.124	0.712	0.773	0.010	0.655	1.221	0.577	0.743	0.033
count Social grade - AB	0.621	1.136	0.590	0.653	<0.001	0.689	1.219	0.593	0.800	0.059
count - Number of adults	0.909	1.047	0.895	0.923	0.037	0.843	1.062	0.803	0.884	0.005
count Presence of children - Yes	0.725	1.098	0.697	0.754	0.001	1.119	1.174	0.982	1.274	0.485
count Region - North of England	0.865	1.104	0.834	0.896	0.144	0.964	1.110	0.875	1.062	0.722
count Festival - Valentine's Day	1.080	1.078	0.977	1.193	0.309	1.440	1.236	1.086	1.911	0.085
count Festival - Easter	1.106	1.103	1.002	1.220	0.305	0.802	1.131	0.596	1.080	0.074
countFestival - Halloween	0.983	1.138	0.852	1.134	0.892	1.232	1.307	0.799	1.899	0.436
count Festival - Christmas	1.789	1.239	1.527	2.095	0.007	0.644	1.226	0.455	0.912	0.031
count Interaction Time*Pandemic	1.009	1.007	0.999	1.020	0.186	1.038	1.030	1.007	1.069	0.215
count Interaction Pandemic*Region	1.066	1.098	0.983	1.156	0.492	0.860	1.232	0.686	1.077	0.470
zero Constant	3.428	1.175	3.167	3.711	<0.001	51.109	1.328	40.165	65.035	<0.001
zero Time	1.000	1.001	0.999	1.001	0.794	0.996	1.002	0.993	0.999	0.069
zero Pandemic - during pandemic	0.873	1.051	0.793	0.961	0.006	1.367	1.184	1.017	1.839	0.064
zero Season - 2	0.868	1.022	0.830	0.908	<0.001	0.799	1.076	0.694	0.921	0.002
zero Season - 3	0.803	1.023	0.767	0.840	<0.001	0.852	1.086	0.740	0.981	0.051
zero Season - 4	0.946	1.024	0.899	0.995	0.018	0.737	1.082	0.638	0.853	<0.001
zero Age - 45-54 yrs	0.873	1.113	0.834	0.914	0.204	0.839	1.177	0.730	0.964	0.279
zero Age - 55-64 yrs	1.021	1.124	0.972	1.072	0.861	0.849	1.222	0.730	0.986	0.413
zero Age - 65+ yrs	1.292	1.124	1.228	1.359	0.029	0.984	1.219	0.841	1.151	0.935
zero Sex - male	1.183	1.084	1.144	1.223	0.037	1.289	1.152	1.160	1.432	0.073
zero Social grade - C1C2	0.956	1.104	0.918	0.995	0.645	0.822	1.197	0.723	0.936	0.277
zero Social grade - AB	1.343	1.123	1.279	1.410	0.011	0.844	1.213	0.725	0.982	0.379
zero - Number of adults	0.758	1.046	0.744	0.772	<0.001	0.943	1.076	0.896	0.992	0.423
zero Presence of children - Yes	0.654	1.103	0.626	0.682	<0.001	1.194	1.156	1.048	1.360	0.221
zero Region - North of England	0.549	1.076	0.531	0.568	<0.001	1.223	1.150	1.107	1.352	0.149
zero Festival - Valentine's Day	1.035	1.041	0.941	1.139	0.392	0.911	1.158	0.679	1.222	0.524
zero Festival - Easter	0.953	1.044	0.862	1.052	0.260	1.088	1.156	0.794	1.490	0.562
zeroFestival - Halloween	0.996	1.063	0.867	1.144	0.943	1.515	1.238	0.961	2.390	0.051
zero Festival - Christmas	1.216	1.069	1.054	1.402	0.004	0.918	1.203	0.635	1.327	0.643
zero Interaction Time*Pandemic	0.983	1.005	0.973	0.994	<0.001	0.968	1.016	0.938	0.999	0.040
zero Interaction Pandemic*Region	1.108	1.054	1.023	1.201	0.051	1.086	1.174	0.858	1.376	0.605
Observations	89,382					89,382				

Outcome	Energy from high-sugar soft drinks					Alcohol volume				
Term	Exp. estimate	SE	95%CI low	95%CI high	p value	Exp. estimate	SE	95%CI low	95%CI high	p value
count Constant	0.103	1.176	0.091	0.117	<0.001	3715.743	1.173	3475.589	3972.492	<0.001
count Time	0.999	1.001	0.997	1.001	0.377	1.001	1.001	1.000	1.002	0.058
count Pandemic - during pandemic	0.771	1.106	0.652	0.911	0.010	1.104	1.051	1.025	1.190	0.045
count Season - 2	0.926	1.056	0.856	1.002	0.162	1.098	1.022	1.060	1.138	<0.001
count Season - 3	1.146	1.074	1.056	1.244	0.056	1.093	1.027	1.055	1.133	0.001
count Season - 4	0.932	1.068	0.851	1.020	0.285	1.126	1.024	1.083	1.170	<0.001
count Age - 45-54 yrs	0.753	1.114	0.702	0.809	0.009	1.160	1.096	1.116	1.206	0.107
count Age - 55-64 yrs	0.630	1.141	0.584	0.681	<0.001	1.060	1.111	1.018	1.104	0.577
count Age - 65+ yrs	0.668	1.198	0.609	0.733	0.025	0.919	1.131	0.881	0.958	0.491
count Sex - male	1.151	1.104	1.082	1.224	0.156	1.112	1.075	1.084	1.141	0.144
count Social grade - C1C2	0.829	1.116	0.776	0.887	0.088	0.791	1.117	0.766	0.817	0.034
count Social grade - AB	0.796	1.116	0.730	0.866	0.038	0.722	1.121	0.695	0.750	0.004
count - Number of adults	0.853	1.056	0.832	0.876	0.003	0.695	1.038	0.686	0.704	<0.001
count Presence of children - Yes	0.690	1.091	0.645	0.738	<0.001	0.885	1.091	0.854	0.917	0.160
count Region - North of England	0.836	1.103	0.787	0.888	0.068	1.351	1.073	1.315	1.389	<0.001
count Festival - Valentine's Day	0.840	1.088	0.706	0.998	0.038	0.911	1.038	0.847	0.980	0.012
count Festival - Easter	0.952	1.094	0.819	1.106	0.579	1.085	1.040	1.009	1.167	0.038
countFestival - Halloween	0.834	1.120	0.653	1.064	0.108	0.911	1.058	0.821	1.011	0.099
count Festival - Christmas	0.837	1.124	0.674	1.039	0.127	1.036	1.064	0.934	1.150	0.564
count Interaction Time*Pandemic	1.014	1.011	0.995	1.032	0.231	0.995	1.005	0.987	1.002	0.275
count Interaction Pandemic*Region	1.107	1.139	0.965	1.269	0.436	1.092	1.052	1.029	1.160	0.084
zero Constant	10.376	1.334	8.962	12.013	<0.001	8.343	1.189	7.698	9.043	<0.001
zero Time	1.001	1.001	0.999	1.003	0.304	0.999	1.001	0.998	1.000	0.320
zero Pandemic - during pandemic	1.013	1.103	0.841	1.219	0.898	0.863	1.055	0.785	0.949	0.006
zero Season - 2	0.844	1.048	0.773	0.922	<0.001	0.829	1.022	0.793	0.867	<0.001
zero Season - 3	0.891	1.048	0.813	0.976	0.014	0.816	1.024	0.779	0.854	<0.001
zero Season - 4	0.843	1.054	0.763	0.933	0.001	0.687	1.025	0.654	0.721	<0.001
zero Age - 45-54 yrs	1.691	1.201	1.558	1.835	0.004	0.765	1.125	0.730	0.801	0.022
zero Age - 55-64 yrs	1.792	1.249	1.641	1.957	0.009	0.675	1.135	0.643	0.709	0.002
zero Age - 65+ yrs	3.134	1.268	2.824	3.477	<0.001	0.725	1.138	0.689	0.762	0.013
zero Sex - male	0.888	1.157	0.832	0.949	0.416	1.011	1.092	0.978	1.044	0.903
zero Social grade - C1C2	1.562	1.185	1.453	1.680	0.008	0.782	1.119	0.751	0.815	0.029
zero Social grade - AB	1.992	1.213	1.814	2.189	<0.001	0.801	1.139	0.763	0.841	0.087
zero - Number of adults	0.810	1.078	0.785	0.835	0.005	0.843	1.048	0.829	0.857	<0.001
zero Presence of children - Yes	1.238	1.197	1.147	1.338	0.234	1.337	1.115	1.281	1.396	0.007
zero Region - North of England	1.369	1.145	1.283	1.462	0.021	0.622	1.085	0.601	0.643	<0.001
zero Festival - Valentine's Day	0.963	1.087	0.794	1.168	0.649	0.820	1.039	0.747	0.901	<0.001
zero Festival - Easter	0.715	1.078	0.604	0.848	<0.001	0.975	1.039	0.887	1.073	0.512
zeroFestival - Halloween	1.052	1.125	0.801	1.382	0.667	1.198	1.056	1.048	1.369	0.001
zero Festival - Christmas	0.738	1.126	0.578	0.943	0.011	1.198	1.068	1.048	1.371	0.006
zero Interaction Time*Pandemic	0.988	1.010	0.968	1.008	0.226	0.986	1.004	0.976	0.996	0.001
zero Interaction Pandemic*Region	0.978	1.110	0.839	1.140	0.833	0.921	1.059	0.852	0.995	0.153
Observations	89,382					89,382				

Outcome	OOH purchasing				
Term	Exp. estimate	SE	95%CI low	95%CI high	p value
count Constant	1.375	1.352	1.250	1.513	0.290
count Time	0.999	1.001	0.997	1.000	0.037
count Pandemic - during pandemic	0.495	1.102	0.434	0.563	<0.001
count Season - 2	0.962	1.025	0.913	1.013	0.121
count Season - 3	0.995	1.024	0.945	1.047	0.824
count Season - 4	0.995	1.024	0.940	1.053	0.829
count Age - 45-54 yrs	0.913	1.181	0.871	0.957	0.582
count Age - 55-64 yrs	1.046	1.191	0.993	1.102	0.797
count Age - 65+ yrs	0.718	1.261	0.668	0.772	0.154
count Sex - male	1.503	1.137	1.446	1.562	0.001
count Social grade - C1C2	1.024	1.218	0.971	1.079	0.905
count Social grade - AB	1.031	1.299	0.963	1.104	0.907
count - Number of adults	1.005	1.078	0.983	1.027	0.948
count Presence of children - Yes	0.995	1.173	0.950	1.042	0.975
count Region - North of England	1.106	1.126	1.064	1.149	0.398
count Festival - Valentine's Day	1.006	1.027	0.906	1.117	0.827
count Festival - Easter	0.874	1.042	0.772	0.990	0.001
countFestival - Halloween	1.012	1.041	0.872	1.175	0.767
count Festival - Christmas	0.727	1.060	0.616	0.859	<0.001
count Interaction Time*Pandemic	1.018	1.008	1.004	1.033	0.026
count Interaction Pandemic*Region	1.023	1.128	0.918	1.139	0.852
zero Constant	0.073	3.975	0.031	0.170	0.058
zero Time	1.008	1.013	0.993	1.023	0.543
zero Pandemic - during pandemic	2.599	2.056	0.953	7.091	0.185
zero Season - 2	2.529	1.643	1.444	4.428	0.062
zero Season - 3	1.050	1.435	0.574	1.920	0.893
zero Season - 4	1.305	1.473	0.705	2.415	0.491
zero Age - 45-54 yrs	-				
zero Age - 55-64 yrs	-				
zero Age - 65+ yrs	-				
zero Sex - male	8.395	3.566	4.992	14.119	0.094
zero Social grade - C1C2	0.001	8.797	<0.001	9335.695	0.001
zero Social grade - AB	5.620	4.422	3.211	9.835	0.246
zero - Number of adults	0.359	2.159	0.279	0.461	0.183
zero Presence of children - Yes	-				
zero Region - North of England	-				
zero Festival - Valentine's Day	-				
zero Festival - Easter	-				
zeroFestival - Halloween	-				
zero Festival - Christmas	-				
zero Interaction Time*Pandemic	0.800	1.106	0.690	0.928	0.027
zero Interaction Pandemic*Region	-				
Observations	16,806				

Model coefficients from secondary analysis; interactions with presence of children

HFSS = high in fat, salt and sugar; UPF = ultra-processed food; OOH = out-of-home

Due to multicollineraity, the variables region, presence of children, and age of the main shopper were not included in the OOH models

Outcome	Total Energy purchased					Energy purchased from fruit & vegetables				
Term	Exp. estimate	SE	95%CI low	95%CI high	p value	Exp. estimate	SE	95%CI low	95%CI high	p value
count Constant	15168.394	1.046	14814.241	15531.013	<0.001	0.082	1.089	0.080	0.085	<0.001
count Time	1.001	1.000	1.000	1.001	<0.001	0.999	1.000	0.998	0.999	<0.001
count Pandemic - during pandemic	1.178	1.013	1.147	1.210	<0.001	0.907	1.025	0.875	0.939	<0.001
count Season - 2	1.007	1.006	0.993	1.021	0.222	1.029	1.012	1.010	1.048	0.022
count Season - 3	0.970	1.007	0.956	0.983	<0.001	1.025	1.013	1.006	1.045	0.053
count Season - 4	1.070	1.007	1.054	1.087	<0.001	0.894	1.013	0.876	0.913	<0.001
count Age - 45-54 yrs	1.147	1.029	1.131	1.163	<0.001	0.911	1.051	0.894	0.929	0.060
count Age - 55-64 yrs	1.255	1.032	1.236	1.274	<0.001	0.862	1.060	0.844	0.880	0.011
count Age - 65+ yrs	1.271	1.032	1.251	1.290	<0.001	0.867	1.061	0.849	0.885	0.017
count Sex - male	0.971	1.023	0.961	0.981	0.199	0.971	1.038	0.958	0.985	0.432
count Social grade - C1C2	1.002	1.029	0.990	1.014	0.945	1.144	1.045	1.125	1.164	0.002
count Social grade - AB	0.928	1.034	0.914	0.941	0.026	1.422	1.053	1.394	1.451	<0.001
count - Number of adults	0.864	1.012	0.859	0.868	<0.001	0.890	1.021	0.883	0.896	<0.001
count Presence of children - Yes	0.799	1.027	0.788	0.810	<0.001	0.826	1.048	0.811	0.842	<0.001
count Region - North of England	1.042	1.020	1.033	1.052	0.039	0.757	1.035	0.748	0.767	<0.001
count Festival - Valentine's Day	0.998	1.012	0.970	1.027	0.868	0.926	1.020	0.890	0.963	<0.001
count Festival - Easter	1.042	1.013	1.011	1.074	0.001	0.983	1.025	0.944	1.024	0.488
countFestival - Halloween	0.922	1.017	0.884	0.961	<0.001	1.067	1.039	1.008	1.131	0.084
count Festival - Christmas	0.855	1.025	0.819	0.893	<0.001	1.076	1.046	1.013	1.143	0.106
count Interaction Time*Pandemic	0.999	1.001	0.996	1.002	0.320	1.000	1.003	0.996	1.004	0.939
count Interaction Pandemic*Presence of Children - Yes	1.056	1.020	1.027	1.086	0.006	1.000	1.032	0.963	1.039	0.990
zero Constant	0.078	1.176	0.067	0.092	<0.001	0.471	1.199	0.421	0.526	<0.001
zero Time	0.998	1.001	0.996	1.001	0.146	1.000	1.001	0.999	1.002	0.893
zero Pandemic - during pandemic	1.441	1.091	1.211	1.715	<0.001	0.865	1.075	0.755	0.992	0.046
zero Season - 2	1.060	1.045	0.964	1.165	0.184	0.977	1.033	0.913	1.046	0.480
zero Season - 3	1.411	1.041	1.289	1.545	<0.001	1.040	1.037	0.973	1.113	0.281
zero Season - 4	1.120	1.053	1.005	1.247	0.029	1.182	1.037	1.100	1.271	<0.001
zero Age - 45-54 yrs	0.737	1.098	0.677	0.803	0.001	0.834	1.120	0.780	0.891	0.108
zero Age - 55-64 yrs	0.549	1.122	0.499	0.605	<0.001	0.694	1.139	0.646	0.745	0.005
zero Age - 65+ yrs	0.466	1.123	0.422	0.516	<0.001	0.472	1.141	0.438	0.509	<0.001
zero Sex - male	0.871	1.083	0.812	0.934	0.084	1.335	1.094	1.273	1.401	0.001
zero Social grade - C1C2	0.980	1.106	0.900	1.066	0.838	0.631	1.111	0.597	0.667	<0.001
zero Social grade - AB	1.109	1.121	1.005	1.223	0.367	0.550	1.143	0.512	0.591	<0.001
zero - Number of adults	0.922	1.044	0.889	0.957	0.057	0.689	1.066	0.669	0.710	<0.001
zero Presence of children - Yes	0.861	1.095	0.789	0.939	0.099	0.673	1.118	0.628	0.721	<0.001
zero Region - North of England	0.995	1.069	0.936	1.058	0.941	1.107	1.086	1.057	1.158	0.221
zero Festival - Valentine's Day	0.937	1.114	0.758	1.159	0.547	1.099	1.061	0.959	1.260	0.112
zero Festival - Easter	1.251	1.098	1.043	1.501	0.016	0.965	1.080	0.824	1.130	0.640
zeroFestival - Halloween	1.641	1.122	1.297	2.076	<0.001	0.879	1.099	0.718	1.076	0.173
zero Festival - Christmas	2.542	1.103	2.073	3.118	<0.001	1.327	1.093	1.106	1.591	0.002
zero Interaction Time*Pandemic	0.979	1.010	0.959	0.999	0.029	0.987	1.009	0.970	1.004	0.115
zero Interaction Pandemic*Presence of Children - Yes	1.035	1.097	0.871	1.229	0.714	0.876	1.102	0.743	1.033	0.172
Observations	89,382					89,382				

Outcome	Energy purchased from HFSS					Energy purchased from UPF				
Term	Exp. estimate	SE	95%CI low	95%CI high	p value	Exp. estimate	SE	95%CI low	95%CI high	p value
count Constant	0.519	1.021	0.512	0.527	<0.001	0.603	1.026	0.594	0.611	<0.001
count Time	1.000	1.000	1.000	1.000	0.776	1.000	1.000	1.000	1.000	0.765
count Pandemic - during pandemic	1.008	1.007	0.993	1.024	0.228	0.964	1.007	0.950	0.979	<0.001
count Season - 2	1.033	1.003	1.025	1.042	<0.001	1.017	1.003	1.009	1.025	<0.001
count Season - 3	1.039	1.004	1.031	1.048	<0.001	1.024	1.003	1.016	1.032	<0.001
count Season - 4	1.073	1.004	1.064	1.083	<0.001	1.026	1.003	1.017	1.036	<0.001
count Age - 45-54 yrs	0.998	1.013	0.990	1.007	0.904	1.019	1.017	1.011	1.027	0.267
count Age - 55-64 yrs	0.993	1.015	0.985	1.002	0.664	0.995	1.020	0.986	1.003	0.776
count Age - 65+ yrs	0.997	1.016	0.988	1.006	0.863	0.985	1.020	0.977	0.994	0.453
count Sex - male	1.006	1.011	1.000	1.012	0.547	0.998	1.013	0.992	1.004	0.885
count Social grade - C1C2	0.971	1.012	0.964	0.978	0.017	0.955	1.015	0.948	0.962	0.002
count Social grade - AB	0.940	1.015	0.932	0.949	<0.001	0.900	1.018	0.893	0.908	<0.001
count - Number of adults	0.992	1.006	0.989	0.995	0.159	0.992	1.007	0.989	0.995	0.212
count Presence of children - Yes	1.011	1.012	1.003	1.019	0.384	1.051	1.015	1.043	1.059	0.001
count Region - North of England	1.015	1.009	1.010	1.021	0.095	1.059	1.012	1.054	1.065	<0.001
count Festival - Valentine's Day	1.022	1.007	1.005	1.040	0.001	1.014	1.006	0.997	1.031	0.016
count Festival - Easter	0.982	1.007	0.965	1.000	0.011	0.999	1.006	0.982	1.017	0.912
countFestival - Halloween	0.994	1.010	0.970	1.019	0.571	1.011	1.008	0.987	1.036	0.172
count Festival - Christmas	0.994	1.012	0.969	1.019	0.589	0.991	1.011	0.967	1.017	0.414
count Interaction Time*Pandemic	1.000	1.001	0.998	1.002	0.735	1.000	1.001	0.998	1.002	0.758
count Interaction Pandemic*Presence of Children - Yes	1.025	1.008	1.008	1.042	0.002	1.022	1.008	1.006	1.039	0.006
zero Constant	0.101	1.240	0.081	0.126	<0.001	0.026	1.318	0.019	0.037	<0.001
zero Time	0.998	1.001	0.995	1.001	0.223	0.997	1.002	0.992	1.001	0.153
zero Pandemic - during pandemic	1.271	1.138	1.000	1.615	0.063	1.207	1.202	0.841	1.733	0.306
zero Season - 2	0.966	1.065	0.850	1.097	0.578	1.014	1.108	0.836	1.229	0.892
zero Season - 3	0.967	1.067	0.848	1.103	0.607	1.163	1.101	0.959	1.411	0.117
zero Season - 4	0.813	1.084	0.697	0.950	0.010	0.892	1.117	0.705	1.129	0.304
zero Age - 45-54 yrs	0.620	1.153	0.544	0.707	0.001	0.651	1.237	0.541	0.784	0.044
zero Age - 55-64 yrs	0.520	1.170	0.454	0.597	<0.001	0.487	1.227	0.399	0.595	<0.001
zero Age - 65+ yrs	0.562	1.167	0.493	0.641	<0.001	0.530	1.226	0.438	0.641	0.002
zero Sex - male	1.228	1.115	1.119	1.347	0.060	1.215	1.156	1.061	1.391	0.181
zero Social grade - C1C2	1.060	1.144	0.935	1.201	0.667	1.300	1.190	1.063	1.589	0.131
zero Social grade - AB	1.338	1.166	1.161	1.541	0.058	1.852	1.217	1.488	2.305	0.002
zero - Number of adults	0.663	1.074	0.625	0.703	<0.001	0.788	1.089	0.727	0.854	0.005
zero Presence of children - Yes	0.443	1.150	0.382	0.515	<0.001	0.309	1.207	0.244	0.392	<0.001
zero Region - North of England	0.690	1.103	0.632	0.754	<0.001	0.600	1.131	0.526	0.684	<0.001
zero Festival - Valentine's Day	0.819	1.155	0.610	1.099	0.165	0.985	1.237	0.647	1.500	0.943
zero Festival - Easter	0.976	1.148	0.734	1.297	0.859	1.129	1.216	0.759	1.679	0.536
zeroFestival - Halloween	0.992	1.250	0.638	1.543	0.972	1.026	1.393	0.535	1.968	0.939
zero Festival - Christmas	2.002	1.197	1.427	2.810	<0.001	3.124	1.247	2.048	4.766	<0.001
zero Interaction Time*Pandemic	0.963	1.016	0.933	0.993	0.016	0.996	1.025	0.954	1.041	0.885
zero Interaction Pandemic*Presence of Children - Yes	1.155	1.209	0.845	1.579	0.447	1.676	1.303	1.076	2.610	0.051
Observations	89,382					89,382				

Outcome	Energy purchased from savoury snacks					Enegy purchased from chocolate & confectionery				
Term	Exp. estimate	SE	95%CI low	95%CI high	p value	Exp. estimate	SE	95%CI low	95%CI high	p value
count Constant	0.139	1.077	0.134	0.144	<0.001	0.150	1.064	0.144	0.156	<0.001
count Time	1.000	1.000	1.000	1.001	0.176	1.001	1.000	1.000	1.001	0.021
count Pandemic - during pandemic	0.909	1.026	0.872	0.948	<0.001	0.965	1.028	0.923	1.008	0.186
count Season - 2	1.012	1.013	0.991	1.034	0.333	1.039	1.014	1.015	1.063	0.005
count Season - 3	1.065	1.013	1.041	1.088	<0.001	1.046	1.016	1.022	1.072	0.003
count Season - 4	1.079	1.015	1.053	1.105	<0.001	1.174	1.016	1.144	1.204	<0.001
count Age - 45-54 yrs	0.919	1.044	0.900	0.938	0.050	0.997	1.045	0.974	1.021	0.947
count Age - 55-64 yrs	0.813	1.052	0.795	0.832	<0.001	0.936	1.044	0.913	0.961	0.127
count Age - 65+ yrs	0.734	1.060	0.716	0.752	<0.001	0.870	1.046	0.847	0.893	0.002
count Sex - male	1.132	1.037	1.114	1.150	0.001	1.040	1.032	1.022	1.059	0.215
count Social grade - C1C2	0.906	1.041	0.888	0.923	0.014	0.899	1.038	0.880	0.917	0.004
count Social grade - AB	0.921	1.046	0.900	0.943	0.068	0.975	1.045	0.951	1.000	0.572
count - Number of adults	0.898	1.017	0.891	0.905	<0.001	0.872	1.018	0.864	0.880	<0.001
count Presence of children - Yes	0.793	1.043	0.777	0.810	<0.001	0.875	1.040	0.856	0.895	0.001
count Region - North of England	0.880	1.029	0.867	0.892	<0.001	0.984	1.028	0.969	1.000	0.558
count Festival - Valentine's Day	1.078	1.026	1.031	1.127	0.004	1.043	1.030	0.994	1.094	0.154
count Festival - Easter	0.968	1.026	0.924	1.015	0.202	1.192	1.030	1.137	1.249	<0.001
countFestival - Halloween	1.006	1.041	0.942	1.075	0.882	1.124	1.040	1.051	1.201	0.003
count Festival - Christmas	1.242	1.043	1.159	1.331	<0.001	1.169	1.057	1.081	1.263	0.005
count Interaction Time*Pandemic	1.000	1.003	0.995	1.005	0.930	0.988	1.003	0.983	0.994	<0.001
count Interaction Pandemic*Presence of Children - Yes	1.077	1.029	1.035	1.122	0.009	1.003	1.030	0.960	1.049	0.909
zero Constant	1.703	1.133	1.586	1.830	<0.001	1.632	1.123	1.520	1.753	<0.001
zero Time	0.999	1.001	0.998	1.000	0.145	0.999	1.001	0.998	1.000	0.068
zero Pandemic - during pandemic	0.986	1.041	0.911	1.068	0.729	0.782	1.044	0.722	0.847	<0.001
zero Season - 2	0.915	1.020	0.878	0.954	<0.001	0.902	1.021	0.865	0.939	<0.001
zero Season - 3	0.967	1.022	0.928	1.009	0.126	0.938	1.022	0.900	0.978	0.003
zero Season - 4	0.871	1.024	0.831	0.912	<0.001	0.713	1.024	0.681	0.746	<0.001
zero Age - 45-54 yrs	0.882	1.086	0.845	0.920	0.127	0.843	1.082	0.809	0.880	0.031
zero Age - 55-64 yrs	0.922	1.094	0.881	0.965	0.367	0.722	1.090	0.690	0.755	<0.001
zero Age - 65+ yrs	1.223	1.096	1.167	1.280	0.029	0.808	1.090	0.771	0.846	0.013
zero Sex - male	1.011	1.067	0.981	1.043	0.860	1.286	1.063	1.248	1.326	<0.001
zero Social grade - C1C2	0.907	1.081	0.874	0.942	0.211	1.057	1.075	1.018	1.097	0.445
zero Social grade - AB	1.037	1.094	0.992	1.084	0.690	1.250	1.089	1.196	1.306	0.009
zero - Number of adults	0.805	1.034	0.791	0.818	<0.001	0.888	1.033	0.874	0.902	<0.001
zero Presence of children - Yes	0.604	1.080	0.579	0.629	<0.001	0.685	1.076	0.657	0.714	<0.001
zero Region - North of England	0.980	1.057	0.953	1.007	0.714	0.757	1.056	0.737	0.779	<0.001
zero Festival - Valentine's Day	0.871	1.041	0.798	0.950	0.001	0.798	1.040	0.732	0.870	<0.001
zero Festival - Easter	1.021	1.043	0.932	1.120	0.612	0.695	1.045	0.633	0.764	<0.001
zeroFestival - Halloween	1.097	1.059	0.966	1.245	0.106	0.949	1.060	0.835	1.078	0.370
zero Festival - Christmas	1.227	1.064	1.078	1.396	0.001	1.698	1.067	1.493	1.932	<0.001
zero Interaction Time*Pandemic	0.990	1.005	0.980	0.999	0.028	1.009	1.005	0.999	1.019	0.056
zero Interaction Pandemic*Presence of Children - Yes	0.916	1.056	0.839	1.001	0.107	0.926	1.056	0.849	1.009	0.158
Observations	89,382					89,382				

Outcome	Energy purchased from low-sugar soft drinks					Energy from medium-sugar soft drinks				
Term	Exp. estimate	SE	95%CI low	95%CI high	p value	Exp. estimate	SE	95%CI low	95%CI high	p value
count Constant	0.020	1.205	0.019	0.022	<0.001	0.040	1.253	0.032	0.051	<0.001
count Time	0.999	1.001	0.998	1.000	0.420	1.005	1.002	1.001	1.008	0.065
count Pandemic - during pandemic	0.879	1.078	0.805	0.959	0.086	0.635	1.199	0.493	0.818	0.012
count Season - 2	1.005	1.046	0.960	1.053	0.912	0.909	1.087	0.795	1.039	0.253
count Season - 3	1.119	1.045	1.068	1.172	0.010	0.951	1.102	0.832	1.087	0.601
count Season - 4	1.000	1.042	0.949	1.054	0.991	0.908	1.126	0.790	1.045	0.418
count Age - 45-54 yrs	0.738	1.116	0.706	0.772	0.006	1.169	1.171	1.021	1.338	0.323
count Age - 55-64 yrs	0.860	1.149	0.819	0.903	0.278	1.147	1.154	0.988	1.332	0.339
count Age - 65+ yrs	0.674	1.140	0.639	0.710	0.003	0.938	1.172	0.807	1.089	0.684
count Sex - male	1.105	1.095	1.067	1.144	0.272	1.089	1.102	0.985	1.204	0.381
count Social grade - C1C2	0.743	1.124	0.712	0.774	0.011	0.657	1.213	0.580	0.744	0.030
count Social grade - AB	0.622	1.136	0.590	0.654	<0.001	0.695	1.211	0.600	0.806	0.058
count - Number of adults	0.909	1.047	0.895	0.923	0.037	0.846	1.062	0.807	0.888	0.005
count Presence of children - Yes	0.729	1.102	0.698	0.760	0.001	0.945	1.158	0.821	1.086	0.697
count Region - North of England	0.875	1.097	0.848	0.904	0.148	0.939	1.098	0.860	1.025	0.500
count Festival - Valentine's Day	1.080	1.079	0.977	1.194	0.309	1.427	1.227	1.078	1.889	0.083
count Festival - Easter	1.106	1.104	1.003	1.221	0.306	0.764	1.137	0.568	1.027	0.036
countFestival - Halloween	0.982	1.138	0.852	1.133	0.891	1.268	1.338	0.825	1.950	0.415
count Festival - Christmas	1.792	1.239	1.530	2.099	0.007	0.658	1.229	0.466	0.929	0.042
count Interaction Time*Pandemic	1.009	1.007	0.999	1.020	0.185	1.019	1.023	0.989	1.050	0.403
count Interaction Pandemic*Presence of Children - Yes	0.973	1.087	0.894	1.060	0.746	2.120	1.281	1.617	2.779	0.002
zero Constant	3.395	1.175	3.137	3.673	<0.001	50.390	1.325	39.634	64.063	<0.001
zero Time	1.000	1.001	0.999	1.001	0.793	0.996	1.002	0.993	0.999	0.069
zero Pandemic - during pandemic	0.920	1.044	0.843	1.004	0.051	1.481	1.178	1.120	1.959	0.016
zero Season - 2	0.868	1.022	0.830	0.908	<0.001	0.800	1.076	0.694	0.921	0.002
zero Season - 3	0.803	1.023	0.767	0.840	<0.001	0.852	1.086	0.740	0.981	0.052
zero Season - 4	0.946	1.024	0.900	0.995	0.018	0.738	1.082	0.638	0.853	<0.001
zero Age - 45-54 yrs	0.873	1.113	0.834	0.915	0.205	0.839	1.176	0.730	0.964	0.279
zero Age - 55-64 yrs	1.021	1.124	0.972	1.073	0.858	0.849	1.222	0.730	0.986	0.413
zero Age - 65+ yrs	1.292	1.124	1.228	1.359	0.029	0.984	1.219	0.841	1.151	0.935
zero Sex - male	1.183	1.084	1.144	1.223	0.037	1.289	1.152	1.160	1.432	0.073
zero Social grade - C1C2	0.956	1.104	0.918	0.995	0.647	0.823	1.197	0.723	0.936	0.277
zero Social grade - AB	1.343	1.123	1.279	1.410	0.011	0.844	1.213	0.725	0.982	0.380
zero - Number of adults	0.758	1.046	0.744	0.772	<0.001	0.943	1.076	0.896	0.992	0.423
zero Presence of children - Yes	0.651	1.104	0.622	0.681	<0.001	1.229	1.162	1.068	1.415	0.169
zero Region - North of England	0.559	1.074	0.542	0.576	<0.001	1.241	1.143	1.134	1.359	0.106
zero Festival - Valentine's Day	1.035	1.041	0.941	1.139	0.393	0.910	1.158	0.679	1.222	0.522
zero Festival - Easter	0.952	1.044	0.862	1.052	0.258	1.088	1.156	0.794	1.490	0.559
zeroFestival - Halloween	0.996	1.063	0.867	1.144	0.942	1.516	1.238	0.961	2.390	0.051
zero Festival - Christmas	1.216	1.069	1.054	1.402	0.004	0.918	1.203	0.635	1.328	0.645
zero Interaction Time*Pandemic	0.983	1.005	0.973	0.993	<0.001	0.968	1.016	0.938	0.999	0.040
zero Interaction Pandemic*Presence of Children - Yes	1.029	1.061	0.937	1.130	0.631	0.850	1.202	0.643	1.123	0.376
Observations	89,382					89,382				

Term	Energy from high-sugar soft drinks: Exp. estimate	SE	95%CI low	95%CI high	p value	Alcohol volume: Exp. estimate	SE	95%CI low	95%CI high	p value
count Constant	0.110	1.168	0.097	0.124	<0.001	3685.240	1.172	3448.575	3938.146	<0.001
count Time	0.999	1.001	0.997	1.001	0.369	1.001	1.001	1.000	1.002	0.057
count Pandemic - during pandemic	0.329	2.138	0.092	1.181	0.144	1.148	1.045	1.075	1.225	0.002
count Season - 2	0.926	1.056	0.856	1.002	0.160	1.098	1.022	1.060	1.138	<0.001
count Season - 3	1.145	1.074	1.055	1.243	0.057	1.093	1.027	1.055	1.133	0.001
count Season - 4	0.933	1.068	0.852	1.021	0.292	1.125	1.024	1.083	1.170	<0.001
count Age - 45-54 yrs	0.752	1.114	0.701	0.808	0.008	1.161	1.096	1.117	1.207	0.105
count Age - 55-64 yrs	0.630	1.141	0.583	0.680	<0.001	1.059	1.110	1.016	1.103	0.585
count Age - 65+ yrs	0.667	1.198	0.608	0.731	0.025	0.918	1.130	0.880	0.957	0.485
count Sex - male	1.151	1.104	1.082	1.224	0.157	1.113	1.075	1.084	1.142	0.141
count Social grade - C1C2	0.830	1.115	0.777	0.888	0.088	0.792	1.116	0.767	0.818	0.034
count Social grade - AB	0.796	1.116	0.731	0.867	0.038	0.723	1.121	0.695	0.751	0.004
count - Number of adults	0.854	1.055	0.832	0.876	0.003	0.695	1.038	0.686	0.705	<0.001
count Presence of children - Yes	0.692	1.099	0.643	0.744	<0.001	0.867	1.094	0.834	0.902	0.114
count Region - North of England	0.852	1.097	0.806	0.900	0.082	1.375	1.070	1.342	1.409	<0.001
count Festival - Valentine's Day	0.840	1.088	0.706	0.998	0.038	0.911	1.038	0.847	0.981	0.012
count Festival - Easter	0.946	1.093	0.814	1.100	0.534	1.083	1.040	1.008	1.165	0.041
countFestival - Halloween	0.833	1.119	0.653	1.064	0.105	0.910	1.058	0.820	1.010	0.095
count Festival - Christmas	0.837	1.124	0.674	1.039	0.126	1.038	1.064	0.935	1.152	0.552
count Interaction Time*Pandemic	1.014	1.011	0.996	1.033	0.205	0.995	1.005	0.987	1.002	0.269
count Interaction Pandemic*Presence of Children - Yes	0.982	1.153	0.844	1.143	0.898	1.092	1.055	1.019	1.170	0.101
zero Constant	9.558	1.337	8.309	10.996	<0.001	8.355	1.189	7.711	9.052	<0.001
zero Time	1.001	1.001	0.999	1.003	0.304	0.999	1.001	0.998	1.000	0.321
zero Pandemic - during pandemic	2.138	1.984	0.524	8.723	0.267	0.859	1.044	0.790	0.935	<0.001
zero Season - 2	0.844	1.048	0.773	0.922	<0.001	0.829	1.022	0.793	0.867	<0.001
zero Season - 3	0.891	1.048	0.813	0.976	0.014	0.816	1.024	0.779	0.854	<0.001
zero Season - 4	0.843	1.054	0.763	0.932	0.001	0.686	1.025	0.653	0.721	<0.001
zero Age - 45-54 yrs	1.691	1.201	1.558	1.835	0.004	0.764	1.125	0.730	0.800	0.022
zero Age - 55-64 yrs	1.792	1.249	1.641	1.957	0.009	0.675	1.136	0.643	0.709	0.002
zero Age - 65+ yrs	3.133	1.268	2.824	3.477	<0.001	0.725	1.138	0.689	0.762	0.013
zero Sex - male	0.888	1.157	0.832	0.949	0.416	1.011	1.092	0.978	1.045	0.902
zero Social grade - C1C2	1.562	1.185	1.453	1.680	0.008	0.782	1.119	0.751	0.815	0.029
zero Social grade - AB	1.992	1.213	1.813	2.189	<0.001	0.801	1.139	0.763	0.841	0.087
zero - Number of adults	0.810	1.078	0.785	0.835	0.005	0.843	1.048	0.829	0.857	<0.001
zero Presence of children - Yes	1.228	1.201	1.131	1.334	0.261	1.391	1.118	1.328	1.457	0.003
zero Region - North of England	1.364	1.141	1.286	1.447	0.018	0.612	1.083	0.594	0.631	<0.001
zero Festival - Valentine's Day	0.963	1.087	0.794	1.168	0.649	0.820	1.039	0.746	0.901	<0.001
zero Festival - Easter	0.715	1.078	0.603	0.847	<0.001	0.976	1.039	0.887	1.074	0.525
zeroFestival - Halloween	1.052	1.125	0.801	1.382	0.668	1.198	1.056	1.048	1.370	0.001
zero Festival - Christmas	0.738	1.126	0.578	0.943	0.011	1.199	1.068	1.048	1.371	0.006
zero Interaction Time*Pandemic	0.988	1.010	0.968	1.008	0.225	0.986	1.004	0.976	0.996	0.001
zero Interaction Pandemic*Presence of Children - Yes	1.048	1.125	0.884	1.242	0.693	0.815	1.068	0.745	0.891	0.002
Observations	89,382					89,382				

Outcome	OOH purchasing				
Term	Exp. estimate	SE	95%CI low	95%CI high	p value
count Constant	1.377	1.351	1.252	1.515	0.287
count Time	0.999	1.001	0.997	1.000	0.037
count Pandemic - during pandemic	0.490	1.091	0.436	0.550	<0.001
count Season - 2	0.962	1.025	0.913	1.013	0.121
count Season - 3	0.995	1.024	0.945	1.046	0.819
count Season - 4	0.995	1.024	0.940	1.053	0.828
count Age - 45-54 yrs	0.912	1.181	0.870	0.956	0.580
count Age - 55-64 yrs	1.045	1.191	0.992	1.101	0.799
count Age - 65+ yrs	0.717	1.261	0.667	0.771	0.152
count Sex - male	1.503	1.137	1.446	1.563	0.001
count Social grade - C1C2	1.025	1.218	0.972	1.080	0.902
count Social grade - AB	1.031	1.299	0.962	1.104	0.908
count - Number of adults	1.005	1.078	0.983	1.027	0.948
count Presence of children - Yes	0.985	1.171	0.938	1.034	0.923
count Region - North of England	1.108	1.125	1.069	1.149	0.382
count Festival - Valentine's Day	1.006	1.027	0.906	1.117	0.823
count Festival - Easter	0.874	1.042	0.772	0.990	0.001
countFestival - Halloween	1.012	1.041	0.872	1.174	0.770
count Festival - Christmas	0.727	1.060	0.616	0.859	<0.001
count Interaction Time*Pandemic	1.018	1.008	1.004	1.033	0.025
count Interaction Pandemic*Presence of Children - Yes	1.086	1.138	0.967	1.218	0.526
zero Constant	0.072	4.042	0.031	0.168	0.059
zero Time	1.008	1.013	0.993	1.023	0.539
zero Pandemic - during pandemic	2.590	2.057	0.945	7.099	0.187
zero Season - 2	2.538	1.655	1.445	4.459	0.064
zero Season - 3	1.052	1.438	0.575	1.926	0.889
zero Season - 4	1.298	1.477	0.700	2.407	0.504
zero Age - 45-54 yrs	-				
zero Age - 55-64 yrs	-				
zero Age - 65+ yrs	-				
zero Sex - male	8.463	3.612	5.008	14.301	0.096
zero Social grade - C1C2	<0.001	8.563	<0.001	Inf	<0.001
zero Social grade - AB	5.700	4.479	3.234	10.047	0.246
zero - Number of adults	0.359	2.161	0.279	0.462	0.184
zero Presence of children - Yes	-				
zero Region - North of England	-				
zero Festival - Valentine's Day	-				
zero Festival - Easter	-				
zeroFestival - Halloween	-				
zero Festival - Christmas	-				
zero Interaction Time*Pandemic	0.796	1.110	0.684	0.926	0.028
zero Interaction Pandemic*Presence of Children - Yes	-				
Observations	16,806				

Model coefficients from secondary analysis; interactions with age group of the main reporter

HFSS = high in fat, salt and sugar; UPF = ultra-processed food; OOH = out-of-home. Due to multicollinearity, the variables region, presence of children, and age of the main shopper were not included in the OOH models

Outcome	Total Energy purchased					Energy purchased from fruit & vegetables				
Term	Exp. estimate	SE	95%CI low	95%CI high	p value	Exp. estimate	SE	95%CI low	95%CI high	p value
count Constant	14953.260	1.047	14600.740	15314.292	<0.001	0.082	1.089	0.080	0.085	<0.001
count Time	1.001	1.000	1.000	1.001	<0.001	0.999	1.000	0.998	0.999	<0.001
count Pandemic - during pandemic	1.277	1.020	1.235	1.321	<0.001	0.899	1.036	0.859	0.941	0.003
count Season - 2	1.006	1.006	0.993	1.020	0.268	1.028	1.012	1.009	1.048	0.023
count Season - 3	0.969	1.007	0.956	0.983	<0.001	1.025	1.013	1.006	1.045	0.053
count Season - 4	1.070	1.007	1.054	1.086	<0.001	0.894	1.013	0.876	0.913	<0.001
count Age - 45-54 yrs	1.151	1.031	1.134	1.168	<0.001	0.916	1.052	0.897	0.935	0.084
count Age - 55-64 yrs	1.275	1.033	1.254	1.295	<0.001	0.859	1.061	0.840	0.879	0.011
count Age - 65+ yrs	1.308	1.033	1.287	1.330	<0.001	0.861	1.063	0.841	0.880	0.013
count Sex - male	0.971	1.023	0.961	0.981	0.194	0.971	1.038	0.958	0.985	0.435
count Social grade - C1C2	1.002	1.029	0.990	1.014	0.947	1.144	1.045	1.125	1.164	0.002
count Social grade - AB	0.928	1.034	0.914	0.941	0.026	1.422	1.053	1.393	1.451	<0.001
count - Number of adults	0.864	1.012	0.859	0.868	<0.001	0.890	1.021	0.883	0.896	<0.001
count Presence of children - Yes	0.807	1.026	0.796	0.817	<0.001	0.826	1.046	0.812	0.841	<0.001
count Region - North of England	1.042	1.020	1.033	1.052	0.040	0.757	1.035	0.747	0.767	<0.001
count Festival - Valentine's Day	0.998	1.012	0.969	1.027	0.838	0.926	1.020	0.890	0.963	<0.001
count Festival - Easter	1.042	1.013	1.011	1.074	0.001	0.983	1.025	0.944	1.025	0.491
countFestival - Halloween	0.922	1.017	0.884	0.961	<0.001	1.067	1.038	1.008	1.131	0.084
count Festival - Christmas	0.856	1.025	0.820	0.893	<0.001	1.077	1.047	1.014	1.144	0.104
count Interaction Time*Pandemic	0.999	1.001	0.996	1.002	0.383	1.000	1.003	0.996	1.005	0.916
count Interaction Pandemic*Age - 45-54 yrs	0.982	1.025	0.949	1.017	0.481	0.971	1.040	0.926	1.017	0.446
count Interaction Pandemic*Age - 55-64 yrs	0.918	1.025	0.887	0.950	0.001	1.016	1.040	0.970	1.065	0.683
count Interaction Pandemic*Age - 65+ yrs	0.843	1.027	0.814	0.872	<0.001	1.045	1.040	0.997	1.095	0.268
zero Constant	0.078	1.176	0.067	0.092	<0.001	0.477	1.199	0.427	0.534	<0.001
zero Time	0.998	1.001	0.996	1.001	0.147	1.000	1.001	0.999	1.002	0.891
zero Pandemic - during pandemic	1.449	1.107	1.187	1.769	<0.001	0.785	1.098	0.659	0.934	0.010
zero Season - 2	1.060	1.045	0.964	1.165	0.185	0.977	1.033	0.913	1.046	0.482
zero Season - 3	1.412	1.041	1.290	1.546	<0.001	1.040	1.037	0.973	1.113	0.279
zero Season - 4	1.120	1.053	1.005	1.247	0.029	1.182	1.037	1.100	1.271	<0.001
zero Age - 45-54 yrs	0.755	1.101	0.689	0.828	0.004	0.838	1.121	0.781	0.900	0.123
zero Age - 55-64 yrs	0.564	1.124	0.508	0.626	<0.001	0.685	1.142	0.635	0.738	0.004
zero Age - 65+ yrs	0.433	1.129	0.388	0.484	<0.001	0.455	1.143	0.420	0.493	<0.001
zero Sex - male	0.871	1.083	0.812	0.934	0.085	1.336	1.094	1.273	1.401	0.001
zero Social grade - C1C2	0.979	1.106	0.900	1.065	0.835	0.631	1.111	0.597	0.667	<0.001
zero Social grade - AB	1.108	1.121	1.005	1.222	0.368	0.550	1.143	0.512	0.591	<0.001
zero - Number of adults	0.922	1.044	0.889	0.957	0.058	0.689	1.066	0.669	0.710	<0.001
zero Presence of children - Yes	0.866	1.092	0.799	0.939	0.101	0.661	1.116	0.619	0.706	<0.001
zero Region - North of England	0.995	1.069	0.936	1.058	0.944	1.107	1.086	1.057	1.158	0.221
zero Festival - Valentine's Day	0.937	1.114	0.758	1.160	0.548	1.100	1.061	0.959	1.260	0.112
zero Festival - Easter	1.252	1.098	1.043	1.502	0.016	0.964	1.080	0.823	1.129	0.635
zeroFestival - Halloween	1.642	1.122	1.298	2.077	<0.001	0.879	1.099	0.718	1.076	0.173
zero Festival - Christmas	2.543	1.103	2.073	3.119	<0.001	1.326	1.093	1.106	1.591	0.002
zero Interaction Time*Pandemic	0.979	1.010	0.958	0.999	0.029	0.987	1.009	0.970	1.004	0.112
zero Interaction Pandemic*Age - 45-54 yrs	0.870	1.119	0.706	1.072	0.217	0.957	1.120	0.794	1.152	0.694
zero Interaction Pandemic*Age - 55-64 yrs	0.859	1.127	0.687	1.073	0.202	1.099	1.123	0.919	1.316	0.414
zero Interaction Pandemic*Age - 65+ yrs	1.414	1.129	1.142	1.751	0.004	1.292	1.119	1.072	1.557	0.023
Observations	89,382					89,382				

Outcome	Energy purchased from HFSS					Energy purchased from UPF				
Term	Exp. estimate	SE	95%CI low	95%CI high	p value	Exp. estimate	SE	95%CI low	95%CI high	p value
count Constant	0.518	1.021	0.511	0.525	<0.001	0.602	1.026	0.593	0.610	<0.001
count Time	1.000	1.000	1.000	1.000	0.779	1.000	1.000	1.000	1.000	0.764
count Pandemic - during pandemic	1.025	1.009	1.006	1.046	0.005	0.973	1.009	0.955	0.992	0.004
count Season - 2	1.033	1.003	1.025	1.041	<0.001	1.017	1.003	1.009	1.025	<0.001
count Season - 3	1.039	1.004	1.031	1.048	<0.001	1.024	1.003	1.016	1.032	<0.001
count Season - 4	1.073	1.004	1.064	1.083	<0.001	1.026	1.003	1.017	1.036	<0.001
count Age - 45-54 yrs	0.997	1.014	0.989	1.006	0.844	1.019	1.018	1.010	1.028	0.284
count Age - 55-64 yrs	0.996	1.016	0.986	1.005	0.791	0.996	1.020	0.986	1.005	0.823
count Age - 65+ yrs	1.003	1.016	0.994	1.013	0.835	0.987	1.020	0.978	0.997	0.516
count Sex - male	1.006	1.011	1.000	1.012	0.546	0.998	1.013	0.992	1.004	0.886
count Social grade - C1C2	0.971	1.012	0.964	0.978	0.017	0.955	1.015	0.948	0.962	0.002
count Social grade - AB	0.941	1.015	0.932	0.949	<0.001	0.900	1.018	0.893	0.908	<0.001
count - Number of adults	0.992	1.006	0.989	0.995	0.158	0.992	1.007	0.989	0.995	0.211
count Presence of children - Yes	1.015	1.012	1.007	1.023	0.214	1.055	1.015	1.047	1.063	<0.001
count Region - North of England	1.015	1.009	1.010	1.021	0.096	1.059	1.012	1.054	1.065	<0.001
count Festival - Valentine's Day	1.022	1.007	1.005	1.040	0.001	1.014	1.006	0.997	1.031	0.016
count Festival - Easter	0.982	1.007	0.965	1.000	0.011	0.999	1.006	0.982	1.017	0.914
countFestival - Halloween	0.994	1.010	0.970	1.019	0.568	1.012	1.008	0.987	1.036	0.171
count Festival - Christmas	0.994	1.012	0.969	1.019	0.595	0.991	1.011	0.967	1.017	0.416
count Interaction Time*Pandemic	1.000	1.001	0.998	1.002	0.762	1.000	1.001	0.998	1.002	0.750
count Interaction Pandemic*Age - 45-54 yrs	1.006	1.011	0.986	1.027	0.558	1.002	1.011	0.982	1.022	0.882
count Interaction Pandemic*Age - 55-64 yrs	0.986	1.010	0.966	1.006	0.158	0.994	1.011	0.974	1.014	0.558
count Interaction Pandemic*Age - 65+ yrs	0.965	1.011	0.946	0.985	0.001	0.989	1.011	0.970	1.009	0.333
zero Constant	0.103	1.241	0.083	0.128	<0.001	0.026	1.323	0.018	0.036	<0.001
zero Time	0.998	1.001	0.995	1.001	0.227	0.997	1.002	0.992	1.001	0.154
zero Pandemic - during pandemic	1.141	1.182	0.839	1.551	0.429	1.365	1.268	0.878	2.122	0.190
zero Season - 2	0.966	1.065	0.850	1.098	0.581	1.014	1.108	0.836	1.230	0.891
zero Season - 3	0.968	1.067	0.849	1.104	0.615	1.165	1.101	0.960	1.412	0.114
zero Season - 4	0.814	1.084	0.697	0.950	0.010	0.892	1.117	0.705	1.129	0.305
zero Age - 45-54 yrs	0.630	1.159	0.547	0.725	0.002	0.706	1.256	0.577	0.864	0.127
zero Age - 55-64 yrs	0.505	1.176	0.436	0.586	<0.001	0.473	1.246	0.380	0.590	0.001
zero Age - 65+ yrs	0.525	1.179	0.455	0.606	<0.001	0.518	1.246	0.420	0.639	0.003
zero Sex - male	1.228	1.115	1.119	1.348	0.060	1.215	1.156	1.061	1.392	0.179
zero Social grade - C1C2	1.059	1.145	0.935	1.200	0.669	1.299	1.190	1.063	1.589	0.132
zero Social grade - AB	1.338	1.166	1.161	1.541	0.058	1.853	1.217	1.489	2.306	0.002
zero - Number of adults	0.663	1.074	0.625	0.703	<0.001	0.788	1.089	0.727	0.855	0.005
zero Presence of children - Yes	0.454	1.147	0.396	0.522	<0.001	0.345	1.195	0.279	0.426	<0.001
zero Region - North of England	0.690	1.103	0.632	0.754	<0.001	0.600	1.131	0.526	0.683	<0.001
zero Festival - Valentine's Day	0.819	1.155	0.610	1.099	0.166	0.986	1.237	0.647	1.500	0.946
zero Festival - Easter	0.976	1.149	0.734	1.298	0.861	1.131	1.216	0.760	1.682	0.530
zeroFestival - Halloween	0.992	1.250	0.638	1.543	0.971	1.025	1.393	0.534	1.966	0.941
zero Festival - Christmas	2.002	1.197	1.426	2.809	<0.001	3.124	1.247	2.048	4.765	<0.001
zero Interaction Time*Pandemic	0.962	1.016	0.933	0.993	0.016	0.996	1.025	0.954	1.040	0.877
zero Interaction Pandemic*Age - 45-54 yrs	0.891	1.249	0.625	1.270	0.603	0.595	1.344	0.361	0.981	0.079
zero Interaction Pandemic*Age - 55-64 yrs	1.195	1.234	0.855	1.671	0.396	1.159	1.309	0.733	1.832	0.584
zero Interaction Pandemic*Age - 65+ yrs	1.469	1.228	1.077	2.003	0.062	1.122	1.300	0.724	1.739	0.661
Observations	89,382					89,382				

Outcome	Energy purchased from savoury snacks					Enegy purchased from chocolate & confectionery				
Term	Exp. estimate	SE	95%CI low	95%CI high	p value	Exp. estimate	SE	95%CI low	95%CI high	p value
count Constant	0.138	1.078	0.133	0.144	<0.001	0.151	1.064	0.145	0.157	<0.001
count Time	1.000	1.000	1.000	1.001	0.176	1.001	1.000	1.000	1.001	0.021
count Pandemic - during pandemic	0.930	1.033	0.885	0.978	0.024	0.949	1.036	0.899	1.002	0.134
count Season - 2	1.012	1.013	0.991	1.034	0.330	1.039	1.014	1.015	1.063	0.005
count Season - 3	1.065	1.013	1.042	1.089	<0.001	1.046	1.016	1.022	1.072	0.003
count Season - 4	1.079	1.015	1.053	1.105	<0.001	1.174	1.016	1.144	1.204	<0.001
count Age - 45-54 yrs	0.923	1.046	0.902	0.944	0.074	0.993	1.046	0.968	1.018	0.870
count Age - 55-64 yrs	0.816	1.054	0.796	0.837	<0.001	0.932	1.045	0.907	0.958	0.108
count Age - 65+ yrs	0.725	1.061	0.706	0.744	<0.001	0.867	1.047	0.843	0.892	0.002
count Sex - male	1.132	1.037	1.114	1.151	0.001	1.040	1.032	1.022	1.059	0.215
count Social grade - C1C2	0.905	1.041	0.888	0.923	0.014	0.899	1.038	0.880	0.917	0.004
count Social grade - AB	0.921	1.046	0.900	0.943	0.067	0.976	1.045	0.952	1.000	0.576
count - Number of adults	0.898	1.017	0.891	0.905	<0.001	0.872	1.018	0.864	0.880	<0.001
count Presence of children - Yes	0.804	1.043	0.789	0.820	<0.001	0.876	1.039	0.857	0.895	0.001
count Region - North of England	0.880	1.029	0.867	0.893	<0.001	0.984	1.028	0.969	0.999	0.556
count Festival - Valentine's Day	1.078	1.026	1.031	1.127	0.004	1.043	1.030	0.994	1.094	0.154
count Festival - Easter	0.968	1.026	0.924	1.015	0.203	1.192	1.030	1.137	1.249	<0.001
countFestival - Halloween	1.006	1.041	0.942	1.075	0.881	1.123	1.040	1.051	1.201	0.003
count Festival - Christmas	1.243	1.043	1.160	1.332	<0.001	1.168	1.057	1.081	1.263	0.005
count Interaction Time*Pandemic	1.000	1.003	0.995	1.005	0.949	0.988	1.003	0.983	0.994	<0.001
count Interaction Pandemic*Age - 45-54 yrs	0.979	1.037	0.931	1.030	0.559	1.024	1.041	0.969	1.083	0.548
count Interaction Pandemic*Age - 55-64 yrs	0.981	1.040	0.932	1.033	0.624	1.028	1.040	0.972	1.086	0.492
count Interaction Pandemic*Age - 65+ yrs	1.071	1.041	1.013	1.132	0.085	1.017	1.046	0.960	1.078	0.702
zero Constant	1.742	1.133	1.620	1.872	<0.001	1.670	1.124	1.554	1.794	<0.001
zero Time	0.999	1.001	0.998	1.000	0.146	0.999	1.001	0.998	1.000	0.069
zero Pandemic - during pandemic	0.866	1.057	0.782	0.959	0.009	0.685	1.059	0.619	0.759	<0.001
zero Season - 2	0.915	1.020	0.878	0.954	<0.001	0.902	1.021	0.865	0.939	<0.001
zero Season - 3	0.968	1.022	0.928	1.009	0.128	0.939	1.022	0.900	0.978	0.003
zero Season - 4	0.871	1.024	0.832	0.912	<0.001	0.713	1.024	0.681	0.746	<0.001
zero Age - 45-54 yrs	0.885	1.087	0.845	0.926	0.142	0.844	1.083	0.807	0.884	0.035
zero Age - 55-64 yrs	0.892	1.095	0.850	0.937	0.210	0.706	1.092	0.672	0.741	<0.001
zero Age - 65+ yrs	1.173	1.098	1.116	1.232	0.087	0.765	1.093	0.728	0.804	0.003
zero Sex - male	1.012	1.067	0.981	1.043	0.858	1.287	1.063	1.248	1.326	<0.001
zero Social grade - C1C2	0.907	1.081	0.874	0.941	0.210	1.057	1.075	1.018	1.097	0.447
zero Social grade - AB	1.037	1.094	0.992	1.084	0.690	1.250	1.089	1.196	1.306	0.009
zero - Number of adults	0.805	1.034	0.791	0.818	<0.001	0.888	1.033	0.873	0.902	<0.001
zero Presence of children - Yes	0.595	1.080	0.572	0.619	<0.001	0.676	1.075	0.650	0.702	<0.001
zero Region - North of England	0.980	1.057	0.953	1.007	0.712	0.757	1.056	0.737	0.778	<0.001
zero Festival - Valentine's Day	0.871	1.041	0.798	0.950	0.001	0.798	1.040	0.732	0.870	<0.001
zero Festival - Easter	1.021	1.043	0.932	1.119	0.616	0.695	1.045	0.632	0.763	<0.001
zeroFestival - Halloween	1.097	1.059	0.967	1.245	0.105	0.949	1.060	0.835	1.078	0.371
zero Festival - Christmas	1.226	1.064	1.078	1.396	0.001	1.698	1.067	1.493	1.932	<0.001
zero Interaction Time*Pandemic	0.990	1.005	0.980	0.999	0.026	1.009	1.005	0.999	1.019	0.059
zero Interaction Pandemic*Age - 45-54 yrs	0.980	1.068	0.880	1.091	0.759	0.990	1.071	0.891	1.101	0.888
zero Interaction Pandemic*Age - 55-64 yrs	1.219	1.070	1.098	1.354	0.003	1.143	1.072	1.029	1.269	0.056
zero Interaction Pandemic*Age - 65+ yrs	1.279	1.068	1.151	1.422	<0.001	1.378	1.074	1.241	1.530	<0.001
Observations	89,382					89,382				

Outcome	Energy purchased from low-sugar soft drinks					Energy from medium-sugar soft drinks				
Term	Exp. estimate	SE	95%CI low	95%CI high	p value	Exp. estimate	SE	95%CI low	95%CI high	p value
count Constant	0.021	1.203	0.019	0.022	<0.001	0.038	1.253	0.030	0.048	<0.001
count Time	0.999	1.001	0.998	1.000	0.421	1.004	1.003	1.001	1.007	0.089
count Pandemic - during pandemic	0.838	1.089	0.753	0.932	0.037	0.700	1.314	0.490	1.001	0.192
count Season - 2	1.006	1.046	0.961	1.054	0.890	0.895	1.088	0.783	1.023	0.190
count Season - 3	1.121	1.045	1.070	1.174	0.009	0.948	1.102	0.830	1.085	0.587
count Season - 4	1.001	1.042	0.950	1.055	0.980	0.899	1.124	0.781	1.035	0.364
count Age - 45-54 yrs	0.725	1.122	0.691	0.761	0.005	1.129	1.163	0.975	1.307	0.421
count Age - 55-64 yrs	0.873	1.159	0.828	0.920	0.356	1.218	1.167	1.038	1.428	0.202
count Age - 65+ yrs	0.651	1.145	0.615	0.689	0.002	0.978	1.181	0.834	1.147	0.892
count Sex - male	1.107	1.095	1.068	1.146	0.262	1.082	1.103	0.978	1.197	0.419
count Social grade - C1C2	0.744	1.124	0.714	0.776	0.011	0.671	1.214	0.591	0.761	0.040
count Social grade - AB	0.621	1.135	0.590	0.653	<0.001	0.709	1.212	0.611	0.823	0.075
count - Number of adults	0.909	1.047	0.896	0.923	0.037	0.846	1.063	0.806	0.887	0.006
count Presence of children - Yes	0.725	1.098	0.697	0.754	0.001	1.077	1.163	0.946	1.227	0.623
count Region - North of England	0.876	1.096	0.849	0.905	0.149	0.948	1.100	0.868	1.036	0.577
count Festival - Valentine's Day	1.080	1.078	0.978	1.194	0.306	1.408	1.226	1.062	1.866	0.094
count Festival - Easter	1.107	1.104	1.003	1.221	0.306	0.751	1.131	0.558	1.011	0.020
countFestival - Halloween	0.983	1.139	0.852	1.134	0.894	1.256	1.330	0.816	1.933	0.425
count Festival - Christmas	1.796	1.240	1.533	2.103	0.006	0.655	1.226	0.463	0.926	0.038
count Interaction Time*Pandemic	1.009	1.007	0.999	1.019	0.208	1.035	1.026	1.005	1.067	0.178
count Interaction Pandemic*Age - 45-54 yrs	1.096	1.104	0.987	1.217	0.354	1.476	1.357	1.056	2.061	0.202
count Interaction Pandemic*Age - 55-64 yrs	0.917	1.116	0.823	1.021	0.428	0.776	1.272	0.562	1.071	0.291
count Interaction Pandemic*Age - 65+ yrs	1.204	1.133	1.070	1.354	0.136	0.879	1.290	0.612	1.262	0.613
zero Constant	3.413	1.175	3.152	3.696	<0.001	49.624	1.326	38.930	63.257	<0.001
zero Time	1.000	1.001	0.999	1.001	0.797	0.996	1.002	0.993	0.999	0.068
zero Pandemic - during pandemic	0.894	1.061	0.800	0.998	0.059	1.623	1.231	1.130	2.332	0.020
zero Season - 2	0.869	1.022	0.830	0.909	<0.001	0.799	1.076	0.694	0.921	0.002
zero Season - 3	0.804	1.023	0.768	0.841	<0.001	0.852	1.086	0.740	0.981	0.051
zero Season - 4	0.946	1.024	0.900	0.995	0.019	0.737	1.082	0.638	0.853	<0.001
zero Age - 45-54 yrs	0.885	1.114	0.842	0.930	0.258	0.859	1.185	0.738	0.999	0.370
zero Age - 55-64 yrs	1.010	1.125	0.958	1.065	0.931	0.907	1.236	0.771	1.067	0.645
zero Age - 65+ yrs	1.254	1.125	1.188	1.324	0.055	0.970	1.224	0.821	1.147	0.882
zero Sex - male	1.183	1.084	1.144	1.224	0.037	1.288	1.152	1.159	1.431	0.074
zero Social grade - C1C2	0.956	1.104	0.918	0.996	0.649	0.823	1.197	0.723	0.936	0.277
zero Social grade - AB	1.343	1.123	1.279	1.411	0.011	0.844	1.213	0.725	0.982	0.380
zero - Number of adults	0.758	1.046	0.744	0.772	<0.001	0.943	1.076	0.896	0.992	0.423
zero Presence of children - Yes	0.653	1.103	0.626	0.682	<0.001	1.194	1.156	1.048	1.360	0.221
zero Region - North of England	0.559	1.074	0.542	0.576	<0.001	1.242	1.143	1.135	1.360	0.105
zero Festival - Valentine's Day	1.035	1.041	0.942	1.139	0.390	0.911	1.158	0.679	1.222	0.524
zero Festival - Easter	0.952	1.044	0.862	1.052	0.259	1.087	1.156	0.794	1.489	0.564
zeroFestival - Halloween	0.996	1.063	0.867	1.144	0.944	1.515	1.238	0.960	2.388	0.051
zero Festival - Christmas	1.215	1.069	1.054	1.401	0.004	0.918	1.203	0.635	1.327	0.642
zero Interaction Time*Pandemic	0.983	1.005	0.972	0.993	<0.001	0.968	1.016	0.938	0.999	0.040
zero Interaction Pandemic*Age - 45-54 yrs	0.922	1.076	0.821	1.034	0.262	0.870	1.254	0.613	1.234	0.537
zero Interaction Pandemic*Age - 55-64 yrs	1.063	1.075	0.949	1.190	0.403	0.698	1.271	0.497	0.980	0.133
zero Interaction Pandemic*Age - 65+ yrs	1.186	1.079	1.057	1.331	0.024	1.095	1.284	0.752	1.595	0.717
Observations	89,382					89,382				

Term	Outcome: Energy from high-sugar soft drinks — Exp. estimate	SE	95%CI low	95%CI high	p value	Alcohol volume — Exp. estimate	SE	95%CI low	95%CI high	p value
count Constant	0.104	1.175	0.091	0.118	<0.001	3637.614	1.174	3400.521	3891.238	<0.001
count Time	0.999	1.001	0.997	1.001	0.388	1.001	1.001	1.000	1.002	0.055
count Pandemic - during pandemic	0.687	1.125	0.574	0.821	0.001	1.224	1.054	1.126	1.331	<0.001
count Season - 2	0.928	1.056	0.858	1.005	0.172	1.098	1.022	1.060	1.137	<0.001
count Season - 3	1.146	1.073	1.056	1.244	0.052	1.093	1.027	1.055	1.133	0.001
count Season - 4	0.935	1.067	0.854	1.024	0.303	1.125	1.024	1.082	1.169	<0.001
count Age - 45-54 yrs	0.749	1.127	0.693	0.810	0.016	1.156	1.101	1.108	1.207	0.131
count Age - 55-64 yrs	0.595	1.148	0.548	0.646	<0.001	1.080	1.114	1.033	1.129	0.478
count Age - 65+ yrs	0.630	1.214	0.570	0.696	0.017	0.939	1.135	0.897	0.982	0.618
count Sex - male	1.155	1.104	1.086	1.228	0.145	1.112	1.075	1.083	1.141	0.144
count Social grade - C1C2	0.831	1.115	0.777	0.888	0.088	0.791	1.117	0.766	0.817	0.034
count Social grade - AB	0.794	1.115	0.729	0.865	0.034	0.722	1.121	0.695	0.750	0.004
count - Number of adults	0.856	1.055	0.834	0.878	0.004	0.695	1.038	0.686	0.705	<0.001
count Presence of children - Yes	0.694	1.090	0.649	0.742	<0.001	0.885	1.091	0.853	0.917	0.161
count Region - North of England	0.851	1.096	0.806	0.900	0.080	1.374	1.070	1.341	1.408	<0.001
count Festival - Valentine's Day	0.841	1.089	0.708	1.000	0.042	0.911	1.038	0.846	0.980	0.012
count Festival - Easter	0.946	1.086	0.814	1.099	0.500	1.083	1.040	1.008	1.165	0.041
countFestival - Halloween	0.831	1.117	0.651	1.060	0.092	0.910	1.058	0.820	1.010	0.094
count Festival - Christmas	0.837	1.123	0.674	1.039	0.123	1.038	1.064	0.935	1.152	0.546
count Interaction Time*Pandemic	1.017	1.011	0.999	1.036	0.122	0.995	1.005	0.988	1.002	0.301
count Interaction Pandemic*Age - 45-54 yrs	1.031	1.178	0.865	1.229	0.850	1.015	1.078	0.934	1.103	0.842
count Interaction Pandemic*Age - 55-64 yrs	1.398	1.183	1.168	1.673	0.046	0.913	1.063	0.841	0.992	0.138
count Interaction Pandemic*Age - 65+ yrs	1.382	1.218	1.113	1.715	0.101	0.895	1.067	0.821	0.976	0.088
zero Constant	10.515	1.335	9.076	12.182	<0.001	8.612	1.190	7.939	9.342	<0.001
zero Time	1.001	1.001	0.999	1.003	0.304	0.999	1.001	0.998	1.000	0.323
zero Pandemic - during pandemic	0.942	1.110	0.772	1.148	0.564	0.736	1.066	0.660	0.820	<0.001
zero Season - 2	0.844	1.048	0.773	0.922	<0.001	0.829	1.022	0.792	0.867	<0.001
zero Season - 3	0.891	1.048	0.813	0.977	0.014	0.816	1.024	0.779	0.854	<0.001
zero Season - 4	0.844	1.054	0.763	0.933	0.001	0.686	1.025	0.653	0.721	<0.001
zero Age - 45-54 yrs	1.683	1.207	1.538	1.840	0.006	0.776	1.129	0.737	0.817	0.036
zero Age - 55-64 yrs	1.733	1.253	1.577	1.906	0.015	0.655	1.138	0.621	0.691	0.001
zero Age - 65+ yrs	3.107	1.278	2.775	3.478	<0.001	0.680	1.141	0.644	0.718	0.003
zero Sex - male	0.889	1.157	0.832	0.949	0.417	1.011	1.092	0.979	1.045	0.900
zero Social grade - C1C2	1.562	1.185	1.453	1.680	0.008	0.782	1.119	0.751	0.815	0.029
zero Social grade - AB	1.992	1.213	1.814	2.189	<0.001	0.801	1.139	0.763	0.840	0.087
zero - Number of adults	0.810	1.078	0.785	0.835	0.005	0.843	1.048	0.828	0.857	<0.001
zero Presence of children - Yes	1.238	1.197	1.147	1.338	0.234	1.338	1.115	1.282	1.397	0.007
zero Region - North of England	1.364	1.141	1.286	1.447	0.018	0.612	1.083	0.594	0.631	<0.001
zero Festival - Valentine's Day	0.963	1.087	0.794	1.168	0.650	0.820	1.039	0.746	0.901	<0.001
zero Festival - Easter	0.715	1.078	0.603	0.847	0.000	0.976	1.039	0.887	1.074	0.527
zeroFestival - Halloween	1.052	1.125	0.801	1.382	0.665	1.198	1.056	1.048	1.370	0.001
zero Festival - Christmas	0.738	1.126	0.578	0.944	0.011	1.199	1.068	1.048	1.371	0.006
zero Interaction Time*Pandemic	0.988	1.010	0.968	1.008	0.225	0.986	1.004	0.976	0.996	0.001
zero Interaction Pandemic*Age - 45-54 yrs	1.027	1.143	0.843	1.251	0.842	0.921	1.081	0.826	1.027	0.288
zero Interaction Pandemic*Age - 55-64 yrs	1.209	1.131	0.988	1.479	0.124	1.176	1.080	1.055	1.310	0.035
zero Interaction Pandemic*Age - 65+ yrs	1.048	1.215	0.824	1.332	0.809	1.413	1.084	1.265	1.579	<0.001
Observations	89,382					89,382				

Outcome			OOH purchasing		
Term	Exp. estimate	SE	95%CI low	95%CI high	p value
count Constant	1.353	1.350	1.229	1.489	0.314
count Time	0.999	1.001	0.997	1.000	0.036
count Pandemic - during pandemic	0.535	1.109	0.468	0.613	<0.001
count Season - 2	0.961	1.026	0.912	1.012	0.112
count Season - 3	0.994	1.024	0.945	1.046	0.798
count Season - 4	0.994	1.024	0.939	1.052	0.800
count Age - 45-54 yrs	0.910	1.178	0.866	0.957	0.566
count Age - 55-64 yrs	1.066	1.192	1.009	1.126	0.718
count Age - 65+ yrs	0.761	1.271	0.705	0.821	0.255
count Sex - male	1.502	1.137	1.445	1.562	0.002
count Social grade - C1C2	1.030	1.217	0.977	1.085	0.881
count Social grade - AB	1.031	1.298	0.963	1.105	0.905
count - Number of adults	1.006	1.078	0.984	1.028	0.938
count Presence of children - Yes	0.995	1.172	0.950	1.042	0.975
count Region - North of England	1.107	1.125	1.068	1.148	0.385
count Festival - Valentine's Day	1.005	1.027	0.905	1.116	0.837
count Festival - Easter	0.872	1.042	0.770	0.987	0.001
countFestival - Halloween	1.013	1.041	0.872	1.175	0.754
count Festival - Christmas	0.728	1.060	0.617	0.859	<0.001
count Interaction Time*Pandemic	1.020	1.008	1.006	1.034	0.016
count Interaction Pandemic*Age - 45-54 yrs	1.022	1.150	0.897	1.164	0.877
count Interaction Pandemic*Age - 55-64 yrs	0.857	1.160	0.750	0.980	0.301
count Interaction Pandemic*Age - 65+ yrs	0.545	1.283	0.432	0.687	0.015
zero Constant	0.066	4.370	0.027	0.158	0.065
zero Time	1.008	1.013	0.993	1.023	0.560
zero Pandemic - during pandemic	2.227	2.037	0.756	6.561	0.260
zero Season - 2	2.457	1.665	1.394	4.331	0.078
zero Season - 3	1.043	1.436	0.572	1.902	0.908
zero Season - 4	1.271	1.485	0.684	2.362	0.545
zero Age - 45-54 yrs	-				
zero Age - 55-64 yrs	-				
zero Age - 65+ yrs	-				
zero Sex - male	8.933	3.812	5.169	15.439	0.102
zero Social grade - C1C2	<0.001	5.711	<0.001	189660.934	<0.001
zero Social grade - AB	6.203	4.626	3.418	11.257	0.233
zero - Number of adults	0.358	2.178	0.277	0.461	0.187
zero Presence of children - Yes	-				
zero Region - North of England	-				
zero Festival - Valentine's Day	-				
zero Festival - Easter	-				
zeroFestival - Halloween	-				
zero Festival - Christmas	-				
zero Interaction Time*Pandemic	0.782	1.127	0.656	0.931	0.040
zero Interaction Pandemic*Age - 45-54 yrs	-				
zero Interaction Pandemic*Age - 55-64 yrs	-				
zero Interaction Pandemic*Age - 65+ yrs	-				
Observations	16,806				

Model coefficients from secondary analysis; interactions with social grade of the main reporter

HFSS = high in fat, salt and sugar; UPF = ultra-processed food; OOH = out-of-home.

Due to multicollineraity, the variables region, presence of children, and age of the main shopper were not included in the OOH models

Outcome	Total Energy purchased					Energy purchased from fruit & vegetables				
Term	Exp. estimate	SE	95%CI low	95%CI high	p value	Exp. estimate	SE	95%CI low	95%CI high	p value
count Constant	15341.460	1.047	14977.837	15713.910	<0.001	0.081	1.089	0.079	0.084	<0.001
count Time	1.001	1.000	1.000	1.001	<0.001	0.999	1.000	0.998	0.999	<0.001
count Pandemic - during pandemic	1.095	1.021	1.055	1.135	<0.001	0.966	1.045	0.919	1.016	0.432
count Season - 2	1.007	1.006	0.993	1.021	0.214	1.028	1.012	1.009	1.048	0.023
count Season - 3	0.970	1.007	0.956	0.983	<0.001	1.025	1.013	1.006	1.044	0.054
count Season - 4	1.070	1.007	1.054	1.087	<0.001	0.894	1.013	0.875	0.913	<0.001
count Age - 45-54 yrs	1.147	1.029	1.132	1.163	<0.001	0.911	1.051	0.894	0.928	0.059
count Age - 55-64 yrs	1.256	1.032	1.237	1.275	<0.001	0.862	1.060	0.844	0.880	0.011
count Age - 65+ yrs	1.271	1.032	1.252	1.291	<0.001	0.867	1.061	0.849	0.886	0.017
count Sex - male	0.971	1.023	0.961	0.981	0.195	0.971	1.038	0.958	0.985	0.436
count Social grade - C1C2	0.986	1.030	0.973	1.000	0.641	1.164	1.046	1.143	1.186	0.001
count Social grade - AB	0.909	1.036	0.894	0.924	0.007	1.426	1.055	1.395	1.458	<0.001
count - Number of adults	0.864	1.012	0.859	0.868	<0.001	0.890	1.021	0.883	0.896	<0.001
count Presence of children - Yes	0.806	1.026	0.796	0.817	<0.001	0.827	1.046	0.812	0.841	<0.001
count Region - North of England	1.042	1.020	1.033	1.052	0.040	0.757	1.035	0.748	0.767	<0.001
count Festival - Valentine's Day	0.998	1.012	0.970	1.027	0.875	0.926	1.020	0.890	0.963	<0.001
count Festival - Easter	1.042	1.013	1.011	1.074	0.001	0.983	1.025	0.944	1.025	0.490
countFestival - Halloween	0.921	1.017	0.884	0.961	<0.001	1.067	1.038	1.007	1.131	0.085
count Festival - Christmas	0.855	1.025	0.819	0.893	<0.001	1.077	1.047	1.014	1.144	0.103
count Interaction Time*Pandemic	0.999	1.001	0.996	1.002	0.323	1.000	1.003	0.996	1.005	0.913
count Interaction Pandemic*Social grade - C1C2	1.103	1.023	1.067	1.140	<0.001	0.905	1.042	0.865	0.947	0.015
count Interaction Pandemic*Social grade - AB	1.133	1.029	1.090	1.178	<0.001	0.982	1.047	0.931	1.035	0.688
zero Constant	0.076	1.180	0.065	0.090	<0.001	0.458	1.200	0.409	0.513	<0.001
zero Time	0.998	1.001	0.996	1.001	0.146	1.000	1.001	0.999	1.002	0.893
zero Pandemic - during pandemic	1.665	1.131	1.316	2.108	<0.001	1.045	1.097	0.880	1.242	0.632
zero Season - 2	1.060	1.045	0.964	1.165	0.184	0.977	1.033	0.913	1.046	0.480
zero Season - 3	1.412	1.041	1.290	1.545	<0.001	1.040	1.037	0.973	1.113	0.280
zero Season - 4	1.120	1.053	1.005	1.247	0.029	1.182	1.037	1.100	1.270	<0.001
zero Age - 45-54 yrs	0.737	1.098	0.677	0.803	0.001	0.833	1.120	0.779	0.891	0.107
zero Age - 55-64 yrs	0.549	1.122	0.499	0.605	<0.001	0.693	1.139	0.646	0.744	0.005
zero Age - 65+ yrs	0.466	1.123	0.422	0.516	<0.001	0.472	1.141	0.438	0.509	<0.001
zero Sex - male	0.871	1.083	0.812	0.934	0.085	1.336	1.094	1.273	1.402	0.001
zero Social grade - C1C2	1.005	1.112	0.915	1.104	0.964	0.656	1.114	0.618	0.696	<0.001
zero Social grade - AB	1.159	1.127	1.040	1.292	0.217	0.578	1.145	0.535	0.624	<0.001
zero - Number of adults	0.922	1.044	0.889	0.956	0.057	0.689	1.066	0.669	0.710	<0.001
zero Presence of children - Yes	0.866	1.092	0.799	0.939	0.102	0.662	1.116	0.620	0.707	<0.001
zero Region - North of England	0.995	1.069	0.936	1.058	0.944	1.107	1.086	1.058	1.159	0.219
zero Festival - Valentine's Day	0.937	1.114	0.758	1.159	0.546	1.099	1.061	0.959	1.260	0.112
zero Festival - Easter	1.251	1.098	1.043	1.501	0.016	0.964	1.080	0.823	1.130	0.637
zeroFestival - Halloween	1.642	1.122	1.298	2.077	<0.001	0.879	1.099	0.719	1.076	0.174
zero Festival - Christmas	2.543	1.103	2.073	3.119	<0.001	1.326	1.093	1.106	1.590	0.002
zero Interaction Time*Pandemic	0.979	1.010	0.959	0.999	0.029	0.987	1.009	0.970	1.004	0.116
zero Interaction Pandemic*Social grade - C1C2	0.876	1.118	0.710	1.081	0.235	0.765	1.097	0.656	0.892	0.004
zero Interaction Pandemic*Social grade - AB	0.786	1.145	0.614	1.007	0.075	0.705	1.139	0.574	0.866	0.007
Observations	89,382					89,382				

Outcome	Energy purchased from HFSS					Energy purchased from UPF				
Term	Exp. estimate	SE	95%CI low	95%CI high	p value	Exp. estimate	SE	95%CI low	95%CI high	p value
count Constant	0.519	1.021	0.512	0.527	<0.001	0.601	1.026	0.593	0.609	<0.001
count Time	1.000	1.000	1.000	1.000	0.776	1.000	1.000	1.000	1.000	0.761
count Pandemic - during pandemic	1.010	1.010	0.989	1.032	0.309	0.979	1.011	0.958	1.000	0.045
count Season - 2	1.033	1.003	1.025	1.042	<0.001	1.017	1.003	1.009	1.025	<0.001
count Season - 3	1.039	1.004	1.031	1.048	<0.001	1.024	1.003	1.016	1.032	<0.001
count Season - 4	1.073	1.004	1.064	1.083	<0.001	1.026	1.003	1.017	1.036	<0.001
count Age - 45-54 yrs	0.998	1.013	0.990	1.007	0.902	1.019	1.017	1.011	1.027	0.267
count Age - 55-64 yrs	0.993	1.015	0.985	1.002	0.664	0.995	1.020	0.986	1.003	0.777
count Age - 65+ yrs	0.997	1.016	0.988	1.006	0.863	0.985	1.020	0.977	0.994	0.454
count Sex - male	1.006	1.011	1.000	1.013	0.545	0.998	1.013	0.992	1.004	0.887
count Social grade - C1C2	0.970	1.013	0.962	0.978	0.017	0.956	1.015	0.949	0.964	0.003
count Social grade - AB	0.941	1.016	0.932	0.950	<0.001	0.904	1.019	0.896	0.913	<0.001
count - Number of adults	0.992	1.006	0.989	0.995	0.159	0.992	1.007	0.989	0.995	0.212
count Presence of children - Yes	1.015	1.012	1.007	1.023	0.215	1.055	1.015	1.047	1.063	<0.001
count Region - North of England	1.015	1.009	1.010	1.021	0.095	1.059	1.012	1.054	1.065	<0.001
count Festival - Valentine's Day	1.022	1.007	1.005	1.040	0.001	1.014	1.006	0.998	1.031	0.016
count Festival - Easter	0.982	1.007	0.965	1.000	0.011	0.999	1.006	0.982	1.017	0.916
countFestival - Halloween	0.994	1.010	0.970	1.019	0.573	1.012	1.008	0.987	1.036	0.169
count Festival - Christmas	0.994	1.012	0.969	1.019	0.589	0.991	1.011	0.967	1.017	0.416
count Interaction Time*Pandemic	1.000	1.001	0.998	1.002	0.712	1.000	1.001	0.998	1.002	0.740
count Interaction Pandemic*Social grade - C1C2	1.007	1.010	0.988	1.027	0.478	0.993	1.011	0.975	1.013	0.538
count Interaction Pandemic*Social grade - AB	0.999	1.013	0.976	1.022	0.943	0.974	1.013	0.953	0.997	0.047
zero Constant	0.096	1.243	0.077	0.121	<0.001	0.023	1.331	0.016	0.033	<0.001
zero Time	0.998	1.001	0.995	1.001	0.226	0.997	1.002	0.992	1.001	0.154
zero Pandemic - during pandemic	1.656	1.206	1.178	2.328	0.007	2.083	1.312	1.243	3.492	0.007
zero Season - 2	0.966	1.065	0.850	1.097	0.580	1.014	1.108	0.836	1.230	0.890
zero Season - 3	0.968	1.067	0.848	1.104	0.610	1.164	1.101	0.960	1.411	0.116
zero Season - 4	0.814	1.084	0.697	0.950	0.010	0.893	1.117	0.705	1.130	0.306
zero Age - 45-54 yrs	0.620	1.153	0.543	0.706	0.001	0.651	1.237	0.541	0.784	0.044
zero Age - 55-64 yrs	0.520	1.170	0.453	0.597	<0.001	0.487	1.227	0.399	0.595	<0.001
zero Age - 65+ yrs	0.562	1.167	0.493	0.641	<0.001	0.530	1.226	0.438	0.642	0.002
zero Sex - male	1.228	1.115	1.119	1.348	0.060	1.216	1.156	1.062	1.393	0.178
zero Social grade - C1C2	1.117	1.154	0.972	1.283	0.439	1.479	1.212	1.174	1.863	0.042
zero Social grade - AB	1.407	1.177	1.203	1.645	0.037	2.027	1.242	1.578	2.604	0.001
zero - Number of adults	0.663	1.074	0.624	0.703	<0.001	0.788	1.089	0.727	0.854	0.005
zero Presence of children - Yes	0.455	1.147	0.396	0.522	<0.001	0.345	1.195	0.280	0.426	<0.001
zero Region - North of England	0.690	1.103	0.632	0.754	<0.001	0.600	1.131	0.526	0.684	<0.001
zero Festival - Valentine's Day	0.818	1.155	0.610	1.098	0.164	0.985	1.237	0.647	1.500	0.944
zero Festival - Easter	0.976	1.149	0.734	1.298	0.862	1.131	1.216	0.761	1.682	0.529
zeroFestival - Halloween	0.992	1.250	0.638	1.543	0.972	1.027	1.393	0.535	1.969	0.937
zero Festival - Christmas	2.001	1.197	1.426	2.808	<0.001	3.122	1.247	2.046	4.763	<0.001
zero Interaction Time*Pandemic	0.962	1.016	0.933	0.993	0.016	0.996	1.025	0.954	1.040	0.878
zero Interaction Pandemic*Social grade - C1C2	0.745	1.201	0.543	1.020	0.107	0.537	1.291	0.335	0.860	0.015
zero Interaction Pandemic*Social grade - AB	0.756	1.243	0.528	1.084	0.200	0.668	1.332	0.402	1.111	0.160
Observations	89,382					89,382				

Outcome	Energy purchased from savoury snacks					Enegy purchased from chocolate & confectionery				
Term	Exp. estimate	SE	95%CI low	95%CI high	p value	Exp. estimate	SE	95%CI low	95%CI high	p value
count Constant	0.139	1.078	0.133	0.144	<0.001	0.149	1.065	0.143	0.155	<0.001
count Time	1.000	1.000	1.000	1.001	0.175	1.001	1.000	1.000	1.001	0.020
count Pandemic - during pandemic	0.926	1.042	0.873	0.981	0.061	1.026	1.044	0.966	1.090	0.544
count Season - 2	1.012	1.013	0.991	1.034	0.335	1.039	1.014	1.015	1.064	0.005
count Season - 3	1.065	1.013	1.042	1.089	<0.001	1.047	1.016	1.022	1.072	0.003
count Season - 4	1.079	1.015	1.053	1.105	<0.001	1.174	1.016	1.144	1.204	<0.001
count Age - 45-54 yrs	0.919	1.044	0.900	0.938	0.050	0.996	1.045	0.973	1.020	0.933
count Age - 55-64 yrs	0.813	1.052	0.795	0.832	<0.001	0.936	1.044	0.913	0.960	0.124
count Age - 65+ yrs	0.734	1.060	0.716	0.752	<0.001	0.869	1.046	0.847	0.892	0.002
count Sex - male	1.132	1.037	1.114	1.151	0.001	1.040	1.032	1.022	1.059	0.216
count Social grade - C1C2	0.904	1.043	0.884	0.923	0.015	0.908	1.039	0.888	0.929	0.013
count Social grade - AB	0.922	1.048	0.899	0.947	0.085	0.996	1.047	0.969	1.023	0.922
count - Number of adults	0.898	1.017	0.891	0.905	<0.001	0.872	1.018	0.864	0.880	<0.001
count Presence of children - Yes	0.804	1.043	0.789	0.820	<0.001	0.876	1.039	0.857	0.895	0.001
count Region - North of England	0.880	1.029	0.867	0.892	<0.001	0.984	1.028	0.969	1.000	0.561
count Festival - Valentine's Day	1.078	1.026	1.031	1.128	0.004	1.043	1.030	0.994	1.094	0.151
count Festival - Easter	0.969	1.026	0.924	1.016	0.213	1.191	1.030	1.136	1.249	<0.001
countFestival - Halloween	1.006	1.041	0.942	1.075	0.878	1.124	1.040	1.051	1.202	0.003
count Festival - Christmas	1.243	1.043	1.160	1.332	<0.001	1.169	1.057	1.081	1.263	0.005
count Interaction Time*Pandemic	1.000	1.003	0.995	1.005	0.998	0.988	1.003	0.983	0.994	<0.001
count Interaction Pandemic*Social grade - C1C2	1.013	1.041	0.962	1.067	0.747	0.940	1.044	0.891	0.991	0.151
count Interaction Pandemic*Social grade - AB	0.994	1.046	0.935	1.057	0.895	0.893	1.049	0.838	0.952	0.018
zero Constant	1.676	1.134	1.559	1.803	<0.001	1.593	1.124	1.482	1.712	<0.001
zero Time	0.999	1.001	0.998	1.000	0.145	0.999	1.001	0.998	1.000	0.068
zero Pandemic - during pandemic	1.087	1.065	0.973	1.213	0.187	0.910	1.070	0.815	1.016	0.166
zero Season - 2	0.915	1.020	0.878	0.954	<0.001	0.902	1.021	0.865	0.939	<0.001
zero Season - 3	0.967	1.022	0.928	1.009	0.125	0.938	1.022	0.900	0.978	0.003
zero Season - 4	0.871	1.024	0.831	0.912	<0.001	0.712	1.024	0.680	0.746	<0.001
zero Age - 45-54 yrs	0.882	1.086	0.845	0.920	0.127	0.843	1.082	0.809	0.879	0.031
zero Age - 55-64 yrs	0.922	1.094	0.881	0.965	0.366	0.722	1.090	0.689	0.755	<0.001
zero Age - 65+ yrs	1.223	1.096	1.167	1.280	0.029	0.807	1.090	0.771	0.846	0.013
zero Sex - male	1.011	1.067	0.981	1.043	0.860	1.287	1.063	1.248	1.326	<0.001
zero Social grade - C1C2	0.926	1.083	0.889	0.965	0.337	1.093	1.078	1.049	1.138	0.236
zero Social grade - AB	1.070	1.097	1.020	1.124	0.460	1.295	1.092	1.233	1.359	0.003
zero - Number of adults	0.805	1.034	0.791	0.818	<0.001	0.888	1.033	0.873	0.902	<0.001
zero Presence of children - Yes	0.595	1.080	0.572	0.619	<0.001	0.676	1.075	0.650	0.703	<0.001
zero Region - North of England	0.980	1.057	0.953	1.008	0.716	0.757	1.056	0.737	0.779	<0.001
zero Festival - Valentine's Day	0.870	1.041	0.798	0.949	0.001	0.797	1.040	0.731	0.869	<0.001
zero Festival - Easter	1.021	1.043	0.932	1.119	0.618	0.695	1.045	0.633	0.764	<0.001
zeroFestival - Halloween	1.097	1.059	0.967	1.245	0.105	0.949	1.061	0.835	1.078	0.372
zero Festival - Christmas	1.227	1.064	1.078	1.396	0.001	1.699	1.067	1.493	1.933	<0.001
zero Interaction Time*Pandemic	0.990	1.005	0.980	1.000	0.030	1.009	1.005	0.999	1.019	0.054
zero Interaction Pandemic*Social grade - C1C2	0.883	1.065	0.799	0.975	0.048	0.816	1.071	0.739	0.901	0.003
zero Interaction Pandemic*Social grade - AB	0.825	1.078	0.733	0.927	0.010	0.806	1.085	0.717	0.906	0.008
Observations	89,382					89,382				

Outcome	Energy purchased from low-sugar soft drinks					Energy from medium-sugar soft drinks				
Term	Exp. estimate	SE	95%CI low	95%CI high	p value	Exp. estimate	SE	95%CI low	95%CI high	p value
count Constant	0.020	1.204	0.018	0.022	<0.001	0.040	1.275	0.032	0.051	<0.001
count Time	0.999	1.001	0.998	1.000	0.409	1.004	1.002	1.001	1.007	0.079
count Pandemic - during pandemic	1.003	1.136	0.888	1.133	0.982	0.617	1.412	0.428	0.891	0.162
count Season - 2	1.005	1.046	0.960	1.053	0.910	0.889	1.087	0.777	1.017	0.159
count Season - 3	1.120	1.045	1.069	1.173	0.010	0.949	1.103	0.830	1.085	0.593
count Season - 4	1.001	1.042	0.950	1.055	0.977	0.909	1.127	0.789	1.046	0.422
count Age - 45-54 yrs	0.738	1.115	0.706	0.771	0.005	1.235	1.186	1.080	1.412	0.215
count Age - 55-64 yrs	0.860	1.149	0.819	0.903	0.276	1.173	1.159	1.010	1.363	0.279
count Age - 65+ yrs	0.672	1.140	0.638	0.708	0.002	0.967	1.178	0.833	1.123	0.838
count Sex - male	1.106	1.095	1.068	1.145	0.266	1.089	1.105	0.983	1.206	0.392
count Social grade - C1C2	0.767	1.128	0.733	0.803	0.028	0.621	1.257	0.540	0.715	0.037
count Social grade - AB	0.635	1.139	0.600	0.672	<0.001	0.695	1.261	0.589	0.821	0.117
count - Number of adults	0.908	1.047	0.895	0.922	0.036	0.836	1.060	0.796	0.877	0.002
count Presence of children - Yes	0.724	1.098	0.696	0.753	0.001	1.122	1.178	0.985	1.277	0.484
count Region - North of England	0.877	1.096	0.849	0.906	0.153	0.941	1.101	0.861	1.028	0.524
count Festival - Valentine's Day	1.079	1.078	0.976	1.192	0.313	1.440	1.233	1.086	1.910	0.082
count Festival - Easter	1.106	1.103	1.002	1.220	0.302	0.804	1.133	0.597	1.083	0.082
countFestival - Halloween	0.983	1.138	0.852	1.133	0.891	1.228	1.302	0.797	1.892	0.437
count Festival - Christmas	1.789	1.239	1.527	2.095	0.007	0.630	1.230	0.445	0.892	0.026
count Interaction Time*Pandemic	1.009	1.007	0.999	1.019	0.212	1.031	1.029	1.000	1.062	0.293
count Interaction Pandemic*Social grade - C1C2	0.835	1.140	0.750	0.930	0.170	1.337	1.363	0.961	1.861	0.348
count Interaction Pandemic*Social grade - AB	0.888	1.155	0.780	1.012	0.410	0.940	1.382	0.647	1.365	0.847
zero Constant	3.308	1.175	3.053	3.583	<0.001	50.687	1.327	39.683	64.743	<0.001
zero Time	1.000	1.001	0.999	1.001	0.791	0.996	1.002	0.993	0.999	0.069
zero Pandemic - during pandemic	1.076	1.074	0.954	1.214	0.304	1.432	1.255	0.971	2.113	0.114
zero Season - 2	0.868	1.022	0.830	0.908	<0.001	0.800	1.076	0.694	0.921	0.002
zero Season - 3	0.803	1.023	0.767	0.841	<0.001	0.852	1.086	0.740	0.981	0.052
zero Season - 4	0.946	1.024	0.900	0.995	0.018	0.737	1.082	0.638	0.853	<0.001
zero Age - 45-54 yrs	0.873	1.113	0.834	0.914	0.205	0.839	1.176	0.730	0.964	0.280
zero Age - 55-64 yrs	1.021	1.124	0.972	1.073	0.860	0.849	1.222	0.730	0.986	0.413
zero Age - 65+ yrs	1.291	1.124	1.228	1.359	0.029	0.984	1.219	0.841	1.151	0.935
zero Sex - male	1.183	1.084	1.144	1.224	0.037	1.289	1.152	1.160	1.432	0.073
zero Social grade - C1C2	0.987	1.105	0.944	1.031	0.893	0.817	1.206	0.709	0.941	0.279
zero Social grade - AB	1.378	1.125	1.306	1.454	0.007	0.865	1.225	0.731	1.022	0.474
zero - Number of adults	0.758	1.046	0.744	0.772	<0.001	0.943	1.076	0.896	0.992	0.422
zero Presence of children - Yes	0.654	1.103	0.627	0.682	<0.001	1.194	1.156	1.048	1.360	0.221
zero Region - North of England	0.559	1.074	0.542	0.576	<0.001	1.242	1.143	1.134	1.359	0.106
zero Festival - Valentine's Day	1.035	1.041	0.941	1.138	0.393	0.911	1.158	0.679	1.222	0.524
zero Festival - Easter	0.952	1.044	0.862	1.052	0.258	1.087	1.156	0.794	1.489	0.565
zeroFestival - Halloween	0.996	1.063	0.867	1.144	0.945	1.516	1.238	0.961	2.390	0.051
zero Festival - Christmas	1.215	1.069	1.054	1.401	0.004	0.918	1.203	0.634	1.327	0.642
zero Interaction Time*Pandemic	0.983	1.005	0.973	0.993	<0.001	0.968	1.016	0.938	0.999	0.040
zero Interaction Pandemic*Social grade - C1C2	0.826	1.076	0.741	0.920	0.009	1.041	1.238	0.743	1.460	0.849
zero Interaction Pandemic*Social grade - AB	0.857	1.087	0.754	0.975	0.064	0.880	1.278	0.597	1.298	0.603
Observations	89,382					89,382				

Outcome	Energy from high-sugar soft drinks					Alcohol volume				
Term	Exp. estimate	SE	95%CI low	95%CI high	p value	Exp. estimate	SE	95%CI low	95%CI high	p value
count Constant	0.112	1.170	0.099	0.127	<0.001	3755.540	1.173	3510.209	4018.018	<0.001
count Time	0.999	1.001	0.997	1.001	0.368	1.001	1.001	1.000	1.002	0.055
count Pandemic - during pandemic	0.307	2.145	0.086	1.105	0.122	1.032	1.063	0.940	1.133	0.606
count Season - 2	0.927	1.056	0.856	1.003	0.166	1.098	1.022	1.060	1.137	<0.001
count Season - 3	1.145	1.074	1.055	1.243	0.056	1.093	1.027	1.055	1.133	0.001
count Season - 4	0.934	1.068	0.853	1.022	0.297	1.125	1.024	1.082	1.170	<0.001
count Age - 45-54 yrs	0.751	1.114	0.699	0.806	0.008	1.161	1.096	1.117	1.207	0.104
count Age - 55-64 yrs	0.629	1.141	0.582	0.679	<0.001	1.061	1.110	1.018	1.105	0.571
count Age - 65+ yrs	0.666	1.197	0.608	0.731	0.024	0.920	1.130	0.882	0.959	0.494
count Sex - male	1.153	1.104	1.084	1.226	0.151	1.111	1.075	1.083	1.140	0.145
count Social grade - C1C2	0.813	1.121	0.756	0.874	0.071	0.773	1.119	0.745	0.801	0.022
count Social grade - AB	0.774	1.122	0.705	0.850	0.026	0.700	1.125	0.671	0.731	0.002
count - Number of adults	0.853	1.055	0.832	0.876	0.003	0.695	1.038	0.686	0.704	<0.001
count Presence of children - Yes	0.689	1.091	0.644	0.737	<0.001	0.884	1.091	0.853	0.917	0.159
count Region - North of England	0.851	1.097	0.806	0.900	0.081	1.374	1.070	1.341	1.408	<0.001
count Festival - Valentine's Day	0.840	1.088	0.707	0.999	0.040	0.911	1.038	0.847	0.981	0.013
count Festival - Easter	0.941	1.092	0.810	1.094	0.491	1.084	1.040	1.008	1.165	0.042
countFestival - Halloween	0.835	1.119	0.654	1.065	0.108	0.912	1.058	0.821	1.012	0.100
count Festival - Christmas	0.837	1.124	0.674	1.040	0.129	1.037	1.064	0.934	1.151	0.558
count Interaction Time*Pandemic	1.014	1.011	0.995	1.032	0.233	0.995	1.005	0.987	1.002	0.275
count Interaction Pandemic*Social grade - C1C2	1.141	1.171	0.959	1.357	0.403	1.148	1.064	1.057	1.246	0.027
count Interaction Pandemic*Social grade - AB	1.183	1.210	0.949	1.473	0.378	1.188	1.081	1.079	1.308	0.027
zero Constant	9.221	1.337	8.004	10.623	<0.001	8.208	1.190	7.566	8.904	<0.001
zero Time	1.001	1.001	0.999	1.003	0.304	0.999	1.001	0.998	1.000	0.320
zero Pandemic - during pandemic	2.624	2.012	0.640	10.763	0.168	0.947	1.073	0.841	1.067	0.442
zero Season - 2	0.844	1.048	0.772	0.922	<0.001	0.829	1.022	0.793	0.867	<0.001
zero Season - 3	0.891	1.048	0.813	0.976	0.014	0.816	1.024	0.779	0.854	<0.001
zero Season - 4	0.843	1.054	0.763	0.932	0.001	0.687	1.025	0.653	0.721	<0.001
zero Age - 45-54 yrs	1.690	1.201	1.558	1.835	0.004	0.764	1.125	0.730	0.800	0.022
zero Age - 55-64 yrs	1.792	1.249	1.641	1.957	0.009	0.675	1.136	0.643	0.709	0.002
zero Age - 65+ yrs	3.134	1.268	2.824	3.477	<0.001	0.725	1.138	0.689	0.762	0.013
zero Sex - male	0.889	1.157	0.832	0.949	0.417	1.011	1.092	0.978	1.045	0.902
zero Social grade - C1C2	1.633	1.190	1.508	1.767	0.005	0.811	1.121	0.775	0.848	0.066
zero Social grade - AB	2.077	1.218	1.873	2.302	<0.001	0.817	1.141	0.774	0.862	0.126
zero - Number of adults	0.810	1.078	0.785	0.835	0.005	0.843	1.048	0.828	0.857	<0.001
zero Presence of children - Yes	1.239	1.197	1.147	1.338	0.234	1.337	1.115	1.281	1.396	0.007
zero Region - North of England	1.364	1.141	1.286	1.447	0.018	0.612	1.083	0.594	0.631	<0.001
zero Festival - Valentine's Day	0.963	1.087	0.794	1.168	0.649	0.820	1.039	0.747	0.901	<0.001
zero Festival - Easter	0.716	1.078	0.604	0.848	<0.001	0.975	1.039	0.887	1.073	0.515
zeroFestival - Halloween	1.052	1.125	0.801	1.382	0.665	1.198	1.056	1.048	1.370	0.001
zero Festival - Christmas	0.738	1.126	0.578	0.944	0.011	1.198	1.068	1.048	1.371	0.006
zero Interaction Time*Pandemic	0.988	1.010	0.968	1.008	0.230	0.986	1.004	0.976	0.996	0.001
zero Interaction Pandemic*Social grade - C1C2	0.771	1.147	0.634	0.937	0.058	0.824	1.076	0.741	0.916	0.008
zero Interaction Pandemic*Social grade - AB	0.782	1.174	0.612	1.000	0.125	0.895	1.097	0.791	1.014	0.235
Observations	89,382					89,382				

Outcome	OOH purchasing				
Term	Exp. estimate	SE	95%CI low	95%CI high	p value
count Constant	1.347	1.356	1.223	1.483	0.329
count Time	0.999	1.001	0.998	1.000	0.047
count Pandemic - during pandemic	0.704	1.235	0.583	0.849	0.096
count Season - 2	0.972	1.026	0.922	1.024	0.270
count Season - 3	0.998	1.024	0.949	1.050	0.948
count Season - 4	0.997	1.024	0.942	1.055	0.896
count Age - 45-54 yrs	0.912	1.180	0.871	0.956	0.579
count Age - 55-64 yrs	1.047	1.190	0.994	1.103	0.793
count Age - 65+ yrs	0.721	1.262	0.671	0.775	0.160
count Sex - male	1.512	1.137	1.454	1.572	0.001
count Social grade - C1C2	1.057	1.218	1.000	1.116	0.780
count Social grade - AB	1.066	1.292	0.992	1.144	0.805
count - Number of adults	1.000	1.078	0.978	1.023	1.000
count Presence of children - Yes	0.996	1.173	0.951	1.043	0.979
count Region - North of England	1.106	1.125	1.067	1.147	0.390
count Festival - Valentine's Day	1.007	1.027	0.907	1.118	0.791
count Festival - Easter	0.886	1.045	0.781	1.004	0.006
countFestival - Halloween	1.013	1.041	0.873	1.175	0.747
count Festival - Christmas	0.726	1.060	0.616	0.857	<0.001
count Interaction Time*Pandemic	1.013	1.011	0.998	1.028	0.224
count Interaction Pandemic*Social grade - C1C2	0.731	1.223	0.618	0.866	0.120
count Interaction Pandemic*Social grade - AB	0.731	1.260	0.594	0.899	0.174
zero Constant	0.046	4.430	0.017	0.122	0.039
zero Time	1.012	1.013	0.996	1.028	0.355
zero Pandemic - during pandemic	24.583	3.168	8.128	74.345	0.005
zero Season - 2	3.303	1.721	1.859	5.867	0.028
zero Season - 3	1.200	1.460	0.647	2.227	0.630
zero Season - 4	1.384	1.506	0.741	2.585	0.428
zero Age - 45-54 yrs	-				
zero Age - 55-64 yrs	-				
zero Age - 65+ yrs	-				
zero Sex - male	7.203	3.884	4.369	11.874	0.146
zero Social grade - C1C2	<0.001	5.204	<0.001	5,527,284,996.23	<0.001
zero Social grade - AB	10.657	4.511	4.973	22.838	0.116
zero - Number of adults	0.367	1.957	0.289	0.466	0.136
zero Presence of children - Yes	-				
zero Region - North of England	-				
zero Festival - Valentine's Day	-				
zero Festival - Easter	-				
zeroFestival - Halloween	-				
zero Festival - Christmas	-				
zero Interaction Time*Pandemic	0.794	1.089	0.710	0.888	0.007
zero Interaction Pandemic*Social grade - C1C2	492.040	5.873	<0.001	1.62744E+16	<0.001
zero Interaction Pandemic*Social grade - AB	0.046	3.824	0.014	0.155	0.022
Observations	16,806				

Moedel coefficients from secondary analysis; interactions with usual purchasing (purchasing levels before the pandemic)
HFSS = high in fat, salt and sugar; UPF = ultra-processed food; OOH = out-of-home; PPP = pre-pandemic purchasing
Due to multicollineraity, the variables region, presence of children, and age of the main shopper were not included in the OOH models
PPP was mainly modelled as quartiles; due to lower purchasing levels, no quartiles could be built for some outcomes, so that pre-pandemic alcohol purchasing was categorised into tertiles, while OOH purchasing were split along the median. Because the more than half of observations for pre-pandemic medium and high-sugar soft drink purchasing were 0, PPP was determined for these variables for considering the highest quartile as 'high' and the remainder as 'low'

Outcome	Total Energy purchased					Energy purchased from fruit & vegetables				
Term	Exp. estimate	SE	95%CI low	95%CI high	p value	Exp. estimate	SE	95%CI low	95%CI high	p value
count Constant	8405.253	1.035	8207.298	8607.983	<0.001	0.039	1.057	0.038	0.040	<0.001
count Time	1.001	1.000	1.001	1.001	<0.001	0.999	1.000	0.999	1.000	0.022
count Pandemic - during pandemic	1.423	1.020	1.379	1.468	<0.001	1.127	1.042	1.081	1.175	0.004
count Season - 2	0.997	1.006	0.984	1.010	0.603	0.981	1.011	0.965	0.998	0.087
count Season - 3	0.967	1.006	0.955	0.980	<0.001	1.028	1.013	1.010	1.045	0.033
count Season - 4	1.069	1.007	1.054	1.085	<0.001	0.901	1.014	0.884	0.918	<0.001
count Age - 45-54 yrs	1.039	1.019	1.026	1.053	0.039	0.955	1.031	0.939	0.972	0.137
count Age - 55-64 yrs	1.040	1.021	1.025	1.054	0.063	0.895	1.037	0.878	0.912	0.002
count Age - 65+ yrs	1.012	1.022	0.997	1.027	0.579	0.903	1.038	0.886	0.921	0.006
count Sex - male	0.990	1.015	0.980	0.999	0.469	1.006	1.023	0.993	1.018	0.804
count Social grade - C1C2	1.001	1.018	0.990	1.013	0.940	1.017	1.026	1.002	1.033	0.505
count Social grade - AB	1.012	1.022	0.999	1.026	0.564	1.139	1.034	1.118	1.160	<0.001
count - Number of adults	0.936	1.007	0.931	0.941	<0.001	0.928	1.013	0.922	0.934	<0.001
count Presence of children - Yes	0.926	1.018	0.915	0.938	<0.001	0.889	1.028	0.875	0.903	<0.001
count Region - North of England	1.006	1.012	0.998	1.015	0.595	0.899	1.021	0.889	0.910	<0.001
count Festival - Valentine's Day	0.998	1.012	0.972	1.025	0.885	0.930	1.019	0.897	0.964	<0.001
count Festival - Easter	1.037	1.013	1.009	1.067	0.004	0.984	1.022	0.948	1.021	0.447
countFestival - Halloween	0.921	1.017	0.886	0.958	<0.001	1.074	1.038	1.019	1.132	0.053
count Festival - Christmas	0.860	1.025	0.827	0.895	<0.001	1.064	1.039	1.008	1.124	0.105
count PPP - 2	1.420	1.019	1.401	1.439	<0.001	1.372	1.029	1.347	1.398	<0.001
count PPP - 3	1.774	1.019	1.749	1.798	<0.001	1.902	1.029	1.868	1.938	<0.001
count PPP - 4	2.542	1.023	2.505	2.580	<0.001	3.259	1.035	3.199	3.321	<0.001
count Interaction Time*Pandemic	0.999	1.001	0.996	1.002	0.516	1.003	1.003	1.000	1.007	0.230
count Interaction Pandemic*PPP - 2	0.880	1.024	0.852	0.909	<0.001	0.879	1.046	0.842	0.918	0.004
count Interaction Pandemic*PPP - 3	0.826	1.023	0.800	0.853	<0.001	0.783	1.040	0.750	0.818	<0.001
count Interaction Pandemic*PPP - 4	0.747	1.026	0.724	0.771	<0.001	0.743	1.040	0.712	0.776	<0.001
zero Constant	0.136	1.193	0.115	0.161	<0.001	0.980	1.173	0.873	1.100	0.899
zero Time	0.999	1.001	0.996	1.001	0.157	1.000	1.001	0.999	1.002	0.884
zero Pandemic - during pandemic	1.195	1.106	0.975	1.464	0.077	0.712	1.090	0.611	0.830	<0.001
zero Season - 2	1.059	1.045	0.964	1.165	0.189	0.982	1.035	0.916	1.053	0.603
zero Season - 3	1.417	1.042	1.294	1.551	<0.001	1.040	1.039	0.970	1.114	0.309
zero Season - 4	1.123	1.053	1.008	1.251	0.026	1.188	1.039	1.103	1.280	<0.001
zero Age - 45-54 yrs	0.807	1.094	0.740	0.879	0.017	0.801	1.104	0.748	0.857	0.025
zero Age - 55-64 yrs	0.661	1.119	0.599	0.730	<0.001	0.685	1.118	0.637	0.737	0.001
zero Age - 65+ yrs	0.600	1.122	0.541	0.666	<0.001	0.539	1.123	0.499	0.582	<0.001
zero Sex - male	0.852	1.081	0.794	0.915	0.041	1.198	1.082	1.140	1.259	0.022
zero Social grade - C1C2	0.985	1.104	0.905	1.073	0.882	0.800	1.102	0.755	0.847	0.022
zero Social grade - AB	1.007	1.119	0.913	1.112	0.948	0.852	1.129	0.791	0.918	0.189
zero - Number of adults	0.850	1.045	0.818	0.882	<0.001	0.717	1.057	0.696	0.738	<0.001
zero Presence of children - Yes	0.755	1.092	0.695	0.820	0.001	0.686	1.102	0.642	0.733	<0.001
zero Region - North of England	1.039	1.068	0.977	1.105	0.562	0.906	1.077	0.864	0.950	0.185
zero Festival - Valentine's Day	0.937	1.114	0.757	1.160	0.549	1.110	1.065	0.965	1.277	0.097
zero Festival - Easter	1.252	1.098	1.044	1.503	0.016	0.961	1.082	0.819	1.129	0.619

zeroFestival - Halloween	1.646	1.123	1.300	2.084	<0.001	0.868	1.105	0.706	1.068	0.158
zero Festival - Christmas	2.562	1.104	2.087	3.146	<0.001	1.381	1.099	1.145	1.665	0.001
zero PPP - 2	0.695	1.087	0.637	0.757	<0.001	0.316	1.100	0.296	0.337	<0.001
zero PPP - 3	0.481	1.101	0.436	0.532	<0.001	0.237	1.103	0.220	0.254	<0.001
zero PPP - 4	0.368	1.127	0.329	0.411	<0.001	0.173	1.110	0.160	0.187	<0.001
zero Interaction Time*Pandemic	0.978	1.010	0.958	0.999	0.026	0.985	1.009	0.968	1.003	0.090
zero Interaction Pandemic*PPP - 2	1.152	1.119	0.937	1.416	0.210	1.377	1.119	1.161	1.632	0.004
zero Interaction Pandemic*PPP - 3	1.409	1.122	1.134	1.752	0.003	1.346	1.128	1.116	1.623	0.014
zero Interaction Pandemic*PPP - 4	1.627	1.133	1.299	2.036	<0.001	1.395	1.122	1.147	1.695	0.004
Observations	89,382					89,382				

Outcome	Energy purchased from HFSS					Energy purchased from UPF				
Term	Exp. estimate	SE	95%CI low	95%CI high	p value	Exp. estimate	SE	95%CI low	95%CI high	p value
count Constant	0.437	1.017	0.431	0.443	<0.001	0.461	1.019	0.455	0.468	<0.001
count Time	1.000	1.000	1.000	1.000	0.414	1.000	1.000	1.000	1.000	0.368
count Pandemic - during pandemic	1.053	1.011	1.033	1.073	<0.001	1.004	1.011	0.987	1.022	0.697
count Season - 2	1.029	1.003	1.021	1.037	<0.001	1.012	1.003	1.005	1.020	<0.001
count Season - 3	1.039	1.004	1.031	1.048	<0.001	1.024	1.003	1.017	1.032	<0.001
count Season - 4	1.075	1.004	1.066	1.084	<0.001	1.029	1.004	1.020	1.037	<0.001
count Age - 45-54 yrs	0.986	1.009	0.978	0.993	0.122	1.007	1.012	1.000	1.015	0.516
count Age - 55-64 yrs	0.979	1.011	0.971	0.987	0.056	0.984	1.013	0.976	0.992	0.210
count Age - 65+ yrs	0.970	1.011	0.962	0.979	0.005	0.979	1.013	0.971	0.987	0.096
count Sex - male	1.003	1.007	0.997	1.009	0.690	0.989	1.008	0.984	0.994	0.181
count Social grade - C1C2	0.980	1.009	0.973	0.987	0.019	0.989	1.009	0.982	0.995	0.213
count Social grade - AB	0.965	1.011	0.957	0.973	0.001	0.973	1.012	0.966	0.981	0.019
count - Number of adults	0.995	1.004	0.992	0.998	0.191	0.992	1.004	0.989	0.995	0.064
count Presence of children - Yes	1.002	1.009	0.994	1.009	0.858	1.016	1.011	1.010	1.023	0.119
count Region - North of England	0.998	1.006	0.993	1.003	0.750	1.012	1.007	1.007	1.017	0.120
count Festival - Valentine's Day	1.024	1.007	1.007	1.040	0.001	1.015	1.006	1.000	1.031	0.015
count Festival - Easter	0.980	1.007	0.964	0.997	0.006	1.001	1.007	0.985	1.017	0.892
countFestival - Halloween	0.993	1.010	0.970	1.016	0.466	1.012	1.009	0.990	1.035	0.185
count Festival - Christmas	0.994	1.012	0.971	1.019	0.642	0.992	1.011	0.969	1.015	0.449
count PPP - 2	1.136	1.011	1.126	1.145	<0.001	1.239	1.013	1.230	1.249	<0.001
count PPP - 3	1.246	1.010	1.236	1.257	<0.001	1.394	1.013	1.383	1.404	<0.001
count PPP - 4	1.405	1.011	1.393	1.416	<0.001	1.606	1.013	1.593	1.618	<0.001
count Interaction Time*Pandemic	1.000	1.001	0.998	1.002	0.886	1.000	1.001	0.998	1.002	0.965
count Interaction Pandemic*PPP - 2	0.984	1.012	0.965	1.003	0.181	0.976	1.013	0.958	0.994	0.058
count Interaction Pandemic*PPP - 3	0.981	1.011	0.962	1.000	0.086	0.962	1.012	0.945	0.980	0.002
count Interaction Pandemic*PPP - 4	0.915	1.012	0.897	0.933	<0.001	0.946	1.012	0.928	0.963	<0.001
zero Constant	0.203	1.243	0.162	0.255	<0.001	0.052	1.319	0.037	0.074	<0.001
zero Time	0.998	1.002	0.995	1.001	0.239	0.997	1.002	0.992	1.001	0.156
zero Pandemic - during pandemic	0.886	1.156	0.671	1.169	0.404	1.007	1.229	0.676	1.500	0.974
zero Season - 2	0.971	1.066	0.853	1.104	0.642	1.020	1.109	0.840	1.237	0.850
zero Season - 3	0.969	1.068	0.849	1.107	0.634	1.168	1.102	0.962	1.417	0.109
zero Season - 4	0.815	1.084	0.698	0.953	0.012	0.891	1.118	0.704	1.128	0.301
zero Age - 45-54 yrs	0.676	1.138	0.592	0.772	0.003	0.696	1.221	0.578	0.838	0.070
zero Age - 55-64 yrs	0.581	1.150	0.505	0.667	<0.001	0.534	1.216	0.437	0.653	0.001
zero Age - 65+ yrs	0.630	1.151	0.551	0.720	0.001	0.567	1.210	0.468	0.687	0.003
zero Sex - male	1.177	1.102	1.072	1.293	0.094	1.227	1.148	1.071	1.406	0.137
zero Social grade - C1C2	1.036	1.137	0.914	1.174	0.783	1.166	1.174	0.953	1.425	0.341
zero Social grade - AB	1.174	1.158	1.017	1.355	0.275	1.424	1.205	1.142	1.777	0.058

zero - Number of adults	0.661	1.069	0.622	0.702	<0.001	0.778	1.087	0.717	0.845	0.003
zero Presence of children - Yes	0.495	1.137	0.431	0.569	<0.001	0.410	1.186	0.332	0.507	<0.001
zero Region - North of England	0.752	1.096	0.687	0.822	0.002	0.730	1.122	0.639	0.834	0.006
zero Festival - Valentine's Day	0.817	1.157	0.608	1.099	0.165	0.980	1.238	0.643	1.493	0.923
zero Festival - Easter	0.974	1.150	0.732	1.297	0.852	1.133	1.217	0.761	1.686	0.526
zeroFestival - Halloween	1.010	1.253	0.648	1.574	0.966	1.024	1.395	0.533	1.968	0.942
zero Festival - Christmas	2.017	1.202	1.433	2.841	<0.001	3.143	1.250	2.055	4.806	<0.001
zero PPP - 2	0.428	1.122	0.379	0.483	<0.001	0.497	1.166	0.418	0.593	<0.001
zero PPP - 3	0.223	1.128	0.191	0.261	<0.001	0.245	1.183	0.195	0.308	<0.001
zero PPP - 4	0.223	1.144	0.192	0.258	<0.001	0.168	1.208	0.129	0.220	<0.001
zero Interaction Time*Pandemic	0.962	1.016	0.933	0.992	0.015	0.996	1.025	0.954	1.040	0.875
zero Interaction Pandemic*PPP - 2	1.191	1.215	0.858	1.652	0.369	1.202	1.272	0.788	1.836	0.443
zero Interaction Pandemic*PPP - 3	2.544	1.221	1.819	3.556	<0.001	2.026	1.328	1.267	3.240	0.013
zero Interaction Pandemic*PPP - 4	2.877	1.230	2.101	3.939	<0.001	2.359	1.331	1.404	3.966	0.003
Observations	89,382					89,382				

Outcome	Energy purchased from savoury snacks					Enegy purchased from chocolate & confectionery				
Term	Exp. estimate	SE	95%CI low	95%CI high	p value	Exp. estimate	SE	95%CI low	95%CI high	p value
count Constant	0.091	1.066	0.087	0.094	<0.001	0.107	1.060	0.102	0.112	<0.001
count Time	1.001	1.000	1.000	1.001	0.007	1.001	1.000	1.001	1.002	0.001
count Pandemic - during pandemic	1.034	1.048	0.976	1.095	0.483	1.063	1.044	1.001	1.129	0.152
count Season - 2	0.977	1.012	0.957	0.997	0.055	1.001	1.014	0.979	1.024	0.927
count Season - 3	1.077	1.014	1.054	1.100	<0.001	1.047	1.016	1.023	1.071	0.004
count Season - 4	1.093	1.016	1.069	1.119	<0.001	1.202	1.017	1.173	1.232	<0.001
count Age - 45-54 yrs	0.902	1.034	0.884	0.920	0.002	0.941	1.032	0.920	0.962	0.056
count Age - 55-64 yrs	0.826	1.040	0.808	0.845	<0.001	0.878	1.035	0.857	0.900	<0.001
count Age - 65+ yrs	0.790	1.044	0.772	0.809	<0.001	0.840	1.036	0.819	0.861	<0.001
count Sex - male	1.080	1.028	1.063	1.097	0.005	1.046	1.025	1.028	1.064	0.070
count Social grade - C1C2	0.938	1.031	0.921	0.956	0.039	0.909	1.029	0.891	0.927	0.001
count Social grade - AB	0.968	1.035	0.947	0.990	0.350	0.980	1.034	0.957	1.004	0.558
count - Number of adults	0.907	1.013	0.901	0.914	<0.001	0.898	1.014	0.891	0.906	<0.001
count Presence of children - Yes	0.819	1.031	0.804	0.834	<0.001	0.859	1.029	0.841	0.876	<0.001
count Region - North of England	0.914	1.023	0.902	0.927	<0.001	0.945	1.022	0.931	0.960	0.009
count Festival - Valentine's Day	1.094	1.027	1.048	1.141	0.001	1.052	1.029	1.005	1.102	0.072
count Festival - Easter	0.978	1.026	0.935	1.023	0.373	1.214	1.028	1.160	1.271	<0.001
countFestival - Halloween	0.991	1.043	0.930	1.055	0.821	1.129	1.043	1.058	1.205	0.004
count Festival - Christmas	1.258	1.043	1.178	1.344	<0.001	1.191	1.058	1.105	1.284	0.002
count PPP - 2	1.058	1.041	1.030	1.086	0.159	1.085	1.039	1.055	1.116	0.033
count PPP - 3	1.315	1.038	1.282	1.349	<0.001	1.319	1.035	1.283	1.355	<0.001
count PPP - 4	1.936	1.040	1.888	1.985	<0.001	1.901	1.036	1.851	1.953	<0.001
count Interaction Time*Pandemic	1.002	1.003	0.997	1.006	0.536	0.989	1.003	0.984	0.994	<0.001
count Interaction Pandemic*PPP - 2	0.964	1.053	0.908	1.023	0.477	0.999	1.049	0.939	1.063	0.982
count Interaction Pandemic*PPP - 3	0.950	1.050	0.898	1.006	0.299	0.953	1.046	0.898	1.012	0.286
count Interaction Pandemic*PPP - 4	0.850	1.050	0.804	0.899	0.001	0.879	1.044	0.828	0.932	0.003
zero Constant	5.383	1.116	4.960	5.842	<0.001	5.020	1.115	4.628	5.446	<0.001
zero Time	0.999	1.001	0.998	1.000	0.200	0.999	1.001	0.998	1.000	0.078
zero Pandemic - during pandemic	0.772	1.061	0.694	0.857	<0.001	0.579	1.067	0.522	0.643	<0.001
zero Season - 2	0.910	1.022	0.871	0.950	<0.001	0.891	1.023	0.853	0.930	<0.001
zero Season - 3	0.966	1.024	0.925	1.010	0.157	0.933	1.024	0.893	0.974	0.003
zero Season - 4	0.857	1.027	0.816	0.900	<0.001	0.682	1.026	0.650	0.716	<0.001
zero Age - 45-54 yrs	0.917	1.072	0.877	0.959	0.210	0.929	1.068	0.888	0.970	0.259
zero Age - 55-64 yrs	0.886	1.076	0.844	0.929	0.098	0.840	1.073	0.801	0.881	0.013

zero Age - 65+ yrs	1.010	1.079	0.961	1.060	0.901	0.836	1.073	0.796	0.877	0.011
zero Sex - male	1.074	1.054	1.040	1.110	0.172	1.193	1.051	1.156	1.232	<0.001
zero Social grade - C1C2	0.885	1.067	0.851	0.921	0.061	1.042	1.063	1.002	1.084	0.498
zero Social grade - AB	0.982	1.076	0.937	1.029	0.803	1.242	1.073	1.185	1.301	0.002
zero - Number of adults	0.802	1.027	0.788	0.817	<0.001	0.823	1.029	0.809	0.838	<0.001
zero Presence of children - Yes	0.660	1.068	0.633	0.688	<0.001	0.719	1.061	0.690	0.749	<0.001
zero Region - North of England	0.903	1.047	0.876	0.930	0.024	0.790	1.046	0.767	0.813	<0.001
zero Festival - Valentine's Day	0.855	1.046	0.780	0.938	0.001	0.780	1.044	0.712	0.855	<0.001
zero Festival - Easter	1.020	1.047	0.926	1.123	0.664	0.682	1.048	0.618	0.753	<0.001
zeroFestival - Halloween	1.097	1.066	0.959	1.255	0.147	0.945	1.067	0.826	1.082	0.389
zero Festival - Christmas	1.250	1.072	1.090	1.434	0.001	1.811	1.075	1.581	2.074	<0.001
zero PPP - 2	0.429	1.064	0.410	0.449	<0.001	0.418	1.061	0.400	0.437	<0.001
zero PPP - 3	0.236	1.063	0.226	0.247	<0.001	0.254	1.067	0.243	0.266	<0.001
zero PPP - 4	0.145	1.074	0.138	0.152	<0.001	0.155	1.072	0.148	0.162	<0.001
zero Interaction Time*Pandemic	0.988	1.005	0.979	0.998	0.021	1.010	1.005	1.000	1.020	0.043
zero Interaction Pandemic*PPP - 2	1.266	1.074	1.139	1.408	0.001	1.297	1.079	1.167	1.442	0.001
zero Interaction Pandemic*PPP - 3	1.379	1.072	1.238	1.536	<0.001	1.410	1.076	1.266	1.570	<0.001
zero Interaction Pandemic*PPP - 4	1.365	1.076	1.219	1.529	<0.001	1.566	1.087	1.401	1.751	<0.001
Observations	89,382					89,382				

Outcome	Energy purchased from low-sugar soft drinks					Energy from medium-sugar soft drinks				
Term	Exp. estimate	SE	95%CI low	95%CI high	p value	Exp. estimate	SE	95%CI low	95%CI high	p value
count Constant	0.016	1.202	0.014	0.017	<0.001	0.040	1.280	0.032	0.051	<0.001
count Time	1.002	1.001	1.001	1.002	0.160	1.004	1.002	1.001	1.007	0.115
count Pandemic - during pandemic	1.029	1.258	0.896	1.183	0.900	0.658	1.258	0.507	0.855	0.068
count Season - 2	0.921	1.039	0.883	0.960	0.031	0.921	1.090	0.802	1.057	0.338
count Season - 3	1.174	1.043	1.126	1.225	<0.001	0.946	1.102	0.827	1.082	0.567
count Season - 4	1.024	1.048	0.976	1.074	0.613	0.898	1.123	0.779	1.034	0.350
count Age - 45-54 yrs	0.844	1.082	0.811	0.879	0.032	1.246	1.188	1.090	1.425	0.201
count Age - 55-64 yrs	0.864	1.102	0.826	0.904	0.131	1.209	1.160	1.040	1.405	0.200
count Age - 65+ yrs	0.742	1.100	0.707	0.779	0.002	1.002	1.171	0.862	1.164	0.992
count Sex - male	1.107	1.068	1.072	1.143	0.123	1.071	1.103	0.967	1.186	0.486
count Social grade - C1C2	0.800	1.082	0.770	0.831	0.004	0.659	1.215	0.580	0.747	0.032
count Social grade - AB	0.756	1.096	0.721	0.792	0.002	0.691	1.216	0.595	0.802	0.058
count - Number of adults	0.876	1.035	0.864	0.889	<0.001	0.840	1.061	0.800	0.881	0.003
count Presence of children - Yes	0.763	1.070	0.736	0.792	<0.001	1.118	1.184	0.983	1.273	0.506
count Region - North of England	0.869	1.065	0.844	0.895	0.027	0.928	1.096	0.848	1.014	0.413
count Festival - Valentine's Day	1.073	1.077	0.980	1.176	0.339	1.391	1.218	1.047	1.848	0.094
count Festival - Easter	1.086	1.079	0.993	1.188	0.277	0.800	1.131	0.594	1.076	0.070
countFestival - Halloween	1.033	1.203	0.905	1.178	0.861	1.254	1.293	0.813	1.933	0.378
count Festival - Christmas	1.795	1.219	1.551	2.077	0.003	0.640	1.231	0.452	0.906	0.032
count PPP - 2	0.560	1.152	0.523	0.600	<0.001	0.893	1.133	0.801	0.995	0.363
count PPP - 3	0.782	1.151	0.731	0.837	0.082	-				
count PPP - 4	2.434	1.157	2.277	2.602	<0.001	-				
count Interaction Time*Pandemic	1.014	1.007	1.004	1.023	0.054	1.038	1.027	1.007	1.071	0.163
count Interaction Pandemic*PPP - 2	1.150	1.269	0.999	1.323	0.559	1.067	1.262	0.841	1.355	0.779
count Interaction Pandemic*PPP - 3	1.056	1.263	0.922	1.210	0.815	-				
count Interaction Pandemic*PPP - 4	0.695	1.258	0.608	0.795	0.113	-				
zero Constant	13.106	1.162	11.947	14.378	<0.001	88.654	1.280	68.961	113.972	<0.001
zero Time	1.000	1.001	0.999	1.001	0.931	0.996	1.002	0.992	0.999	0.064
zero Pandemic - during pandemic	0.680	1.081	0.599	0.773	<0.001	0.981	1.200	0.731	1.318	0.918
zero Season - 2	0.853	1.024	0.813	0.894	<0.001	0.798	1.078	0.691	0.922	0.003

zero Season - 3	0.783	1.026	0.746	0.822	<0.001	0.845	1.088	0.732	0.976	0.046
zero Season - 4	0.940	1.027	0.891	0.992	0.020	0.731	1.085	0.630	0.847	<0.001
zero Age - 45-54 yrs	0.967	1.100	0.921	1.015	0.725	0.909	1.156	0.788	1.048	0.510
zero Age - 55-64 yrs	1.012	1.110	0.960	1.067	0.910	0.884	1.185	0.758	1.030	0.465
zero Age - 65+ yrs	1.245	1.111	1.179	1.314	0.038	1.049	1.187	0.893	1.232	0.780
zero Sex - male	1.137	1.073	1.097	1.179	0.067	1.201	1.130	1.079	1.337	0.134
zero Social grade - C1C2	1.024	1.086	0.981	1.069	0.776	0.892	1.171	0.782	1.016	0.468
zero Social grade - AB	1.227	1.106	1.165	1.292	0.042	0.929	1.191	0.796	1.084	0.674
zero - Number of adults	0.804	1.039	0.789	0.820	<0.001	0.996	1.074	0.945	1.051	0.960
zero Presence of children - Yes	0.708	1.091	0.677	0.741	<0.001	1.077	1.145	0.941	1.232	0.585
zero Region - North of England	0.704	1.065	0.681	0.727	<0.001	1.269	1.121	1.157	1.392	0.037
zero Festival - Valentine's Day	1.033	1.046	0.934	1.142	0.472	0.896	1.164	0.664	1.208	0.468
zero Festival - Easter	0.950	1.048	0.855	1.055	0.272	1.092	1.159	0.794	1.501	0.552
zeroFestival - Halloween	1.008	1.070	0.871	1.168	0.901	1.521	1.245	0.958	2.416	0.055
zero Festival - Christmas	1.226	1.078	1.055	1.425	0.007	0.917	1.215	0.628	1.338	0.656
zero PPP - 2	0.231	1.096	0.218	0.245	<0.001	0.113	1.125	0.102	0.125	<0.001
zero PPP - 3	0.126	1.099	0.119	0.134	<0.001	-				
zero PPP - 4	0.090	1.101	0.085	0.096	<0.001	-				
zero Interaction Time*Pandemic	0.982	1.005	0.971	0.993	<0.001	0.968	1.016	0.938	0.999	0.046
zero Interaction Pandemic*PPP - 2	1.304	1.093	1.149	1.480	0.003	2.120	1.179	1.664	2.702	<0.001
zero Interaction Pandemic*PPP - 3	1.377	1.092	1.213	1.564	<0.001	-				
zero Interaction Pandemic*PPP - 4	1.612	1.089	1.419	1.833	<0.001	-				
Observations	89,382					89,382				

Outcome	Energy from high-sugar soft drinks					Alcohol volume				
Term	Exp. estimate	SE	95%CI low	95%CI high	p value	Exp. estimate	SE	95%CI low	95%CI high	p value
count Constant	0.091	1.207	0.079	0.105	<0.001	1732.637	1.148	1599.469	1876.892	<0.001
count Time	0.999	1.001	0.997	1.001	0.472	1.002	1.001	1.001	1.003	0.003
count Pandemic - during pandemic	0.367	2.136	0.101	1.334	0.187	1.149	1.089	1.027	1.286	0.103
count Season - 2	0.902	1.056	0.833	0.977	0.060	1.080	1.022	1.045	1.116	<0.001
count Season - 3	1.150	1.076	1.059	1.248	0.056	1.123	1.026	1.086	1.161	<0.001
count Season - 4	0.939	1.069	0.858	1.028	0.345	1.205	1.023	1.162	1.250	<0.001
count Age - 45-54 yrs	0.764	1.113	0.711	0.821	0.012	1.164	1.075	1.122	1.207	0.036
count Age - 55-64 yrs	0.637	1.139	0.590	0.688	0.001	1.039	1.093	1.000	1.080	0.664
count Age - 65+ yrs	0.675	1.195	0.615	0.740	0.027	0.905	1.107	0.870	0.941	0.326
count Sex - male	1.147	1.103	1.079	1.220	0.160	1.106	1.065	1.079	1.133	0.113
count Social grade - C1C2	0.840	1.115	0.786	0.899	0.110	0.821	1.100	0.796	0.846	0.037
count Social grade - AB	0.817	1.115	0.750	0.890	0.063	0.730	1.101	0.705	0.757	0.001
count - Number of adults	0.852	1.054	0.830	0.874	0.002	0.726	1.031	0.717	0.735	<0.001
count Presence of children - Yes	0.695	1.091	0.649	0.743	<0.001	1.017	1.073	0.983	1.052	0.811
count Region - North of England	0.866	1.095	0.819	0.916	0.112	1.254	1.055	1.226	1.283	<0.001
count Festival - Valentine's Day	0.837	1.087	0.705	0.996	0.033	0.920	1.036	0.859	0.985	0.019
count Festival - Easter	0.948	1.093	0.816	1.102	0.549	1.085	1.036	1.014	1.161	0.022
countFestival - Halloween	0.829	1.124	0.650	1.058	0.110	0.872	1.055	0.791	0.961	0.010
count Festival - Christmas	0.854	1.121	0.687	1.060	0.167	1.149	1.062	1.042	1.267	0.021
count PPP - 2	1.216	1.122	1.122	1.317	0.090	1.000	1.072	0.943	1.061	0.998
count PPP - 3	-					2.419	1.069	2.285	2.560	<0.001
count PPP - 4	-					-				
count Interaction Time*Pandemic	1.015	1.011	0.996	1.034	0.176	1.001	1.005	0.993	1.008	0.913
count Interaction Pandemic*PPP - 2	0.828	1.195	0.706	0.972	0.291	1.153	1.092	1.034	1.286	0.107
count Interaction Pandemic*PPP - 3	-					1.017	1.086	0.917	1.127	0.841
count Interaction Pandemic*PPP - 4	-					-				

zero Constant	50.033	1.285	42.483	58.924	<0.001	58.976	1.160	52.873	65.783	<0.001
zero Time	1.002	1.001	0.999	1.004	0.301	0.999	1.001	0.998	1.001	0.408
zero Pandemic - during pandemic	1.363	2.096	0.314	5.909	0.676	0.481	1.097	0.417	0.555	<0.001
zero Season - 2	0.829	1.053	0.755	0.910	<0.001	0.784	1.028	0.745	0.825	<0.001
zero Season - 3	0.874	1.053	0.794	0.963	0.010	0.767	1.031	0.728	0.807	<0.001
zero Season - 4	0.828	1.060	0.745	0.921	0.001	0.610	1.032	0.576	0.646	<0.001
zero Age - 45-54 yrs	1.647	1.167	1.513	1.793	0.001	0.791	1.100	0.751	0.834	0.014
zero Age - 55-64 yrs	1.325	1.202	1.208	1.453	0.127	0.881	1.109	0.832	0.932	0.218
zero Age - 65+ yrs	2.256	1.219	2.026	2.513	<0.001	0.899	1.111	0.849	0.953	0.313
zero Sex - male	0.850	1.136	0.793	0.911	0.202	0.997	1.072	0.960	1.035	0.961
zero Social grade - C1C2	1.199	1.168	1.110	1.296	0.241	0.891	1.088	0.850	0.934	0.171
zero Social grade - AB	1.413	1.185	1.280	1.559	0.041	0.976	1.104	0.923	1.032	0.805
zero - Number of adults	0.910	1.069	0.881	0.939	0.153	0.787	1.038	0.772	0.803	<0.001
zero Presence of children - Yes	1.118	1.160	1.032	1.210	0.455	1.223	1.091	1.165	1.284	0.021
zero Region - North of England	1.357	1.126	1.274	1.444	0.010	0.853	1.067	0.823	0.883	0.013
zero Festival - Valentine's Day	0.951	1.097	0.776	1.165	0.586	0.775	1.051	0.696	0.863	<0.001
zero Festival - Easter	0.693	1.087	0.578	0.830	<0.001	0.968	1.050	0.869	1.079	0.507
zeroFestival - Halloween	1.058	1.138	0.793	1.411	0.664	1.271	1.073	1.090	1.483	0.001
zero Festival - Christmas	0.700	1.146	0.538	0.912	0.009	1.267	1.090	1.085	1.478	0.006
zero PPP - 2	0.062	1.109	0.057	0.068	<0.001	0.162	1.082	0.151	0.174	<0.001
zero PPP - 3	-					0.032	1.089	0.030	0.035	<0.001
zero PPP - 4	-					-				
zero Interaction Time*Pandemic	0.988	1.011	0.967	1.009	0.250	0.983	1.005	0.972	0.994	0.001
zero Interaction Pandemic*PPP - 2	1.921	1.120	1.610	2.293	<0.001	1.477	1.104	1.291	1.689	<0.001
zero Interaction Pandemic*PPP - 3	-					1.940	1.105	1.701	2.213	<0.001
zero Interaction Pandemic*PPP - 4	-					-				
Observations	89,382					89,382				

Outcome			OOH purchasing		
Term	Exp. estimate	SE	95%CI low	95%CI high	p value
count Constant	0.499	1.235	0.459	0.543	0.001
count Time	0.999	1.001	0.998	1.000	0.317
count Pandemic - during pandemic	0.644	1.203	0.523	0.793	0.017
count Season - 2	0.952	1.022	0.913	0.993	0.027
count Season - 3	1.038	1.024	0.994	1.084	0.112
count Season - 4	1.025	1.022	0.976	1.076	0.264
count Age - 45-54 yrs	0.840	1.127	0.808	0.874	0.146
count Age - 55-64 yrs	0.943	1.134	0.904	0.985	0.644
count Age - 65+ yrs	0.915	1.193	0.860	0.973	0.613
count Sex - male	1.288	1.097	1.248	1.329	0.006
count Social grade - C1C2	1.146	1.138	1.098	1.196	0.293
count Social grade - AB	1.235	1.182	1.171	1.302	0.207
count - Number of adults	1.010	1.061	0.991	1.030	0.867
count Presence of children - Yes	1.098	1.125	1.057	1.142	0.425
count Region - North of England	1.067	1.089	1.035	1.099	0.449
count Festival - Valentine's Day	1.016	1.027	0.933	1.107	0.540
count Festival - Easter	0.927	1.043	0.832	1.033	0.068
countFestival - Halloween	0.993	1.039	0.879	1.123	0.862
count Festival - Christmas	0.754	1.064	0.650	0.875	<0.001
count PPP - 2	4.200	1.087	4.047	4.359	<0.001
count PPP - 3	-				
count PPP - 4	-				

count Interaction Time*Pandemic	1.009	1.011	0.994	1.024	0.380
count Interaction Pandemic*PPP - 2	1.037	1.190	0.872	1.233	0.835
count Interaction Pandemic*PPP - 3	-				
count Interaction Pandemic*PPP - 4	-				
zero Constant	<0.001	3.347	<0.001	Inf	<0.001
zero Time	1.093	1.066	1.026	1.164	0.164
zero Pandemic - during pandemic	4012206.126	12.898	<0.001	Inf	<0.001
zero Season - 2	3.909	1.453	2.071	7.376	<0.001
zero Season - 3	20.944	9.676	2.927	149.891	0.180
zero Season - 4	8.927	5.784	2.149	37.085	0.212
zero Age - 45-54 yrs	-				
zero Age - 55-64 yrs	-				
zero Age - 65+ yrs	-				
zero Sex - male	1.013	1.659	0.734	1.397	0.980
zero Social grade - C1C2	0.791	1.675	0.528	1.184	0.649
zero Social grade - AB	1.178	1.905	0.726	1.912	0.799
zero - Number of adults	1.082	1.377	0.887	1.320	0.805
zero Presence of children - Yes	-				
zero Region - North of England	-				
zero Festival - Valentine's Day	-				
zero Festival - Easter	-				
zeroFestival - Halloween	-				
zero Festival - Christmas	-				
zero PPP - 2	3595603.272	2.499	<0.001	Inf	<0.001
zero PPP - 3	-				
zero PPP - 4	-				
zero Interaction Time*Pandemic	0.779	1.101	0.705	0.860	0.009
zero Interaction Pandemic*PPP - 2	<0.001	12.575	<0.001	Inf	<0.001
zero Interaction Pandemic*PPP - 3	-				
zero Interaction Pandemic*PPP - 4	-				
Observations	16,806				
